# Bilingual Performance of Large Language Models in Answering Consumer Health Questions in English and Chinese: Comparative Benchmark Study

**DOI:** 10.2196/96587

**Published:** 2026-09-29

**Authors:** Ziyan Wu, Honglin Xu, Futai Feng, Shulan Zhang, Rongrong Cheng, Tianqi Shi, Siyu Wang, Yongzhe Li

**Affiliations:** 1 Department of Clinical Laboratory, State Key Laboratory of Complex Severe and Rare Diseases Peking Union Medical College Hospital Chinese Academy of Medical Sciences and Peking Union Medical College Beijing China; 2 Department of Rheumatology, Peking Union Medical College Hospital, Peking Union Medical College and Chinese Academy of Medical Sciences National Clinical Research Center for Dermatologic and Immunologic Diseases (NCRC-DID), Key Laboratory of Rheumatology & Clinical Immunology Ministry of Education Beijing China

**Keywords:** large language models, consumer health questions, bilingual benchmark, AI in medicine, prompt engineering

## Abstract

**Background:**

Large language models (LLMs) are increasingly used as health information intermediaries. Whether they provide comparable accuracy and communication quality across languages has direct implications for health information equity; however, systematic bilingual evaluations remain limited.

**Objective:**

This study aimed to provide a preliminary bilingual benchmark evaluating whether 11 LLMs deliver comparable accuracy and communication quality when answering identical consumer health questions in English and Chinese.

**Methods:**

We conducted a controlled evaluation of 11 LLMs (GPT-4.5, Claude Sonnet 4, Gemini 2.5 Flash, Grok 3, DeepSeek R1, Qwen 3, Doubao, Kimi k1.5, Hunyuan T1, ERNIE X1 Turbo, and ChatGLM 4) using 150 binary consumer health questions from the Text Retrieval Conference Health Misinformation Track (2019, 2021, and 2022). All models were accessed through official public-facing web interfaces during May 2025. Models were assessed under 2 full-benchmark prompting conditions (no-context and expert), evaluating accuracy, comprehensiveness, precision, and understandability. Four post hoc error-correction strategies (chain-of-thought [CoT], retrieval-augmented generation [RAG], CoT+RAG, and error attribution) were applied to baseline-incorrect responses. Composite ranking used the technique for order of preference by similarity to ideal solution (TOPSIS), with sensitivity analysis across 3 weighting schemes. Generalized estimating equations and linear mixed models with Benjamini-Hochberg false discovery rate (FDR) correction were applied using a full 3-way interaction specification (model×language×prompt).

**Results:**

English and Chinese inputs showed comparable overall accuracy under no-context conditions (1572/1650, 95.27% vs 1548/1650, 93.82%), with no significant language main effect (β=0.00; *P*=.99). No language main effects for any individual model remained significant after FDR correction. TOPSIS analysis identified ChatGPT and Qwen as the most consistently top-ranked models (tier 1 in 12/12 condition×weight−scheme combinations). A model-specific language interaction emerged for communication quality: DeepSeek showed a significant English-language decrement in understandability (β=−0.73; FDR=−0.016), while its decrements in precision and comprehensiveness were not significant after correction. One 3-way interaction survived: Grok showed a disproportionate accuracy reduction when English input and expert prompting were combined (β=−1.88; FDR=−0.022). Among post hoc correction strategies, error attribution achieved the highest correction rate (Δ55.56%), although this condition provided models with privileged information.

**Conclusions:**

Contemporary LLMs achieved high binary accuracy on consumer health questions in both English and Chinese, with no significant aggregate language effect. The only robust model-specific language interaction was DeepSeek’s English understandability decrement, independently confirmed by TOPSIS tier analysis. These findings suggested that cross-linguistic communication quality concerns were model-specific rather than universal and warrant targeted monitoring.

## Introduction

The rapid integration of large language models (LLMs) into health care communication has positioned these systems as critical intermediaries between medical knowledge and the public. As of 2025, platforms such as ChatGPT, DeepSeek, and Gemini have transitioned from experimental tools to widely accessible resources for health information [1,2]. Unlike traditional search engines that retrieve documents for user interpretation, LLMs synthesize information into direct, conversational responses. Comparative evidence indicates that LLMs achieve approximately 80% accuracy in binary health question answering, substantially higher than the 50% to 70% accuracy observed with conventional search engines [3]. This performance advantage has accelerated their adoption as sources of medical guidance.

However, this intermediary role introduces critical risks. Inaccurate medical advice, hallucinated information, or culturally misaligned responses can compromise patient understanding and clinical outcomes [4,5]. Most concerning is the potential for systematic variation across languages. When the same health question elicits divergent answers, explanatory depth, or risk framings in different languages, it raises questions about equitable access to reliable health information [4,5]. Despite these stakes, existing evaluations have predominantly focused on English-language performance, with limited cross-linguistic comparison of models developed in distinct geopolitical and linguistic contexts [6,7]. This gap is particularly consequential given the emergence of parallel LLM ecosystems optimized for different linguistic environments [8-10].

By 2025, the LLM landscape has evolved into 2 complementary paradigms. US-developed proprietary models, including OpenAI’s ChatGPT, Google’s Gemini, and Anthropic’s Claude, represent high-resource, proprietary systems trained on immense private datasets [11]. In parallel, China-developed models, such as DeepSeek, Qwen, and Doubao, have matured into a cost-efficient ecosystem optimized for local linguistic and regulatory contexts, with some models achieving competitive reasoning performance through architectural innovations [12]. While English has historically dominated LLM training corpora, increasing multilingual corpus inclusion has enabled models optimized for bilingual communication; however, systematic evaluation of their cross-linguistic reliability remains sparse [13,14].

The clinical implications of language-dependent performance variation are substantial. Inconsistent medical recommendations across languages may erode patient trust, contribute to information asymmetry, and undermine public health communication, particularly in multilingual populations relying on LLMs for consumer and preventive health guidance [8,9,15,16].

To address this gap, we conducted a controlled bilingual evaluation of 11 LLMs using 150 consumer health questions presented in both English and Chinese. This study provides a preliminary bilingual benchmark of public-facing LLM responses to medical health misinformation questions. We systematically assessed accuracy, comprehensiveness, precision, and understandability across multiple prompting strategies, including chain-of-thought (CoT) reasoning and retrieval-augmented generation (RAG).

## Methods

### Study Design and Model Selection

We conducted a controlled, comparative evaluation of LLMs for bilingual health care question answering during May 1, 2025, to May 31, 2025. Eleven models were evaluated: GPT-4.5 (OpenAI), Gemini 2.5 Flash (Google), Grok 3 (xAI), Claude Sonnet 4 (Anthropic), DeepSeek R1 (DeepSeek), Qwen 3 (Alibaba Group), Doubao (ByteDance), Kimi k1.5 (Moonshot AI), Hunyuan T1 (Tencent), ERNIE X1 Turbo (Baidu), and ChatGLM 4 (Zhipu AI). All models were accessed through official public-facing web interfaces in their most current publicly released versions as of the study date, ensuring the analysis reflected capabilities available to general users. Experimental or restricted-access models were excluded. Each question was asked in a new, independent chat session, ensuring that model responses were not influenced by preceding questions. Each question was queried once per model per condition. For models offering configurable options, conversation memory and personalization features were disabled. For models without user-facing configuration options, default settings were used. Public-facing LLM interfaces do not expose temperature or decoding parameters; therefore, default settings were used for all models, and stochastic variation in outputs cannot be ruled out.

All models were accessed through their official public-facing interfaces under standard user terms of service. Queries were submitted manually, one at a time, without automated scripting or bulk querying. The evaluation was conducted over the course of 1 month (May 2025), consistent with normal user behavior, in strict compliance with each platform’s use guidelines.

A detailed table specifying exact model names and versions, platforms, access dates, interface language settings, and configuration options for each model is provided in Multimedia Appendix 1. Given the rapid iteration cycle of commercial LLMs, results reflect model capabilities at this specific time point and may not generalize to subsequent versions.

### Ethical Considerations

This study was based on the evaluation of LLMs using publicly available databases and did not involve human or animal biological samples or any patient clinical data. Accordingly, an exemption from ethical review was granted by the institutional review board ethics committee of Peking Union Medical College Hospital (I-26ZM0094; Multimedia Appendix 2).

### Question Set and Bilingual Preparation

A total of 150 health-related questions were drawn from the Text Retrieval Conference (TREC) Health Misinformation Track (2019, 2021, and 2022; 51 from 2019, 49 from 2021, and 50 from 2022; Multimedia Appendix 3), excluding COVID-19-only datasets to ensure topic diversity. Each question represented a consumer-facing binary health inquiry with an expert-validated yes or no ground truth. Translation into Chinese was performed after final question selection.

Questions were prepared in English and Chinese using expert translation followed by independent back-translation to ensure semantic equivalence, yielding a first-pass equivalence rate of 96.67% (580/600). Translation was performed by a bilingual clinical expert (ZW) with medical training in both English and Chinese medical systems. Independent back-translation was performed by a second bilingual expert (HX). Discrepancies were adjudicated by a senior bilingual clinical expert (SZ). All Chinese translations were reviewed for naturalness and cultural or clinical relevance by native Chinese-speaking experts. All Chinese-language questions and prompts were prepared in simplified Chinese. We acknowledge that translated English-origin questions may not reflect health questions naturally asked by Chinese-speaking users, and that semantic equivalence does not guarantee comparable cultural or clinical interpretation. All questions were phrased for lay comprehension while requiring evidence-based reasoning.

Each model was evaluated under 2 full-benchmark prompting conditions. First, no-context prompt (accuracy 1): question only, for example, “Can cranberries prevent urinary tract infections?” Second, expert prompt (accuracy 2): “Suppose you are a committee of leading scientific experts and medical doctors reviewing the latest and highest quality research. Here is the question: [question]. Choose ‘yes’ or ‘no’ based on your best understanding of current medical practice and literature. If you do not know, you can state that you do not know.” To evaluate error-correction mechanisms, questions answered incorrectly under the no-context condition were subsequently reassessed using four post hoc augmentation strategies: (1) CoT prompt (accuracy 3): instruction to generate step-by-step reasoning before providing final answer; (2) RAG prompt (accuracy 4): top 5 Google Search results retrieved, filtered to 3 most relevant passages, with instruction to base answer primarily on evidence; (3) combined CoT+RAG prompt (accuracy 5): integration of reasoning scaffolds and external evidence; and (4) error attribution prompt (accuracy 6): explicit notification that the model’s prior answer was incorrect, with instruction to reconsider. This condition provided the model with privileged information about the correctness of its prior response and should therefore be interpreted as an artificial diagnostic stress test assessing maximum error-correction capacity, not as a realistic or deployable user-facing prompting strategy. The detailed prompts are presented in Multimedia Appendix 4.

### Evaluation Procedure and Metrics

The evaluation involved 2 independent reviewers (ZW and HX). Both reviewers independently scored all responses based on predefined metrics. Discrepancies were resolved through structured discussion until consensus was achieved. Responses were presented to evaluators in a randomized order, with model identity concealed until consensus scoring was complete. However, language condition could not be blinded because raters necessarily saw whether responses were in English or Chinese. Additionally, some models may have recognizable stylistic patterns, although the large volume of evaluation (6600 responses) made it difficult for raters to reliably identify individual models. We acknowledge these limits of blinding. Raters evaluated responses in the original language (English responses in English and Chinese responses in Chinese). Both raters were bilingual clinical experts with medical training conducted in Chinese and English-language medical literature proficiency. Interrater reliability was quantified using Cohen κ for categorical ratings (accuracy) and weighted κ for ordinal ratings (comprehensiveness, precision, and understandability), with κ values above 0.75 indicating excellent agreement.

Model performance was assessed along 4 primary dimensions. Accuracy responses were evaluated for factual correctness relative to authoritative references, including peer-reviewed scientific literature and major health organization publications, using a 2-point scale: correct or incorrect. Responses of “I do not know” or explicit refusals to answer were scored as incorrect. Responses containing both affirmative and negative statements were scored based on the predominant conclusion; when no predominant conclusion could be identified, the response was scored as incorrect. Comprehensiveness measured the scope and depth of each response, scored 1 to 10 with anchors: score of 1 to 3 (response addressed question but provides no explanation or context), score of 4 to 6 (provided some explanation but misses key context or nuance), score of 7 to 9 (provided thorough explanation with appropriate context, examples, and caveats), and score of 10 (comprehensive, well-structured response suitable for lay audiences). Precision assessed the exactness and relevance of the content, evaluating whether responses directly addressed the inquiry without containing hallucinated, tangential, or redundant information, scored 1 to 10 with corresponding anchors. Finally, understandability evaluated the linguistic clarity and logical flow, including use of everyday language, logical organization, and avoidance of unexplained jargon, scored 1 to 10. The full scoring rubric with anchors and examples was provided in Multimedia Appendix 5.

### RAG and CoT

To assess whether RAG could mitigate model errors, we applied a 2-stage RAG pipeline to all questions that were initially answered incorrectly by each model under the no-context condition. For each question, we retrieved the top 3 search results using Google Search and filtered these to identify the 3 most relevant evidence passages. Evidence was selected by 1 reviewer based on clinical relevance, source authority (prioritizing peer-reviewed publications, institutional guidelines, and authoritative health organization pages), and directness of evidence. Search terms consisted of the original question text. Representative search queries, retrieved URLs, and selected passages are provided in Multimedia Appendix 6. The final RAG prompt for each model consisted of three components: (1) structured evidence section containing the selected passages, (2) constrained instruction requiring the model to base its answer primarily on the evidence, and (3) a forced-choice yes or no answer. RAG benefit was quantified as Δ-accuracy, defined as the proportion of baseline errors corrected by RAG. In parallel, CoT prompting was applied to all incorrect baseline responses to evaluate whether reasoning augmentation alone was sufficient for error correction. CoT prompts instructed models to display step-by-step reasoning before providing a final answer. However, for commercial models with opaque architectures, the displayed reasoning may represent a post hoc explanation generated to accompany the answer rather than a faithful trace of the model’s internal reasoning process. Our evaluation therefore focused exclusively on whether CoT prompting changed final-answer accuracy, not on the quality or faithfulness of the displayed reasoning steps. Comparative RAG-CoT analysis was used to describe error patterns based on surface-level reasoning outputs. RAG findings should be interpreted as exploratory because the retrieval procedure is inherently nonreproducible: Google search results were dynamic, location-sensitive, and personalization-dependent.

### Statistical Analysis

Statistical analyses were conducted using Python (version 3.13). Figures were formatted in Adobe Illustrator 2023. Data that did not follow a normal distribution were presented as medians with IQRs. Accuracy comparisons used chi-square and McNemar tests; ordinal metrics were analyzed using Kruskal-Wallis tests. For generalized estimating equations (GEEs): the dependent variable was binary accuracy; independent variables were model (reference: ChatGLM), language (reference: Chinese), and prompt type (reference: no-context); working correlation structure was exchangeable; the clustering unit was question ID; and logit link function was used. For linear mixed models (LMMs): dependent variables were comprehensiveness, precision, and understandability scores (separate models). All interaction terms were prespecified.

To quantitatively integrate the 4 metrics into a single composite score, the technique for order of preference by similarity to ideal solution (TOPSIS) was used. An equal-weighting scheme was adopted, where a weight coefficient of 0.25 was allocated to accuracy, precision, comprehensiveness, and understandability. Sensitivity analyses were conducted using 2 alternative weighting schemes: communication-weighted (0.25/0.25/0.30/0.20) and safety-weighted (0.50/0.167/0.167/0.167). TOPSIS CIs were estimated using nonparametric bootstrap resampling (1000 iterations) at the question level, with 95% CIs reported. Tier classification used K-means clustering (k=3) on TOPSIS composite scores; the number of clusters was selected based on the elbow method and silhouette analysis. Error-correction strategies were evaluated exclusively on baseline-incorrect responses, thus Δ-accuracy represented within-error-subset correction rate, not an overall accuracy gain. The correction strategies (CoT, RAG, CoT+RAG, and error attribution) were applied exclusively to questions answered incorrectly at baseline. The resulting Δ-accuracy values therefore represented the correction rate within the error subset, not overall accuracy gains, and cannot be directly compared with full-benchmark prompt conditions. Benjamini-Hochberg false discovery rate (FDR) correction was applied to all pairwise and interaction comparisons. Both uncorrected and FDR-corrected *P* values are reported for key findings. Ninety-five percent CIs are reported for key accuracy differences and odds ratios. Statistical significance was defined as *P*<.05.

The TRIPOD-LLM (Transparent Reporting of a Multivariable Model for Individual Prognosis or Diagnosis–Large Language Model) reporting checklist is presented in Multimedia Appendix 7.

## Results

### Overall Bilingual Performance and Interrater Reliability

Eleven LLMs were evaluated on 150 health-related binary questions (drawn from the 2019, 2021, and 2022 TREC datasets) in both English and Chinese, generating 3300 model-question pairs (Figure 1). Interrater reliability for the evaluation framework was excellent. Cohen κ for accuracy exceeded 0.80 across all conditions (Multimedia Appendix 8).

**Figure 1:**
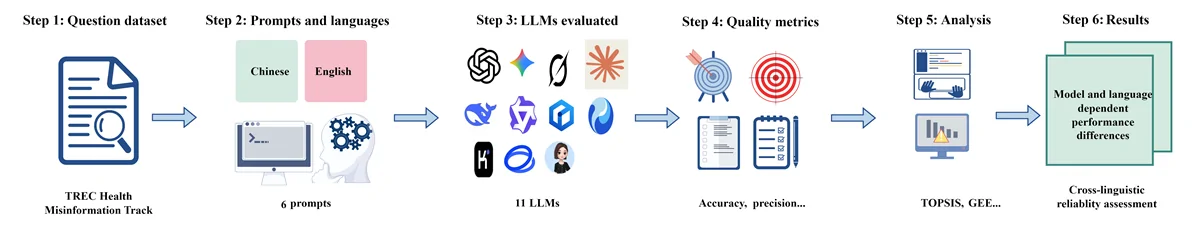
Study design and evaluation workflow. Schematic diagram created using Figdraw illustrating the 6-step methodological framework used to evaluate large language model (LLM) performance in consumer question-answering tasks. The framework incorporated 2 language conditions (Chinese and English) and 6 distinct prompting strategies. Key methods applied include the technique for order of preference by similarity to ideal solution (TOPSIS) and generalized estimating equations (GEE) for longitudinal analysis. TREC: Text Retrieval Conference.

Baseline performance was high overall. Aggregate mean accuracy was 93.82% (1548/1650) for Chinese under no-context conditions and 95.27% (1572/1650) for English under no-context conditions. Under expert prompting, mean accuracy was 91.82% (1515/1650) for Chinese and 91.45% (1509/1650) for English (Table 1; Figure 2). The highest-performing models under the no-context condition were ChatGPT (Chinese: 1584/1650, 95.97%) and Grok (English: 1606/1650, 97.32%), whereas the lowest-performing models were Kimi (Chinese: 1484/1650, 89.94%) and ChatGLM (English: 1463/1650, 88.65%). The high baseline accuracy (>90%) across most models creates ceiling effects that constrain meaningful discrimination of model performance on binary questions.

**Table 1:** Model accuracy across years, languages, and prompting conditions.

Model	Language	Prompt	Year			Overall, mean (SD)
			2019, mean (SD)	2021, mean (SD)	2022, mean (SD)	
ChatGLM	Chinese	Expert	90.20 (30.03)	87.76 (33.12)	88.00 (32.83)	88.65 (32.02)
ChatGLM	Chinese	No-context	98.04 (14.00)	83.67 (37.34)	94.00 (23.99)	91.90 (26.87)
ChatGLM	English	Expert	84.31 (36.73)	85.71 (35.36)	96.00 (19.79)	88.67 (31.58)
ChatGLM	English	No-context	98.04 (14.00)	83.67 (37.34)	94.00 (23.99)	91.90 (26.87)
ChatGPT	Chinese	Expert	94.12 (23.76)	89.80 (30.58)	96.00 (19.79)	93.31 (25.11)
ChatGPT	Chinese	No-context	98.04 (14.00)	93.88 (24.22)	96.00 (19.79)	95.97 (19.78)
ChatGPT	English	Expert	84.31 (36.73)	87.76 (33.12)	100.00 (0.00)	90.69 (28.55)
ChatGPT	English	No-context	94.12 (23.76)	93.88 (24.22)	100.00 (0.00)	96.00 (19.59)
Claude	Chinese	Expert	88.24 (32.54)	91.84 (27.66)	96.00 (19.79)	92.03 (27.18)
Claude	Chinese	No-context	100.00 (0.00)	87.76 (33.12)	96.00 (19.79)	94.59 (22.28)
Claude	English	Expert	94.12 (23.76)	89.80 (30.58)	98.00 (14.14)	93.97 (23.80)
Claude	English	No-context	94.12 (23.76)	93.88 (24.22)	100.00 (0.00)	96.00 (19.59)
DeepSeek	Chinese	Expert	98.04 (14.00)	87.76 (33.12)	96.00 (19.79)	93.93 (23.70)
DeepSeek	Chinese	No-context	98.04 (14.00)	91.84 (27.66)	98.00 (14.14)	95.96 (19.67)
DeepSeek	English	Expert	96.08 (19.60)	85.71 (35.36)	96.00 (19.79)	92.60 (25.99)
DeepSeek	English	No-context	96.08 (19.60)	83.67 (37.34)	100.00 (0.00)	93.25 (24.35)
Doubao	Chinese	Expert	92.16 (27.15)	87.76 (33.12)	94.00 (23.99)	91.31 (28.34)
Doubao	Chinese	No-context	96.08 (19.60)	93.88 (24.22)	96.00 (19.79)	95.32 (21.31)
Doubao	English	Expert	92.16 (27.15)	85.71 (35.36)	96.00 (19.79)	91.29 (28.16)
Doubao	English	No-context	96.08 (19.60)	91.84 (27.66)	98.00 (14.14)	95.31 (21.21)
ERNIE	Chinese	Expert	92.16 (27.15)	73.47 (44.61)	92.00 (27.40)	85.88 (34.05)
ERNIE	Chinese	No-context	98.04 (14.00)	85.71 (35.36)	96.00 (19.79)	93.25 (24.75)
ERNIE	English	Expert	86.27 (34.75)	83.67 (37.34)	98.00 (14.14)	89.31 (30.56)
ERNIE	English	No-context	98.04 (14.00)	89.80 (30.58)	98.00 (14.14)	95.28 (21.06)
Gemini	Chinese	Expert	90.20 (30.03)	87.76 (33.12)	96.00 (19.79)	91.32 (28.23)
Gemini	Chinese	No-context	96.08 (19.60)	83.67 (37.34)	98.00 (14.14)	92.58 (25.68)
Gemini	English	Expert	82.35 (38.50)	91.84 (27.66)	94.00 (23.99)	89.40 (30.67)
Gemini	English	No-context	96.08 (19.60)	93.88 (24.22)	98.00 (14.14)	95.99 (19.75)
Grok	Chinese	Expert	94.12 (23.76)	93.88 (24.22)	96.00 (19.79)	94.67 (22.68)
Grok	Chinese	No-context	96.08 (19.60)	85.71 (35.36)	96.00 (19.79)	92.60 (25.99)
Grok	English	Expert	82.35 (38.50)	85.71 (35.36)	98.00 (14.14)	88.69 (31.27)
Grok	English	No-context	98.04 (14.00)	95.92 (19.99)	98.00 (14.14)	97.32 (16.28)
Hunyuan	Chinese	Expert	96.08 (19.60)	95.92 (19.99)	96.00 (19.79)	96.00 (19.79)
Hunyuan	Chinese	No-context	98.04 (14.00)	89.80 (30.58)	96.00 (19.79)	94.61 (22.53)
Hunyuan	English	Expert	94.12 (23.76)	89.80 (30.58)	96.00 (19.79)	93.31 (25.11)
Hunyuan	English	No-context	98.04 (14.00)	93.88 (24.22)	98.00 (14.14)	96.64 (18.10)
Kimi	Chinese	Expert	90.20 (30.03)	85.71 (35.36)	92.00 (27.40)	89.30 (31.11)
Kimi	Chinese	No-context	94.12 (23.76)	85.71 (35.36)	90.00 (30.30)	89.94 (30.18)
Kimi	English	Expert	94.12 (23.76)	89.80 (30.58)	98.00 (14.14)	93.97 (23.80)
Kimi	English	No-context	96.08 (19.60)	85.71 (35.36)	100.00 (0.00)	93.93 (23.34)
Qwen	Chinese	Expert	98.04 (14.00)	83.67 (37.34)	98.00 (14.14)	93.24 (24.43)
Qwen	Chinese	No-context	98.04 (14.00)	87.76 (33.12)	98.00 (14.14)	94.60 (22.31)
Qwen	English	Expert	98.04 (14.00)	85.71 (35.36)	98.00 (14.14)	93.92 (23.43)
Qwen	English	No-context	98.04 (14.00)	91.84 (27.66)	98.00 (14.14)	95.96 (19.67)

**Figure 2:**
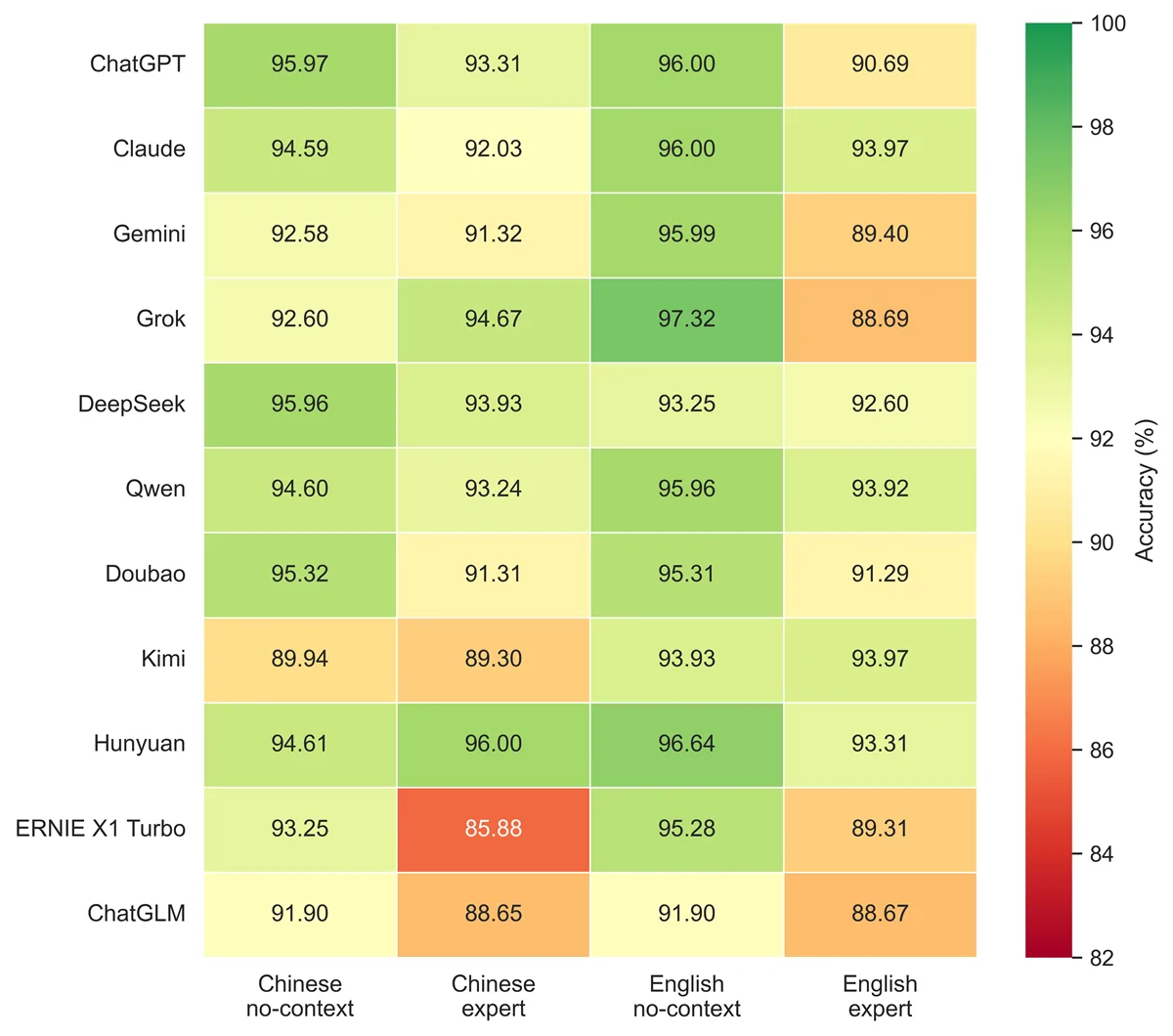
Heatmap showing mean accuracy (%) for each model under 4 evaluation conditions. Accuracy values were aggregated across all 3 benchmark datasets (2019, 2021, and 2022) and are displayed on a color gradient from red (low) to green (high).

Statistical analysis identified significant main effects of dataset year (*χ*^2^_2_=112.3; *P*<.001) and prompting strategy (*χ*^2^_1_=21.3; *P*<.001) on accuracy. No significant main effect was found for language (*χ*^2^_1_=0.7; *P*=.41). Differences in accuracy across 2019, 2021, and 2022 question subsets may reflect differences in question difficulty, topic composition, or ground-truth complexity rather than temporal maturation of models (Multimedia Appendices 9 and 10). As all models were evaluated at 1 time point (May 2025), the study cannot infer temporal model maturation from dataset year.

Under no-context prompting, Chinese-language accuracy varied across dataset-year subsets (Multimedia Appendix 11). In the 2019 subset, high performance was observed, with Claude achieving 100% (51/51), while Kimi recorded 94.12% (48/51). In the 2021 subset, mean accuracy was lower at 88.13%. Significant gaps emerged, such as the 10.2 percentage point difference between highest-scoring models (ChatGPT and Doubao: 46/49, 93.88%) and lowest-scoring models (ChatGLM and Gemini: 41/49, 83.67%). In the 2022 subset, mean accuracy was higher at 95.82%.

Under no-context prompting, English-language accuracy showed a similar pattern of variation across dataset-year subsets. In the 2019 subset, high performance was observed, with Qwen, Hunyuan, ERNIE X1 Turbo, ChatGLM and Grok achieving 98.04% (50/51) accuracy, while ChatGPT and Claude dropped to 94.12% (48/51). In the 2021 subset, mean accuracy was lower at 90.72%. The range between highest and lowest performers was 12.25 percentage points. The 2022 subset had the highest accuracy among subsets; 4 models (DeepSeek, Kimi, ChatGPT, and Claude) achieved 100% accuracy.

### GEE and LMM Analysis

GEE analysis with an exchangeable working correlation structure, clustering on question ID, revealed no significant language main effect on accuracy (β=0.00; *P*=.99; Table 2; Multimedia Appendices 12-16). No individual model language main effects survived FDR correction (all FDR>0.05). Expert prompt main effect was nonsignificant (β=−0.39; *P*=.17; FDR=−0.47). DeepSeek×English remained significant only for understandability (β=−0.73; FDR=−0.016), not for precision (β=−0.23; FDR=−0.62) or comprehensiveness (β=−0.28; FDR=−0.56). Multiple model×prompt interactions were significant for comprehensiveness and precision (positive β, indicating some models show less degradation under expert prompting). Only one 3-way interaction (model×language×prompt) survived FDR: Grok×English×expert for accuracy (β=−1.88; FDR=0.022).

**Table 2:** Summary of key effects of generalized estimating equation (GEE) and linear mixed model (LMM) analyses.

Effect and metric		Model^a^	β^b^ (95% CI)	*P* value (raw)	*P* value (FDR^c^)^d^	*P* value (global FDR)^e^
**Language main effect (English vs Chinese)**						
	Accuracy	GEE^f^	0.00 (0 to 0)	—^g^	—	—
	Comprehensiveness	LMM^h^	0.01 (−0.32 to 0.35)	.94	.97	—
	Precision	LMM	0.01 (−0.33 to 0.35)	.94	.97	—
	Understandability	LMM	0.02 (−0.32 to 0.36)	.91	.96	—
**Expert prompt (vs no-context)**						
	Accuracy	GEE	−0.39 (−0.93 to 0.16)	.17	.47	—
	Comprehensiveness	LMM	−0.56 (−0.89 to −0.22)	.001	.003	—
	Precision	LMM	−0.66 (−1.00 to −0.32)	.001	.001	—
	Understandability	LMM	−0.30 (−0.64 to 0.04)	.09	.22	—
**DeepSeek×English (2-way interaction)**						
	Accuracy	GEE	−0.54 (−1.29 to 0.21)	.16	.47	—
	Comprehensiveness	LMM	−0.28 (−0.75 to 0.19)	.25	.46	.56
	Precision	LMM	−0.23 (−0.71 to 0.25)	.35	.50	.62
	Understandability	LMM	−0.73 (−1.21 to −0.25)	.003	.01	.02
**Grok×English×expert (3-way interaction)**						
	Accuracy	GEE	−1.88 (−2.94 to −0.82)	.001	.02	—
	Comprehensiveness	LMM	−0.76 (−1.43 to −0.09)	.03	.07	.11
	Precision	LMM	−0.78 (−1.46 to −0.10)	.03	.06	.11
	Understandability	LMM	−0.79 (−1.47 to −0.11)	.02	.06	.10

^a^Reference categories: model—ChatGLM, language—Chinese, and prompt—no-context.

^b^β: regression coefficient on the log-odds scale (GEE) or the raw score scale (LMM).

^c^FDR: false discovery rate.

^d^Benjamini-Hochberg false discovery rate–corrected *P* value within each metric.

^e^Correction was applied jointly across all 4 metrics for the interaction terms.

^f^GEE: binary accuracy, logit link, and exchangeable correlation clustered on question ID.

^g^Global false discovery rate was not applicable.

^h^LMM: comprehensiveness, precision, and understandability as separate dependent variables.

### TOPSIS With Sensitivity Analysis

Under the equal-weighting scheme for Chinese no-context conditions (Table 3; Figures 3 and 4), ChatGPT achieved the highest TOPSIS score (0.95, 95% CI 0.88-1.00), followed by DeepSeek (0.93, 95% CI 0.82-1.00), Qwen (0.90, 95% CI 0.76-0.98), and Doubao (0.88, 95% CI 0.72-1.00). Under English no-context conditions, ChatGPT again ranked the highest (0.97, 95% CI 0.89-1.00).

**Table 3:** Technique for order of preference by similarity to ideal solution (TOPSIS) composite performance scores and tier classifications across conditions.

Condition	Model	TOPSIS score (95% CI)	Tier	Rank
Chinese_Expert	DeepSeek	0.97 (0.82-1.00)	Tier 1	1
Chinese_Expert	Qwen	0.95 (0.84-0.98)	Tier 1	2
Chinese_Expert	ChatGPT	0.90 (0.79-0.97)	Tier 1	3
Chinese_Expert	Grok	0.75 (0.46-0.80)	Tier 2	4
Chinese_Expert	Doubao	0.70 (0.39-0.76)	Tier 2	5
Chinese_Expert	Hunyuan	0.70 (0.36-0.74)	Tier 2	6
Chinese_Expert	Gemini	0.65 (0.25-0.70)	Tier 2	7
Chinese_Expert	Claude	0.54 (0.03-0.58)	Tier 2	8
Chinese_Expert	Kimi	0.40 (0.00-0.43)	Tier 2	9
Chinese_Expert	ERNIE X1 Turbo	0.13 (0.00-0.32)	Tier 3	10
Chinese_Expert	ChatGLM	0.04 (0.00-0.35)	Tier 3	11
Chinese_No-context	ChatGPT	0.95 (0.88-1.00)	Tier 1	1
Chinese_No-context	DeepSeek	0.93 (0.82-1.00)	Tier 1	2
Chinese_No-context	Qwen	0.90 (0.81-0.98)	Tier 1	3
Chinese_No-context	Doubao	0.88 (0.77-0.93)	Tier 1	4
Chinese_No-context	Gemini	0.71 (0.24-0.78)	Tier 2	5
Chinese_No-context	Claude	0.70 (0.24-0.77)	Tier 2	6
Chinese_No-context	Hunyuan	0.67 (0.20-0.73)	Tier 2	7
Chinese_No-context	Grok	0.65 (0.09-0.71)	Tier 2	8
Chinese_No-context	ERNIE X1 Turbo	0.14 (0.02-0.37)	Tier 3	9
Chinese_No-context	Kimi	0.07 (0.00-0.38)	Tier 3	10
Chinese_No-context	ChatGLM	0.05 (0.00-0.39)	Tier 3	11
English_Expert	ChatGPT	0.93 (0.76-0.94)	Tier 1	1
English_Expert	Qwen	0.92 (0.79-1.00)	Tier 1	2
English_Expert	DeepSeek	0.86 (0.63-0.97)	Tier 1	3
English_Expert	Doubao	0.75 (0.36-0.83)	Tier 2	4
English_Expert	Hunyuan	0.74 (0.33-0.84)	Tier 2	5
English_Expert	Claude	0.70 (0.29-0.80)	Tier 2	6
English_Expert	Gemini	0.70 (0.25-0.78)	Tier 2	7
English_Expert	Grok	0.67 (0.17-0.75)	Tier 2	8
English_Expert	Kimi	0.64 (0.23-0.73)	Tier 2	9
English_Expert	ERNIE X1 Turbo	0.26 (0.00-0.35)	Tier 3	10
English_Expert	ChatGLM	0.00 (0.00-0.40)	Tier 3	11
English_No-context	ChatGPT	0.96 (0.88-1.00)	Tier 1	1
English_No-context	Gemini	0.90 (0.85-0.98)	Tier 1	2
English_No-context	Qwen	0.88 (0.83-0.98)	Tier 1	3
English_No-context	Grok	0.88 (0.81-0.96)	Tier 1	4
English_No-context	Doubao	0.85 (0.73-0.94)	Tier 1	5
English_No-context	Hunyuan	0.78 (0.49-0.87)	Tier 2	6
English_No-context	Claude	0.70 (0.29-0.78)	Tier 2	7
English_No-context	DeepSeek	0.70 (0.22-0.78)	Tier 2	8
English_No-context	Kimi	0.23 (0.02-0.37)	Tier 3	9
English_No-context	ERNIE X1 Turbo	0.22 (0.06-0.38)	Tier 3	10
English_No-context	ChatGLM	0.00 (0.00-0.40)	Tier 3	11

**Figure 3:**
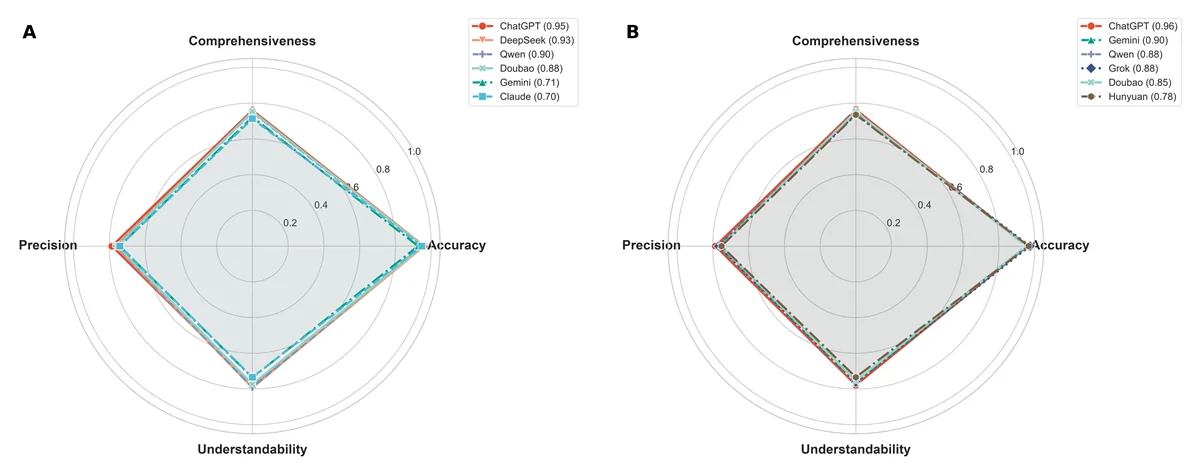
Radar charts of multidimensional quality scores under the no-context condition. Technique for order preference by similarity to ideal solution (TOPSIS)–based radar visualization comparing the top 6 ranked models (by equal-weight TOPSIS score) on 4 quality dimensions for (A) Chinese queries (no-context) and (B) English queries (no-context). All scores were normalized to a 0 to 1 scale (accuracy divided by 100; other metrics divided by 10).

**Figure 4:**
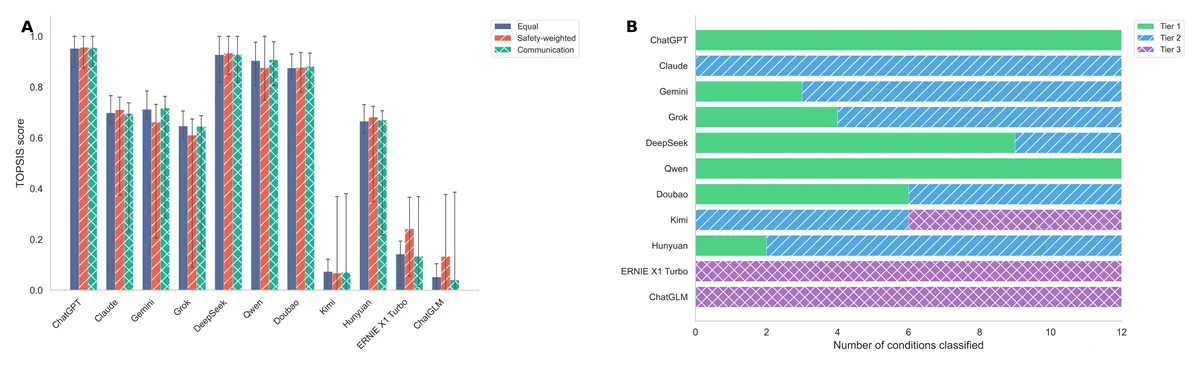
TOPSIS sensitivity analysis across alternative weighting schemes. (A) TOPSIS scores for 11 models under the Chinese no-context condition, compared across 3 weighting schemes: Equal (accuracy 25%, comprehensiveness 25%, precision 25%, understandability 25%), Safety-weighted (accuracy 50%, comprehensiveness 16.7%, precision 16.7%, understandability 16.7%), and Communication-weighted (accuracy 20%, comprehensiveness 30%, precision 25%, understandability 25%). (B) Tier stability across all four languages × prompt conditions and all three weighting schemes. Stacked horizontal bars show the number of conditions × scheme combinations in which each model was classified as Tier 1 (top performers), Tier 2 (mid-tier), or Tier 3 (bottom performers).

Sensitivity analyses using safety-weighted (accuracy: 50% and others: 16.7% each) and communication-weighted (accuracy: 20%, comprehensiveness: 30%, precision: 25%, and understandability: 25%) schemes demonstrated robust tier stability (Multimedia Appendix 17). ChatGPT and Qwen maintained tier 1 classification in all 12 conditions×weight−scheme combinations (100% stability). DeepSeek was classified as tier 1 in 9 (75%) of the 12 combinations, consistently dropping to tier 2 in English no-context conditions under all 3 weighting schemes (rank #7-8), independently confirming the LMM interaction findings. Both ERNIE X1 Turbo and ChatGLM were classified as tier 3 in all 12 combinations (100% stability). This stability at both extremes confirmed that the TOPSIS tier classifications were insensitive to weighting choices for the highest- and lowest-performing models.

### Post Hoc Error-Correction Strategies

The following analyses were conducted exclusively on baseline-incorrect responses (overall baseline error rate: Chinese: 102/1650, 6.18% and English: 78/1650, 4.73%) and were presented separately from the full-benchmark prompt conditions above. Δ-accuracy values represented within-error-subset correction rates (Multimedia Appendix 18; Figure 5).

**Figure 5:**
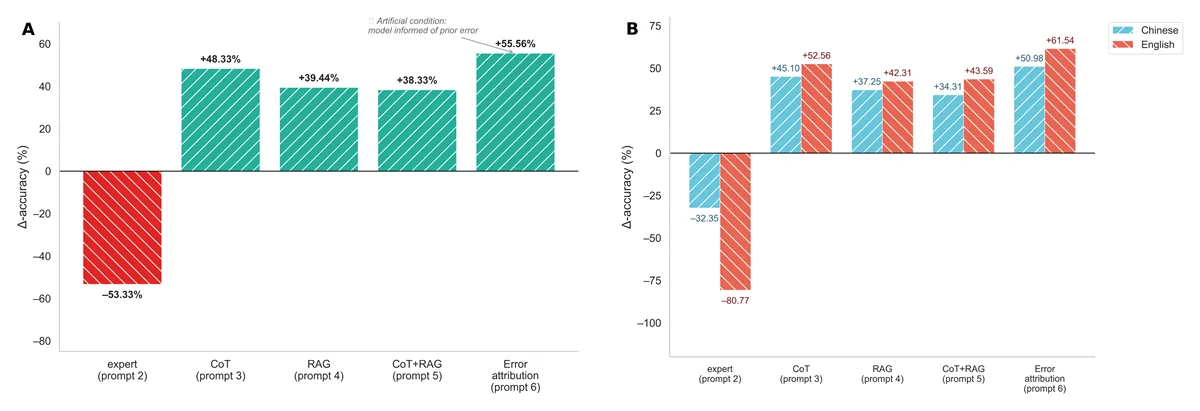
Post hoc accuracy improvement (Δ-Accuracy) following error-correction strategies. (A) Overall improvement pooled across both languages and all 11 models. (B) Comparison of Δ-Accuracy between Chinese (cyan) and English (red) queries. CoT: chain-of-thought; RAG: retrieval-augmented generation.

CoT prompting achieved a correction rate of 48.33% (87/180) overall (Chinese: 46/102, 45.10% and English: 41/78, 52.56%). RAG yielded moderate improvements (71/180, 39.44%), and combined CoT+RAG (69/180, 38.33%) did not produce additive effects. The error-attribution condition achieved the highest correction rate (100/180, 55.56%; Chinese: 52/102, 50.98% and English: 48/78, 61.54%); however, this condition provided models with privileged information that their prior answer was incorrect and should be interpreted as an upper-bound diagnostic test.

The expert prompt was associated with an overall Δ of −53.33% (−96/180, Chinese: −33/102, −32.35% and English: −63/78, −80.77%). These negative Δ values indicated that the expert prompt introduced more new errors than it corrected. When models had very few baseline errors (eg, Grok English: 4 errors), introducing even a small number of new errors produced extremely large negative Δ values (eg, −13/4, −325%).

## Discussion

We evaluated 11 LLMs on 150 binary consumer health questions in both English and Chinese. Aggregate accuracy was high across languages (Chinese: 1548/1650, 93.82% and English: 1572/1650, 95.27%), with a precisely null language main effect. No language main effects for any individual model remained significant after FDR correction, consistent with recent cross-lingual benchmarking showing that question type, rather than language, was the primary determinant of LLM performance on the Chinese National Medical Licensing Examination [17]. Similar conclusions emerged from a multilingual evaluation across German, French, and Italian medical examinations, where performance variability across models exceeded variability across languages [18].

The absence of a systematic language effect on accuracy across 11 models was broadly reassuring. Earlier evaluations consistently reported performance drops when medical LLMs were queried in non-English languages. Jin et al [13] found that English prompts yielded substantially higher accuracy across multiple languages, and a comprehensive platform spanning 67 countries and 27 languages documented persistent, albeit narrowing, English advantages in medical examination performance [19]. Our finding of near-identical English-Chinese accuracy indicated that the cross-linguistic gap had largely closed for this high-resource language pair in binary question answering, though the extent to which this generalizes to open-ended clinical tasks remained unclear.

The concentration of the language interaction in DeepSeek alone pointed to model-level rather than language-level variation. DeepSeek achieved the highest Chinese understandability score (mean: 7.92) among all models tested but dropped to 7.22 in English. Previous work has reported a similar pattern. In an evaluation of prostate cancer radiotherapy questions, DeepSeek received top-quality ratings in 75.8% of Chinese responses vs ChatGPT’s 36.4%, while the relationship shifted in English [20]. In bilingual neuro-oncology consultations involving ChatGPT-4o, DeepSeek-R1, and Doubao, all 3 exceeded 90% diagnostic accuracy across languages, but language-dependent differences emerged in specific clinical tasks [21]. On the 2024 Chinese National Medical Licensing Examination, DeepSeek-R1 achieved 92.0% accuracy compared with GPT-4o’s 87.2% [22]. In a separate evaluation using the 2021 examination, Chinese-developed models consistently outperformed OpenAI models, with ERNIE 4.5 Turbo reaching 95.3% and Qwen 3 reaching 92.5% [23]. Our study extended these observations by showing that DeepSeek’s cross-linguistic limitation was specific to communicative clarity rather than factual accuracy. ChatGPT maintained balanced high performance across both languages, and Qwen demonstrated robust stability.

The expert prompt reduced comprehensiveness and precision but did not affect understandability. This pattern should be interpreted with caution. The expert prompt differed from the no-context prompt in several respects beyond the expert framing itself, including prompt length and forced response structuring. Any of these design features could have contributed to the observed effect. Several models (DeepSeek, Qwen, Kimi, and Grok) showed significantly less degradation than the reference model (ChatGLM), suggesting model-specific resilience to prompt-format effects. These results were specific to this prompt design, binary scoring framework, and benchmark, and should not be taken as evidence that expert-framed prompting generally impaired LLM performance. Among post hoc correction strategies applied to baseline errors, CoT prompting achieved the highest correction rate among realistic strategies. Error attribution achieved a higher rate but should be regarded as an artificial upper bound, as it explicitly informed models of prior errors.

This study has several limitations. First, binary health questions, while enabling objective scoring, did not capture complex clinical reasoning involving probabilistic assessment, treatment trade-offs, uncertainty expression, shared decision-making, or harm avoidance. The high baseline accuracy created ceiling effects that limit performance discrimination. Second, several models had built-in web search enabled during evaluation (DeepSeek R1, Kimi k1.5, Hunyuan T1, ERNIE X1 Turbo, ChatGLM 4, and GPT-4.5), which may have influenced responses through retrieval of language-specific web content. Third, ground-truth labels were used as provided by the TREC Health Misinformation Track without independent revalidation against current evidence. Fourth, results reflect model capabilities at a single time point (May 2025) and may not generalize to subsequent model versions. Fifth, RAG findings were exploratory, as Google Search results were dynamic, nonreproducible, and not the primary search engine used in mainland China. Finally, our English-Chinese comparison could not be generalized to low-resource languages where LLM performance may differ substantially.

Contemporary LLMs achieved high binary accuracy on consumer health questions in both English and Chinese (>93%), with no significant aggregate language effect on either accuracy or communication quality. Model-specific variation in communication quality was concentrated in a single model and single metric: DeepSeek’s English understandability decrement, which was independently confirmed by TOPSIS tier analysis. One 3-way interaction (Grok×English×expert, FDR *P*=.02) indicated model-specific sensitivity to combined cross-linguistic and prompt-format conditions. Cross-linguistic communication quality appeared to be a model-specific rather than universal concern, warranting targeted monitoring in individual model evaluation for multilingual health information deployment.

## Data Availability

All data analyzed in this study are publicly available through the Text Retrieval Conference Health Misinformation Track.

## Appendix

Multimedia Appendix 1 Model specifications and access details for 11 large language models.

Multimedia Appendix 2 Institutional ethics exemption letter.

Multimedia Appendix 3 150 questions (English + Chinese) with ground-truth labels.

Multimedia Appendix 4 Detailed prompts both in Chinese and English.

Multimedia Appendix 5 Full scoring rubric with anchors and examples (1-3/4-6/7-9/10 anchors for each metric).

Multimedia Appendix 6 Representative retrieval-augmented generation search logs.

Multimedia Appendix 7 TRIPOD-LLM Reporting Checklist.

Multimedia Appendix 8 Inter-rater reliability:Cohen κ and weighted κ values.

Multimedia Appendix 9 Accuracy by dataset-year subset for 11 large language models under no-context prompting. Grouped bar charts showing model-level accuracy (%) stratified by Text Retrieval Conference Health Misinformation Track dataset year: 2019, 2021, and 2022. (A) Chinese-language input. (B) English-language input.

Multimedia Appendix 10 Distribution of communication quality scores by language for 11 large language models. Split violin plots showing the distribution of scores under the no-context condition, with Chinese responses (blue, left half) and English responses (red, right half) displayed as mirrored density distributions. Inner box plots display the median and interquartile range. (A) Comprehensiveness. (B) Precision. (C) Understandability. Each violin represents responses across 150 health questions scored on a 1-10 scale by 2 independent bilingual clinical physicians.

Multimedia Appendix 11 Quality metric descriptive statistics by model, language, and prompt condition.

Multimedia Appendix 12 Three-way interaction effects (model×language×prompt) on 4 metrics. Forest plots showing coefficient estimates (dots) with 95% CIs (horizontal lines) for 3-way interaction terms (model×english×expert prompt) from (A) generalized estimating equations for accuracy and (B-D) linear mixed models for comprehensiveness, understandability, and precision, respectively. Each panel displays the 10 model×english×expert prompt interaction terms, with ChatGLM as the reference model, Chinese as the reference language, and no-context as the reference prompt. GEE: generalized estimating equation; LMM: linear mixed model; P2: expert prompt condition.

Multimedia Appendix 13 Full generalized estimating equation coefficients for binary accuracy with false discovery rate correction.

Multimedia Appendix 14 Full linear mixed model coefficients for quality metrics with false discovery rate correction.

Multimedia Appendix 15 Three-way interaction effects (model×language×prompt) across all metrics.

Multimedia Appendix 16 Two-by-two composite of 4 forest plot panels: (A) accuracy from a generalized estimating equation (GEE) with binomial link, (B) comprehensiveness, (C) understandability, and (D) precision from linear mixed-effects models (LMMs).

Multimedia Appendix 17 Technique for order of preference by similarity to ideal solution (TOPSIS) sensitivity analysis: composite scores, tiers, and rankings under 3 weighting schemes.

Multimedia Appendix 18 Post hoc error correction rates (Δ-accuracy) by model and language, with pooled counts.

Multimedia Appendix 19 Detailed prompts and complete conversation logs of Claude Opus 4.6 (Anthropic).

## Abbreviations

CoT: chain-of-thought
FDR: false discovery rate
GEE: generalized estimating equation
LLM: large language model
LMM: linear mixed model
RAG: retrieval-augmented generation
TOPSIS: technique for order of preference by similarity to ideal solution
TREC: Text Retrieval Conference
TRIPOD-LLM: Transparent Reporting of a Multivariable Model for Individual Prognosis or Diagnosis–Large Language Model
