# Supplementary material for: Bilingual Performance of Large Language Models in Answering Consumer Health Questions in English and Chinese: Comparative Benchmark Study
Source: J Med Internet Res. 2026 Sep 29;28:e96587. doi: 10.2196/96587 (PMC13662449; doi:10.2196/96587)
Supplement: Supplementary file 1 [file jmir_v28i1e96587_app1.docx]

| **Multimedia Appendix 1. Model Specifications and Access Details for 11 Large Language Models** | | | | | | |
| --- | --- | --- | --- | --- | --- | --- |
| Name | Version | Platform | Date | Web search on/off | Memory on/off | Fresh session |
| ChatGLM | 4 | Zhipu AI | May,2025 | On | Off | Yes |
| ChatGPT | 4.5 | OpenAI | May,2025 | On | Off | Yes |
| Claude | Sonnet 4 | Anthropic | May,2025 | Off | Off | Yes |
| DeepSeek | R1 | DeepSeek | May,2025 | On | Off | Yes |
| Doubao | NA | ByteDance | May,2025 | Off | Off | Yes |
| ERNIEBot | X1 Turbo | Baidu | May,2025 | On | Off | Yes |
| Gemini | 2.5 flash | Google | May,2025 | Off | Off | Yes |
| Grok | 3 | xAI | May,2025 | Off | Off | Yes |
| Hunyuan | T1 | Tencent | May,2025 | On | Off | Yes |
| Kimi | k1.5 | Moonshot AI | May,2025 | On | Off | Yes |
| Qwen | 3 | Alibaba Group | May,2025 | Off | Off | Yes |
