# Supplementary material for: Bilingual Performance of Large Language Models in Answering Consumer Health Questions in English and Chinese: Comparative Benchmark Study
Source: J Med Internet Res. 2026 Sep 29;28:e96587. doi: 10.2196/96587 (PMC13662449; doi:10.2196/96587)
Supplement: Supplementary file 2 [file jmir_v28i1e96587_app11.docx]

| **Multimedia Appendix11. Quality Metric Descriptive Statistics by Model, Language, and Prompt Condition** | | | | | |
| --- | --- | --- | --- | --- | --- |
| Model | Language | Prompt | Comprehensiveness | Precision | Understandability |
| ChatGLM | Chinese | No-context | 7 (6–7) | 7 (6–7) | 7 (6–7) |
| ChatGLM | Chinese | Expert | 6 (6–7) | 6 (6–6) | 7 (6–7) |
| ChatGLM | English | No-context | 7 (6–7) | 7 (6–7) | 7 (6–7) |
| ChatGLM | English | Expert | 6 (6–7) | 6 (6–6) | 7 (6–7) |
| ChatGPT | Chinese | No-context | 8 (7.5–8) | 8 (8–8) | 8 (8–8) |
| ChatGPT | Chinese | Expert | 8 (8–9) | 9 (9–9) | 9 (8–9) |
| ChatGPT | English | No-context | 8 (8–8) | 8 (8–8) | 8 (8–8) |
| ChatGPT | English | Expert | 8 (8–9) | 9 (9–9) | 9 (8–9) |
| Claude | Chinese | No-context | 8 (7–8) | 8 (7.5–8) | 8 (7.5–8) |
| Claude | Chinese | Expert | 8 (7–8) | 8 (7.5–8) | 8 (7–8) |
| Claude | English | No-context | 7.5 (7–8) | 8 (7.5–8) | 8 (7.5–8) |
| Claude | English | Expert | 8 (7–8) | 8 (7.5–8) | 8 (7–8) |
| DeepSeek | Chinese | No-context | 8 (7.5–8) | 8 (8–8) | 8 (8–9) |
| DeepSeek | Chinese | Expert | 9 (8–9) | 9 (9–9) | 9 (8–9) |
| DeepSeek | English | No-context | 8 (7–8) | 8 (8–8) | 8 (7–8) |
| DeepSeek | English | Expert | 8 (8–8) | 8 (8–8) | 8 (8–8) |
| Doubao | Chinese | No-context | 8 (8–8) | 8 (8–8) | 8 (8–8) |
| Doubao | Chinese | Expert | 8 (8–8) | 9 (8–9) | 8 (7.5–8) |
| Doubao | English | No-context | 8 (8–8) | 8 (8–8) | 8 (8–8) |
| Doubao | English | Expert | 8 (8–8) | 8 (8–9) | 8 (7–8) |
| ERNIEBot | Chinese | No-context | 7 (6–7) | 7 (6–7) | 7 (6.125–7) |
| ERNIEBot | Chinese | Expert | 7 (6–7) | 7 (6–7) | 7 (6–7) |
| ERNIEBot | English | No-context | 7 (6.5–7) | 7 (6–7) | 7 (7–7) |
| ERNIEBot | English | Expert | 7 (6–7) | 7 (6–7) | 7 (6.5–7) |
| Gemini | Chinese | No-context | 8 (7.5–8) | 8 (8–8) | 8 (8–8) |
| Gemini | Chinese | Expert | 8 (7–8) | 8 (8–8) | 8 (8–8) |
| Gemini | English | No-context | 8 (7.5–8) | 8 (8–8) | 8 (8–8) |
| Gemini | English | Expert | 8 (7–8) | 8 (8–8) | 8 (8–8) |
| Grok | Chinese | No-context | 8 (7–8) | 8 (8–8) | 8 (8–8) |
| Grok | Chinese | Expert | 8 (7.5–8) | 8 (8–8) | 8 (8–8) |
| Grok | English | No-context | 8 (7–8) | 8 (8–8) | 8 (8–8) |
| Grok | English | Expert | 8 (7–8) | 8 (8–8) | 8 (8–8) |
| Hunyuan | Chinese | No-context | 8 (7–8) | 8 (7.5–8) | 8 (7–8) |
| Hunyuan | Chinese | Expert | 8 (7–8) | 8 (8–8) | 8 (7–8) |
| Hunyuan | English | No-context | 8 (7–8) | 8 (7.5–8) | 8 (7–8) |
| Hunyuan | English | Expert | 8 (7–8) | 8 (8–8) | 8 (7–8) |
| Kimi | Chinese | No-context | 7 (6–7) | 7 (6.125–7.5) | 7 (6–7) |
| Kimi | Chinese | Expert | 7 (7–8) | 7 (7–8) | 7 (7–8) |
| Kimi | English | No-context | 7 (6–7) | 7 (7–7.5) | 7 (6–7) |
| Kimi | English | Expert | 7 (7–8) | 7 (7–8) | 7 (7–8) |
| Qwen | Chinese | No-context | 8 (8–8) | 8 (8–8) | 8 (8–9) |
| Qwen | Chinese | Expert | 9 (8–9) | 9 (9–9) | 9 (8.5–9) |
| Qwen | English | No-context | 8 (7.625–8) | 8 (8–8) | 8 (7.5–8) |
| Qwen | English | Expert | 8 (8–8) | 8 (8–8) | 8 (8–8) |
