# Supplementary material for: Bilingual Performance of Large Language Models in Answering Consumer Health Questions in English and Chinese: Comparative Benchmark Study
Source: J Med Internet Res. 2026 Sep 29;28:e96587. doi: 10.2196/96587 (PMC13662449; doi:10.2196/96587)
Supplement: Supplementary file 3 [file jmir_v28i1e96587_app13.docx]

| **Multimedia Appendix 13. Full Generalized Estimating Equation Coefficients for Binary Accuracy With False Discovery Rate Correction** | | | | | | | |
| --- | --- | --- | --- | --- | --- | --- | --- |
| Term | Category | beta | SE | 95% CI | z | Raw P | FDR P |
| Intercept | Intercept | 2.44 | 0.30 | [1.85, 3.03] | 8.12 | <1.00E-03 | <1.00E-03 |
| ChatGPT | Model main | 0.74 | 0.39 | [-0.03, 1.50] | 1.88 | 0.06 | 0.37 |
| Claude | Model main | 0.43 | 0.27 | [-0.09, 0.95] | 1.64 | 0.10 | 0.37 |
| DeepSeek | Model main | 0.74 | 0.35 | [0.05, 1.42] | 2.11 | 0.04 | 0.37 |
| Doubao | Model main | 0.57 | 0.35 | [-0.10, 1.25] | 1.66 | 0.10 | 0.37 |
| ERNIEBot | Model main | 0.20 | 0.31 | [-0.41, 0.81] | 0.63 | 0.53 | 0.81 |
| Gemini | Model main | 0.09 | 0.28 | [-0.46, 0.65] | 0.33 | 0.74 | 0.96 |
| Grok | Model main | 0.09 | 0.31 | [-0.52, 0.71] | 0.30 | 0.76 | 0.96 |
| Hunyuan | Model main | 0.43 | 0.38 | [-0.30, 1.17] | 1.15 | 0.25 | 0.59 |
| Kimi | Model main | -0.25 | 0.29 | [-0.82, 0.33] | -0.83 | 0.41 | 0.78 |
| Qwen | Model main | 0.43 | 0.38 | [-0.30, 1.17] | 1.15 | 0.25 | 0.59 |
| English | Language main | 0.00 | — | — | — | NA | NA |
| Expert | Prompt main | -0.39 | 0.28 | [-0.93, 0.16] | -1.39 | 0.17 | 0.47 |
| ChatGPT x English | Model x Language | 0.00 | 0.55 | [-1.08, 1.08] | 0.00 | 1.00 | 1.00 |
| Claude x English | Model x Language | 0.30 | 0.43 | [-0.54, 1.14] | 0.71 | 0.48 | 0.79 |
| DeepSeek x English | Model x Language | -0.54 | 0.38 | [-1.29, 0.21] | -1.41 | 0.16 | 0.47 |
| Doubao x English | Model x Language | 0.00 | 0.47 | [-0.93, 0.93] | 0.00 | 1.00 | 1.00 |
| ERNIEBot x English | Model x Language | 0.38 | 0.38 | [-0.36, 1.12] | 1.00 | 0.32 | 0.69 |
| Gemini x English | Model x Language | 0.64 | 0.39 | [-0.12, 1.40] | 1.65 | 0.10 | 0.37 |
| Grok x English | Model x Language | 1.06 | 0.41 | [0.26, 1.86] | 2.59 | 0.01 | 0.14 |
| Hunyuan x English | Model x Language | 0.49 | 0.37 | [-0.23, 1.21] | 1.34 | 0.18 | 0.49 |
| Kimi x English | Model x Language | 0.55 | 0.32 | [-0.07, 1.18] | 1.73 | 0.08 | 0.37 |
| Qwen x English | Model x Language | 0.30 | 0.37 | [-0.42, 1.03] | 0.81 | 0.41 | 0.78 |
| ChatGPT x Expert | Model x Prompt | -0.15 | 0.38 | [-0.90, 0.59] | -0.40 | 0.69 | 0.95 |
| Claude x Expert | Model x Prompt | -0.05 | 0.29 | [-0.61, 0.51] | -0.17 | 0.86 | 1.00 |
| DeepSeek x Expert | Model x Prompt | -0.04 | 0.47 | [-0.95, 0.87] | -0.09 | 0.93 | 1.00 |
| Doubao x Expert | Model x Prompt | -0.28 | 0.35 | [-0.96, 0.41] | -0.79 | 0.43 | 0.78 |
| ERNIEBot x Expert | Model x Prompt | -0.44 | 0.39 | [-1.20, 0.32] | -1.13 | 0.26 | 0.59 |
| Gemini x Expert | Model x Prompt | 0.20 | 0.38 | [-0.54, 0.94] | 0.54 | 0.59 | 0.87 |
| Grok x Expert | Model x Prompt | 0.72 | 0.39 | [-0.05, 1.50] | 1.84 | 0.07 | 0.37 |
| Hunyuan x Expert | Model x Prompt | 0.69 | 0.46 | [-0.21, 1.58] | 1.51 | 0.13 | 0.44 |
| Kimi x Expert | Model x Prompt | 0.31 | 0.41 | [-0.48, 1.11] | 0.77 | 0.44 | 0.78 |
| Qwen x Expert | Model x Prompt | 0.15 | 0.44 | [-0.71, 1.00] | 0.34 | 0.73 | 0.96 |
| English x Expert | Language x Prompt | 0.00 | 0.27 | [-0.52, 0.52] | 0.00 | 1.00 | 1.00 |
| ChatGPT x English x Expert | Model x Language x Prompt | -0.37 | 0.49 | [-1.32, 0.59] | -0.75 | 0.45 | 0.78 |
| Claude x English x Expert | Model x Language x Prompt | 0.01 | 0.60 | [-1.16, 1.18] | 0.01 | 0.99 | 1.00 |
| DeepSeek x English x Expert | Model x Language x Prompt | 0.32 | 0.47 | [-0.60, 1.25] | 0.68 | 0.49 | 0.79 |
| Doubao x English x Expert | Model x Language x Prompt | 0.00 | 0.54 | [-1.05, 1.05] | 0.00 | 1.00 | 1.00 |
| ERNIEBot x English x Expert | Model x Language x Prompt | -0.07 | 0.45 | [-0.96, 0.82] | -0.15 | 0.88 | 1.00 |
| Gemini x English x Expert | Model x Language x Prompt | -0.87 | 0.52 | [-1.88, 0.14] | -1.69 | 0.09 | 0.37 |
| Grok x English x Expert | Model x Language x Prompt | -1.88 | 0.54 | [-2.94, -0.82] | -3.48 | 0.00 | 0.02 |
| Hunyuan x English x Expert | Model x Language x Prompt | -1.03 | 0.52 | [-2.05, -0.01] | -1.98 | 0.05 | 0.37 |
| Kimi x English x Expert | Model x Language x Prompt | 0.07 | 0.43 | [-0.77, 0.91] | 0.17 | 0.87 | 1.00 |
| Qwen x English x Expert | Model x Language x Prompt | -0.19 | 0.45 | [-1.07, 0.69] | -0.42 | 0.67 | 0.95 |
