# Supplementary material for: Bilingual Performance of Large Language Models in Answering Consumer Health Questions in English and Chinese: Comparative Benchmark Study
Source: J Med Internet Res. 2026 Sep 29;28:e96587. doi: 10.2196/96587 (PMC13662449; doi:10.2196/96587)
Supplement: Supplementary file 4 [file jmir_v28i1e96587_app14.docx]

| **Multimedia Appendix 14. Full Linear Mixed Model Coefficients for Quality Metrics With False Discovery Rate Correction** | | | | | | | | | |
| --- | --- | --- | --- | --- | --- | --- | --- | --- | --- |
| Metric | Term | Category | beta | SE | 95% CI | t | Raw P | FDR P | Global FDR P |
| Comprehensiveness | ChatGPT | Model main | 1.46 | 0.17 | [1.12, 1.79] | 8.52 | <1.00E-03 | <1.00E-03 | NA |
| Comprehensiveness | Claude | Model main | 0.98 | 0.17 | [0.64, 1.31] | 5.71 | <1.00E-03 | <1.00E-03 | NA |
| Comprehensiveness | DeepSeek | Model main | 1.37 | 0.17 | [1.03, 1.70] | 8 | <1.00E-03 | <1.00E-03 | NA |
| Comprehensiveness | Doubao | Model main | 1.39 | 0.17 | [1.05, 1.73] | 8.13 | <1.00E-03 | <1.00E-03 | NA |
| Comprehensiveness | ERNIEBot | Model main | 0.17 | 0.17 | [-0.16, 0.51] | 1.01 | 0.31 | 0.51 | NA |
| Comprehensiveness | Gemini | Model main | 1.07 | 0.17 | [0.73, 1.40] | 6.24 | <1.00E-03 | <1.00E-03 | NA |
| Comprehensiveness | Grok | Model main | 0.89 | 0.17 | [0.55, 1.22] | 5.19 | <1.00E-03 | <1.00E-03 | NA |
| Comprehensiveness | Hunyuan | Model main | 1.05 | 0.17 | [0.71, 1.38] | 6.12 | <1.00E-03 | <1.00E-03 | NA |
| Comprehensiveness | Kimi | Model main | -0.02 | 0.17 | [-0.35, 0.32] | -0.1 | 0.92 | 0.97 | NA |
| Comprehensiveness | Qwen | Model main | 1.35 | 0.17 | [1.01, 1.69] | 7.9 | <1.00E-03 | <1.00E-03 | NA |
| Comprehensiveness | English | Language main | 0.01 | 0.17 | [-0.32, 0.35] | 0.08 | 0.94 | 0.97 | NA |
| Comprehensiveness | Expert | Prompt main | -0.56 | 0.17 | [-0.89, -0.22] | -3.26 | 1.00E-03 | 3.31E-03 | NA |
| Comprehensiveness | ChatGPT x English | Model x Language | 0.01 | 0.24 | [-0.46, 0.49] | 0.06 | 0.96 | 0.97 | 0.97 |
| Comprehensiveness | Claude x English | Model x Language | -0.01 | 0.24 | [-0.48, 0.46] | -0.04 | 0.97 | 0.97 | 0.97 |
| Comprehensiveness | DeepSeek x English | Model x Language | -0.28 | 0.24 | [-0.75, 0.19] | -1.16 | 0.25 | 0.46 | 0.56 |
| Comprehensiveness | Doubao x English | Model x Language | -0.02 | 0.24 | [-0.49, 0.45] | -0.08 | 0.93 | 0.97 | 0.97 |
| Comprehensiveness | ERNIEBot x English | Model x Language | 0.15 | 0.24 | [-0.32, 0.63] | 0.63 | 0.53 | 0.74 | 0.85 |
| Comprehensiveness | Gemini x English | Model x Language | 0.26 | 0.24 | [-0.22, 0.73] | 1.06 | 0.29 | 0.5 | 0.58 |
| Comprehensiveness | Grok x English | Model x Language | 0.34 | 0.24 | [-0.13, 0.81] | 1.41 | 0.16 | 0.36 | 0.46 |
| Comprehensiveness | Hunyuan x English | Model x Language | 0.14 | 0.24 | [-0.34, 0.61] | 0.56 | 0.57 | 0.77 | 0.86 |
| Comprehensiveness | Kimi x English | Model x Language | 0.28 | 0.24 | [-0.19, 0.75] | 1.16 | 0.25 | 0.46 | 0.56 |
| Comprehensiveness | Qwen x English | Model x Language | 0.03 | 0.24 | [-0.45, 0.50] | 0.11 | 0.91 | 0.97 | 0.97 |
| Comprehensiveness | ChatGPT x Expert | Model x Prompt | 0.74 | 0.24 | [0.27, 1.22] | 3.08 | 2.00E-03 | 6.14E-03 | 1.43E-02 |
| Comprehensiveness | Claude x Expert | Model x Prompt | 0.35 | 0.24 | [-0.13, 0.82] | 1.43 | 0.15 | 0.36 | 0.45 |
| Comprehensiveness | DeepSeek x Expert | Model x Prompt | 1.23 | 0.24 | [0.76, 1.70] | 5.09 | <1.00E-03 | <1.00E-03 | <1.00E-03 |
| Comprehensiveness | Doubao x Expert | Model x Prompt | 0.3 | 0.24 | [-0.18, 0.77] | 1.23 | 0.22 | 0.45 | 0.55 |
| Comprehensiveness | ERNIEBot x Expert | Model x Prompt | 0.2 | 0.24 | [-0.27, 0.68] | 0.84 | 0.4 | 0.59 | 0.66 |
| Comprehensiveness | Gemini x Expert | Model x Prompt | 0.44 | 0.24 | [-0.03, 0.91] | 1.82 | 0.07 | 0.17 | 0.26 |
| Comprehensiveness | Grok x Expert | Model x Prompt | 0.89 | 0.24 | [0.41, 1.36] | 3.67 | <1.00E-03 | <1.00E-03 | <1.00E-03 |
| Comprehensiveness | Hunyuan x Expert | Model x Prompt | 0.71 | 0.24 | [0.23, 1.18] | 2.92 | 3.00E-03 | 8.60E-03 | 1.64E-02 |
| Comprehensiveness | Kimi x Expert | Model x Prompt | 1.06 | 0.24 | [0.58, 1.53] | 4.37 | <1.00E-03 | <1.00E-03 | <1.00E-03 |
| Comprehensiveness | Qwen x Expert | Model x Prompt | 1.16 | 0.24 | [0.69, 1.63] | 4.8 | <1.00E-03 | <1.00E-03 | <1.00E-03 |
| Comprehensiveness | English x Expert | Language x Prompt | -0.04 | 0.24 | [-0.51, 0.43] | -0.17 | 0.87 | 0.97 | 0.97 |
| Comprehensiveness | ChatGPT x English x Expert | Model x Language x Prompt | -0.21 | 0.34 | [-0.88, 0.46] | -0.62 | 0.53 | 0.74 | 0.85 |
| Comprehensiveness | Claude x English x Expert | Model x Language x Prompt | 0.14 | 0.34 | [-0.53, 0.81] | 0.41 | 0.68 | 0.89 | 0.94 |
| Comprehensiveness | DeepSeek x English x Expert | Model x Language x Prompt | -0.32 | 0.34 | [-0.99, 0.35] | -0.94 | 0.35 | 0.56 | 0.62 |
| Comprehensiveness | Doubao x English x Expert | Model x Language x Prompt | 0.03 | 0.34 | [-0.64, 0.70] | 0.08 | 0.94 | 0.97 | 0.97 |
| Comprehensiveness | ERNIEBot x English x Expert | Model x Language x Prompt | 0.09 | 0.34 | [-0.58, 0.76] | 0.26 | 0.79 | 0.97 | 0.97 |
| Comprehensiveness | Gemini x English x Expert | Model x Language x Prompt | -0.39 | 0.34 | [-1.06, 0.28] | -1.13 | 0.26 | 0.46 | 0.56 |
| Comprehensiveness | Grok x English x Expert | Model x Language x Prompt | -0.76 | 0.34 | [-1.43, -0.09] | -2.22 | 2.60E-02 | 0.07 | 0.11 |
| Comprehensiveness | Hunyuan x English x Expert | Model x Language x Prompt | -0.31 | 0.34 | [-0.98, 0.36] | -0.9 | 0.37 | 0.57 | 0.64 |
| Comprehensiveness | Kimi x English x Expert | Model x Language x Prompt | 0.1 | 0.34 | [-0.57, 0.77] | 0.28 | 0.78 | 0.97 | 0.97 |
| Comprehensiveness | Qwen x English x Expert | Model x Language x Prompt | -0.43 | 0.34 | [-1.10, 0.24] | -1.27 | 0.2 | 0.44 | 0.54 |
| Precision | ChatGPT | Model main | 1.73 | 0.17 | [1.39, 2.07] | 9.96 | <1.00E-03 | <1.00E-03 | NA |
| Precision | Claude | Model main | 1.29 | 0.17 | [0.95, 1.63] | 7.41 | <1.00E-03 | <1.00E-03 | NA |
| Precision | DeepSeek | Model main | 1.53 | 0.17 | [1.19, 1.87] | 8.83 | <1.00E-03 | <1.00E-03 | NA |
| Precision | Doubao | Model main | 1.42 | 0.17 | [1.08, 1.76] | 8.2 | <1.00E-03 | <1.00E-03 | NA |
| Precision | ERNIEBot | Model main | 0.18 | 0.17 | [-0.16, 0.52] | 1.06 | 0.29 | 0.46 | NA |
| Precision | Gemini | Model main | 1.3 | 0.17 | [0.96, 1.64] | 7.49 | <1.00E-03 | <1.00E-03 | NA |
| Precision | Grok | Model main | 1.23 | 0.17 | [0.89, 1.57] | 7.07 | <1.00E-03 | <1.00E-03 | NA |
| Precision | Hunyuan | Model main | 1.24 | 0.17 | [0.90, 1.58] | 7.12 | <1.00E-03 | <1.00E-03 | NA |
| Precision | Kimi | Model main | 0.22 | 0.17 | [-0.12, 0.56] | 1.29 | 0.2 | 0.38 | NA |
| Precision | Qwen | Model main | 1.48 | 0.17 | [1.14, 1.82] | 8.54 | <1.00E-03 | <1.00E-03 | NA |
| Precision | English | Language main | 0.01 | 0.17 | [-0.33, 0.35] | 0.08 | 0.94 | 0.97 | NA |
| Precision | Expert | Prompt main | -0.66 | 0.17 | [-1.00, -0.32] | -3.78 | <1.00E-03 | <1.00E-03 | NA |
| Precision | ChatGPT x English | Model x Language | -0.01 | 0.25 | [-0.49, 0.47] | -0.04 | 0.97 | 0.97 | 0.97 |
| Precision | Claude x English | Model x Language | -0.05 | 0.25 | [-0.53, 0.43] | -0.2 | 0.84 | 0.95 | 0.97 |
| Precision | DeepSeek x English | Model x Language | -0.23 | 0.25 | [-0.71, 0.25] | -0.94 | 0.35 | 0.5 | 0.62 |
| Precision | Doubao x English | Model x Language | -0.02 | 0.25 | [-0.50, 0.47] | -0.07 | 0.95 | 0.97 | 0.97 |
| Precision | ERNIEBot x English | Model x Language | 0.13 | 0.25 | [-0.35, 0.61] | 0.52 | 0.61 | 0.77 | 0.88 |
| Precision | Gemini x English | Model x Language | 0.25 | 0.25 | [-0.23, 0.73] | 1.03 | 0.3 | 0.46 | 0.59 |
| Precision | Grok x English | Model x Language | 0.31 | 0.25 | [-0.17, 0.79] | 1.25 | 0.21 | 0.38 | 0.55 |
| Precision | Hunyuan x English | Model x Language | 0.14 | 0.25 | [-0.34, 0.62] | 0.57 | 0.57 | 0.76 | 0.86 |
| Precision | Kimi x English | Model x Language | 0.3 | 0.25 | [-0.18, 0.78] | 1.21 | 0.23 | 0.39 | 0.56 |
| Precision | Qwen x English | Model x Language | 0.05 | 0.25 | [-0.43, 0.53] | 0.2 | 0.84 | 0.95 | 0.97 |
| Precision | ChatGPT x Expert | Model x Prompt | 1.04 | 0.25 | [0.56, 1.52] | 4.25 | <1.00E-03 | <1.00E-03 | <1.00E-03 |
| Precision | Claude x Expert | Model x Prompt | 0.41 | 0.25 | [-0.07, 0.90] | 1.68 | 0.09 | 0.21 | 0.33 |
| Precision | DeepSeek x Expert | Model x Prompt | 1.37 | 0.25 | [0.89, 1.85] | 5.57 | <1.00E-03 | <1.00E-03 | <1.00E-03 |
| Precision | Doubao x Expert | Model x Prompt | 1.04 | 0.25 | [0.56, 1.52] | 4.25 | <1.00E-03 | <1.00E-03 | <1.00E-03 |
| Precision | ERNIEBot x Expert | Model x Prompt | 0.31 | 0.25 | [-0.17, 0.80] | 1.28 | 0.2 | 0.38 | 0.54 |
| Precision | Gemini x Expert | Model x Prompt | 0.76 | 0.25 | [0.28, 1.25] | 3.11 | 2.00E-03 | 5.06E-03 | 1.43E-02 |
| Precision | Grok x Expert | Model x Prompt | 1.07 | 0.25 | [0.59, 1.55] | 4.37 | <1.00E-03 | <1.00E-03 | <1.00E-03 |
| Precision | Hunyuan x Expert | Model x Prompt | 0.9 | 0.25 | [0.42, 1.39] | 3.68 | <1.00E-03 | <1.00E-03 | <1.00E-03 |
| Precision | Kimi x Expert | Model x Prompt | 1.13 | 0.25 | [0.65, 1.61] | 4.59 | <1.00E-03 | <1.00E-03 | <1.00E-03 |
| Precision | Qwen x Expert | Model x Prompt | 1.3 | 0.25 | [0.81, 1.78] | 5.28 | <1.00E-03 | <1.00E-03 | <1.00E-03 |
| Precision | English x Expert | Language x Prompt | -0.04 | 0.25 | [-0.52, 0.45] | -0.15 | 0.88 | 0.95 | 0.97 |
| Precision | ChatGPT x English x Expert | Model x Language x Prompt | -0.19 | 0.35 | [-0.87, 0.49] | -0.55 | 0.58 | 0.76 | 0.86 |
| Precision | Claude x English x Expert | Model x Language x Prompt | 0.17 | 0.35 | [-0.51, 0.85] | 0.49 | 0.62 | 0.77 | 0.89 |
| Precision | DeepSeek x English x Expert | Model x Language x Prompt | -0.58 | 0.35 | [-1.26, 0.10] | -1.66 | 0.1 | 0.21 | 0.33 |
| Precision | Doubao x English x Expert | Model x Language x Prompt | -0.32 | 0.35 | [-1.00, 0.36] | -0.92 | 0.36 | 0.5 | 0.63 |
| Precision | ERNIEBot x English x Expert | Model x Language x Prompt | 0.08 | 0.35 | [-0.60, 0.76] | 0.24 | 0.81 | 0.95 | 0.97 |
| Precision | Gemini x English x Expert | Model x Language x Prompt | -0.41 | 0.35 | [-1.09, 0.27] | -1.18 | 0.24 | 0.39 | 0.56 |
| Precision | Grok x English x Expert | Model x Language x Prompt | -0.78 | 0.35 | [-1.46, -0.10] | -2.25 | 2.50E-02 | 0.06 | 0.11 |
| Precision | Hunyuan x English x Expert | Model x Language x Prompt | -0.33 | 0.35 | [-1.01, 0.35] | -0.95 | 0.34 | 0.5 | 0.62 |
| Precision | Kimi x English x Expert | Model x Language x Prompt | 0.05 | 0.35 | [-0.63, 0.73] | 0.15 | 0.88 | 0.95 | 0.97 |
| Precision | Qwen x English x Expert | Model x Language x Prompt | -0.56 | 0.35 | [-1.24, 0.12] | -1.62 | 0.1 | 0.21 | 0.34 |
| Understandability | ChatGPT | Model main | 1.6 | 0.17 | [1.26, 1.94] | 9.2 | <1.00E-03 | <1.00E-03 | NA |
| Understandability | Claude | Model main | 1.16 | 0.17 | [0.82, 1.50] | 6.67 | <1.00E-03 | <1.00E-03 | NA |
| Understandability | DeepSeek | Model main | 1.74 | 0.17 | [1.40, 2.08] | 10.03 | <1.00E-03 | <1.00E-03 | NA |
| Understandability | Doubao | Model main | 1.55 | 0.17 | [1.21, 1.89] | 8.93 | <1.00E-03 | <1.00E-03 | NA |
| Understandability | ERNIEBot | Model main | 0.18 | 0.17 | [-0.16, 0.52] | 1.02 | 0.31 | 0.53 | NA |
| Understandability | Gemini | Model main | 1.18 | 0.17 | [0.84, 1.52] | 6.8 | <1.00E-03 | <1.00E-03 | NA |
| Understandability | Grok | Model main | 1.08 | 0.17 | [0.74, 1.42] | 6.24 | <1.00E-03 | <1.00E-03 | NA |
| Understandability | Hunyuan | Model main | 0.99 | 0.17 | [0.65, 1.33] | 5.72 | <1.00E-03 | <1.00E-03 | NA |
| Understandability | Kimi | Model main | -0.03 | 0.17 | [-0.37, 0.31] | -0.19 | 0.85 | 0.96 | NA |
| Understandability | Qwen | Model main | 1.74 | 0.17 | [1.40, 2.08] | 10.01 | <1.00E-03 | <1.00E-03 | NA |
| Understandability | English | Language main | 0.02 | 0.17 | [-0.32, 0.36] | 0.12 | 0.91 | 0.96 | NA |
| Understandability | Expert | Prompt main | -0.3 | 0.17 | [-0.64, 0.04] | -1.71 | 0.09 | 0.22 | NA |
| Understandability | ChatGPT x English | Model x Language | -0.02 | 0.24 | [-0.50, 0.46] | -0.08 | 0.94 | 0.96 | 0.97 |
| Understandability | Claude x English | Model x Language | -0.01 | 0.24 | [-0.49, 0.47] | -0.04 | 0.97 | 0.97 | 0.97 |
| Understandability | DeepSeek x English | Model x Language | -0.73 | 0.24 | [-1.21, -0.25] | -2.96 | 3.00E-03 | 1.17E-02 | 1.64E-02 |
| Understandability | Doubao x English | Model x Language | -0.21 | 0.24 | [-0.69, 0.27] | -0.86 | 0.39 | 0.62 | 0.66 |
| Understandability | ERNIEBot x English | Model x Language | 0.15 | 0.24 | [-0.33, 0.63] | 0.61 | 0.54 | 0.83 | 0.85 |
| Understandability | Gemini x English | Model x Language | 0.26 | 0.24 | [-0.22, 0.74] | 1.05 | 0.3 | 0.53 | 0.59 |
| Understandability | Grok x English | Model x Language | 0.33 | 0.24 | [-0.15, 0.81] | 1.34 | 0.18 | 0.38 | 0.5 |
| Understandability | Hunyuan x English | Model x Language | 0.14 | 0.24 | [-0.34, 0.62] | 0.58 | 0.56 | 0.83 | 0.86 |
| Understandability | Kimi x English | Model x Language | 0.27 | 0.24 | [-0.21, 0.75] | 1.1 | 0.27 | 0.51 | 0.57 |
| Understandability | Qwen x English | Model x Language | -0.4 | 0.24 | [-0.88, 0.08] | -1.62 | 0.11 | 0.25 | 0.34 |
| Understandability | ChatGPT x Expert | Model x Prompt | 0.72 | 0.24 | [0.24, 1.20] | 2.93 | 3.00E-03 | 1.17E-02 | 1.64E-02 |
| Understandability | Claude x Expert | Model x Prompt | 0.03 | 0.24 | [-0.45, 0.51] | 0.14 | 0.89 | 0.96 | 0.97 |
| Understandability | DeepSeek x Expert | Model x Prompt | 0.6 | 0.24 | [0.12, 1.08] | 2.43 | 1.50E-02 | 4.61E-02 | 0.07 |
| Understandability | Doubao x Expert | Model x Prompt | -0.1 | 0.24 | [-0.58, 0.38] | -0.39 | 0.69 | 0.93 | 0.94 |
| Understandability | ERNIEBot x Expert | Model x Prompt | -0.09 | 0.24 | [-0.57, 0.39] | -0.35 | 0.72 | 0.94 | 0.96 |
| Understandability | Gemini x Expert | Model x Prompt | 0.35 | 0.24 | [-0.13, 0.83] | 1.44 | 0.15 | 0.34 | 0.45 |
| Understandability | Grok x Expert | Model x Prompt | 0.69 | 0.24 | [0.21, 1.17] | 2.8 | 5.00E-03 | 1.79E-02 | 2.58E-02 |
| Understandability | Hunyuan x Expert | Model x Prompt | 0.5 | 0.24 | [0.02, 0.98] | 2.04 | 4.20E-02 | 0.11 | 0.16 |
| Understandability | Kimi x Expert | Model x Prompt | 0.74 | 0.24 | [0.26, 1.22] | 3.02 | 3.00E-03 | 1.17E-02 | 1.64E-02 |
| Understandability | Qwen x Expert | Model x Prompt | 0.6 | 0.24 | [0.12, 1.08] | 2.44 | 1.40E-02 | 4.61E-02 | 0.07 |
| Understandability | English x Expert | Language x Prompt | -0.03 | 0.24 | [-0.51, 0.45] | -0.12 | 0.9 | 0.96 | 0.97 |
| Understandability | ChatGPT x English x Expert | Model x Language x Prompt | -0.38 | 0.35 | [-1.06, 0.30] | -1.09 | 0.27 | 0.51 | 0.57 |
| Understandability | Claude x English x Expert | Model x Language x Prompt | 0.15 | 0.35 | [-0.53, 0.83] | 0.44 | 0.66 | 0.93 | 0.93 |
| Understandability | DeepSeek x English x Expert | Model x Language x Prompt | 0.04 | 0.35 | [-0.64, 0.72] | 0.11 | 0.92 | 0.96 | 0.97 |
| Understandability | Doubao x English x Expert | Model x Language x Prompt | -0.09 | 0.35 | [-0.77, 0.59] | -0.26 | 0.8 | 0.95 | 0.97 |
| Understandability | ERNIEBot x English x Expert | Model x Language x Prompt | 0.09 | 0.35 | [-0.59, 0.77] | 0.26 | 0.8 | 0.95 | 0.97 |
| Understandability | Gemini x English x Expert | Model x Language x Prompt | -0.4 | 0.35 | [-1.08, 0.28] | -1.14 | 0.25 | 0.51 | 0.56 |
| Understandability | Grok x English x Expert | Model x Language x Prompt | -0.79 | 0.35 | [-1.47, -0.11] | -2.29 | 2.20E-02 | 0.06 | 0.1 |
| Understandability | Hunyuan x English x Expert | Model x Language x Prompt | -0.34 | 0.35 | [-1.02, 0.34] | -0.98 | 0.33 | 0.54 | 0.62 |
| Understandability | Kimi x English x Expert | Model x Language x Prompt | 0.09 | 0.35 | [-0.59, 0.77] | 0.26 | 0.8 | 0.95 | 0.97 |
| Understandability | Qwen x English x Expert | Model x Language x Prompt | -0.14 | 0.35 | [-0.82, 0.54] | -0.39 | 0.69 | 0.93 | 0.94 |
