# Supplementary material for: Bilingual Performance of Large Language Models in Answering Consumer Health Questions in English and Chinese: Comparative Benchmark Study
Source: J Med Internet Res. 2026 Sep 29;28:e96587. doi: 10.2196/96587 (PMC13662449; doi:10.2196/96587)
Supplement: Supplementary file 5 [file jmir_v28i1e96587_app15.docx]

| **Multimedia Appendix 15. Three-Way Interaction Effects (Model×Language×Prompt) Across All Metrics** | | | | | | | |
| --- | --- | --- | --- | --- | --- | --- | --- |
| Outcome | Estimator | Model (3-way term) | beta | 95% CI | z/t | Raw P | FDR P |
| Accuracy | GEE | ChatGPT x English x Expert | -0.37 | [-1.32, 0.59] | -0.75 | 0.45 | 0.78 |
| Accuracy | GEE | Claude x English x Expert | 0.01 | [-1.16, 1.18] | 0.01 | 0.99 | 1 |
| Accuracy | GEE | DeepSeek x English x Expert | 0.32 | [-0.60, 1.25] | 0.68 | 0.49 | 0.79 |
| Accuracy | GEE | Doubao x English x Expert | 0 | [-1.05, 1.05] | 0 | 1 | 1 |
| Accuracy | GEE | ERNIEBot x English x Expert | -0.07 | [-0.96, 0.82] | -0.15 | 0.88 | 1 |
| Accuracy | GEE | Gemini x English x Expert | -0.87 | [-1.88, 0.14] | -1.69 | 0.09 | 0.37 |
| Accuracy | GEE | Grok x English x Expert | -1.88 | [-2.94, -0.82] | -3.48 | 1.00E-03 | 2.15E-02 |
| Accuracy | GEE | Hunyuan x English x Expert | -1.03 | [-2.05, -0.01] | -1.98 | 4.80E-02 | 0.37 |
| Accuracy | GEE | Kimi x English x Expert | 0.07 | [-0.77, 0.91] | 0.17 | 0.87 | 1 |
| Accuracy | GEE | Qwen x English x Expert | -0.19 | [-1.07, 0.69] | -0.42 | 0.67 | 0.95 |
| Comprehensiveness | LMM | ChatGPT x English x Expert | -0.21 | [-0.88, 0.46] | -0.62 | 0.53 | 0.74 |
| Comprehensiveness | LMM | Claude x English x Expert | 0.14 | [-0.53, 0.81] | 0.41 | 0.68 | 0.89 |
| Comprehensiveness | LMM | DeepSeek x English x Expert | -0.32 | [-0.99, 0.35] | -0.94 | 0.35 | 0.56 |
| Comprehensiveness | LMM | Doubao x English x Expert | 0.03 | [-0.64, 0.70] | 0.08 | 0.94 | 0.97 |
| Comprehensiveness | LMM | ERNIEBot x English x Expert | 0.09 | [-0.58, 0.76] | 0.26 | 0.79 | 0.97 |
| Comprehensiveness | LMM | Gemini x English x Expert | -0.39 | [-1.06, 0.28] | -1.13 | 0.26 | 0.46 |
| Comprehensiveness | LMM | Grok x English x Expert | -0.76 | [-1.43, -0.09] | -2.22 | 2.60E-02 | 0.07 |
| Comprehensiveness | LMM | Hunyuan x English x Expert | -0.31 | [-0.98, 0.36] | -0.9 | 0.37 | 0.57 |
| Comprehensiveness | LMM | Kimi x English x Expert | 0.1 | [-0.57, 0.77] | 0.28 | 0.78 | 0.97 |
| Comprehensiveness | LMM | Qwen x English x Expert | -0.43 | [-1.10, 0.24] | -1.27 | 0.2 | 0.44 |
| Precision | LMM | ChatGPT x English x Expert | -0.19 | [-0.87, 0.49] | -0.55 | 0.58 | 0.76 |
| Precision | LMM | Claude x English x Expert | 0.17 | [-0.51, 0.85] | 0.49 | 0.62 | 0.77 |
| Precision | LMM | DeepSeek x English x Expert | -0.58 | [-1.26, 0.10] | -1.66 | 0.1 | 0.21 |
| Precision | LMM | Doubao x English x Expert | -0.32 | [-1.00, 0.36] | -0.92 | 0.36 | 0.5 |
| Precision | LMM | ERNIEBot x English x Expert | 0.08 | [-0.60, 0.76] | 0.24 | 0.81 | 0.95 |
| Precision | LMM | Gemini x English x Expert | -0.41 | [-1.09, 0.27] | -1.18 | 0.24 | 0.39 |
| Precision | LMM | Grok x English x Expert | -0.78 | [-1.46, -0.10] | -2.25 | 2.50E-02 | 0.06 |
| Precision | LMM | Hunyuan x English x Expert | -0.33 | [-1.01, 0.35] | -0.95 | 0.34 | 0.5 |
| Precision | LMM | Kimi x English x Expert | 0.05 | [-0.63, 0.73] | 0.15 | 0.88 | 0.95 |
| Precision | LMM | Qwen x English x Expert | -0.56 | [-1.24, 0.12] | -1.62 | 0.1 | 0.21 |
| Understandability | LMM | ChatGPT x English x Expert | -0.38 | [-1.06, 0.30] | -1.09 | 0.27 | 0.51 |
| Understandability | LMM | Claude x English x Expert | 0.15 | [-0.53, 0.83] | 0.44 | 0.66 | 0.93 |
| Understandability | LMM | DeepSeek x English x Expert | 0.04 | [-0.64, 0.72] | 0.11 | 0.92 | 0.96 |
| Understandability | LMM | Doubao x English x Expert | -0.09 | [-0.77, 0.59] | -0.26 | 0.8 | 0.95 |
| Understandability | LMM | ERNIEBot x English x Expert | 0.09 | [-0.59, 0.77] | 0.26 | 0.8 | 0.95 |
| Understandability | LMM | Gemini x English x Expert | -0.4 | [-1.08, 0.28] | -1.14 | 0.25 | 0.51 |
| Understandability | LMM | Grok x English x Expert | -0.79 | [-1.47, -0.11] | -2.29 | 2.20E-02 | 0.06 |
| Understandability | LMM | Hunyuan x English x Expert | -0.34 | [-1.02, 0.34] | -0.98 | 0.33 | 0.54 |
| Understandability | LMM | Kimi x English x Expert | 0.09 | [-0.59, 0.77] | 0.26 | 0.8 | 0.95 |
| Understandability | LMM | Qwen x English x Expert | -0.14 | [-0.82, 0.54] | -0.39 | 0.69 | 0.93 |
