# Supplementary material for: Bilingual Performance of Large Language Models in Answering Consumer Health Questions in English and Chinese: Comparative Benchmark Study
Source: J Med Internet Res. 2026 Sep 29;28:e96587. doi: 10.2196/96587 (PMC13662449; doi:10.2196/96587)
Supplement: Supplementary file 6 [file jmir_v28i1e96587_app17.docx]

| **Multimedia Appendix 16. Technique for Order of Preference by Similarity to Ideal Solution (TOPSIS) Sensitivity Analysis: Composite Scores, Tiers, and Rankings Under Three Weighting Schemes** | | | | | | |
| --- | --- | --- | --- | --- | --- | --- |
| Condition | Weighting scheme | Model | TOPSIS score | 95% CI | Tier | Rank |
| Chinese Expert | Equal | DeepSeek | 0.97 | [0.82, 1.00] | Tier 1 | 1 |
| Chinese Expert | Equal | Qwen | 0.95 | [0.84, 0.98] | Tier 1 | 2 |
| Chinese Expert | Equal | ChatGPT | 0.90 | [0.79, 0.97] | Tier 1 | 3 |
| Chinese Expert | Equal | Grok | 0.75 | [0.46, 0.81] | Tier 2 | 4 |
| Chinese Expert | Equal | Doubao | 0.70 | [0.39, 0.76] | Tier 2 | 5 |
| Chinese Expert | Equal | Hunyuan | 0.70 | [0.36, 0.74] | Tier 2 | 6 |
| Chinese Expert | Equal | Gemini | 0.65 | [0.25, 0.70] | Tier 2 | 7 |
| Chinese Expert | Equal | Claude | 0.54 | [0.03, 0.58] | Tier 2 | 8 |
| Chinese Expert | Equal | Kimi | 0.40 | [0.00, 0.43] | Tier 2 | 9 |
| Chinese Expert | Equal | ERNIEBot | 0.13 | [0.00, 0.32] | Tier 3 | 10 |
| Chinese Expert | Equal | ChatGLM | 0.04 | [0.00, 0.35] | Tier 3 | 11 |
| Chinese Expert | Safety-weighted | DeepSeek | 0.91 | [0.80, 1.00] | Tier 1 | 1 |
| Chinese Expert | Safety-weighted | Qwen | 0.88 | [0.79, 0.96] | Tier 1 | 2 |
| Chinese Expert | Safety-weighted | ChatGPT | 0.86 | [0.77, 0.93] | Tier 1 | 3 |
| Chinese Expert | Safety-weighted | Grok | 0.77 | [0.54, 0.83] | Tier 1 | 4 |
| Chinese Expert | Safety-weighted | Hunyuan | 0.74 | [0.50, 0.78] | Tier 1 | 5 |
| Chinese Expert | Safety-weighted | Doubao | 0.67 | [0.37, 0.75] | Tier 2 | 6 |
| Chinese Expert | Safety-weighted | Gemini | 0.62 | [0.24, 0.70] | Tier 2 | 7 |
| Chinese Expert | Safety-weighted | Claude | 0.55 | [0.19, 0.61] | Tier 2 | 8 |
| Chinese Expert | Safety-weighted | Kimi | 0.39 | [0.00, 0.43] | Tier 2 | 9 |
| Chinese Expert | Safety-weighted | ERNIEBot | 0.12 | [0.00, 0.33] | Tier 3 | 10 |
| Chinese Expert | Safety-weighted | ChatGLM | 0.12 | [0.00, 0.34] | Tier 3 | 11 |
| Chinese Expert | Communication | DeepSeek | 0.97 | [0.80, 1.00] | Tier 1 | 1 |
| Chinese Expert | Communication | Qwen | 0.95 | [0.81, 0.98] | Tier 1 | 2 |
| Chinese Expert | Communication | ChatGPT | 0.89 | [0.78, 0.98] | Tier 1 | 3 |
| Chinese Expert | Communication | Grok | 0.74 | [0.44, 0.92] | Tier 2 | 4 |
| Chinese Expert | Communication | Doubao | 0.70 | [0.38, 0.88] | Tier 2 | 5 |
| Chinese Expert | Communication | Hunyuan | 0.69 | [0.35, 0.89] | Tier 2 | 6 |
| Chinese Expert | Communication | Gemini | 0.64 | [0.23, 0.83] | Tier 2 | 7 |
| Chinese Expert | Communication | Claude | 0.54 | [0.18, 0.70] | Tier 2 | 8 |
| Chinese Expert | Communication | Kimi | 0.40 | [0.00, 0.47] | Tier 2 | 9 |
| Chinese Expert | Communication | ERNIEBot | 0.14 | [0.00, 0.32] | Tier 3 | 10 |
| Chinese Expert | Communication | ChatGLM | 0.03 | [0.00, 0.35] | Tier 3 | 11 |
| Chinese No-context | Equal | ChatGPT | 0.95 | [0.88, 1.00] | Tier 1 | 1 |
| Chinese No-context | Equal | DeepSeek | 0.93 | [0.82, 1.00] | Tier 1 | 2 |
| Chinese No-context | Equal | Qwen | 0.90 | [0.81, 0.98] | Tier 1 | 3 |
| Chinese No-context | Equal | Doubao | 0.88 | [0.77, 0.93] | Tier 1 | 4 |
| Chinese No-context | Equal | Gemini | 0.71 | [0.24, 0.78] | Tier 2 | 5 |
| Chinese No-context | Equal | Claude | 0.70 | [0.24, 0.77] | Tier 2 | 6 |
| Chinese No-context | Equal | Hunyuan | 0.67 | [0.20, 0.73] | Tier 2 | 7 |
| Chinese No-context | Equal | Grok | 0.65 | [0.09, 0.71] | Tier 2 | 8 |
| Chinese No-context | Equal | ERNIEBot | 0.14 | [0.02, 0.37] | Tier 3 | 9 |
| Chinese No-context | Equal | Kimi | 0.07 | [0.00, 0.38] | Tier 3 | 10 |
| Chinese No-context | Equal | ChatGLM | 0.05 | [0.00, 0.39] | Tier 3 | 11 |
| Chinese No-context | Safety-weighted | ChatGPT | 0.96 | [0.88, 1.00] | Tier 1 | 1 |
| Chinese No-context | Safety-weighted | DeepSeek | 0.93 | [0.85, 1.00] | Tier 1 | 2 |
| Chinese No-context | Safety-weighted | Doubao | 0.88 | [0.79, 1.00] | Tier 1 | 3 |
| Chinese No-context | Safety-weighted | Qwen | 0.88 | [0.76, 0.95] | Tier 1 | 4 |
| Chinese No-context | Safety-weighted | Claude | 0.71 | [0.39, 0.79] | Tier 2 | 5 |
| Chinese No-context | Safety-weighted | Hunyuan | 0.68 | [0.35, 0.76] | Tier 2 | 6 |
| Chinese No-context | Safety-weighted | Gemini | 0.66 | [0.21, 0.73] | Tier 2 | 7 |
| Chinese No-context | Safety-weighted | Grok | 0.61 | [0.15, 0.68] | Tier 2 | 8 |
| Chinese No-context | Safety-weighted | ERNIEBot | 0.24 | [0.06, 0.37] | Tier 3 | 9 |
| Chinese No-context | Safety-weighted | ChatGLM | 0.13 | [0.00, 0.38] | Tier 3 | 10 |
| Chinese No-context | Safety-weighted | Kimi | 0.07 | [0.00, 0.37] | Tier 3 | 11 |
| Chinese No-context | Communication | ChatGPT | 0.95 | [0.88, 1.00] | Tier 1 | 1 |
| Chinese No-context | Communication | DeepSeek | 0.93 | [0.82, 1.00] | Tier 1 | 2 |
| Chinese No-context | Communication | Qwen | 0.91 | [0.77, 0.98] | Tier 1 | 3 |
| Chinese No-context | Communication | Doubao | 0.88 | [0.70, 1.00] | Tier 1 | 4 |
| Chinese No-context | Communication | Gemini | 0.72 | [0.25, 0.79] | Tier 2 | 5 |
| Chinese No-context | Communication | Claude | 0.70 | [0.20, 0.76] | Tier 2 | 6 |
| Chinese No-context | Communication | Hunyuan | 0.67 | [0.19, 0.73] | Tier 2 | 7 |
| Chinese No-context | Communication | Grok | 0.64 | [0.08, 0.70] | Tier 2 | 8 |
| Chinese No-context | Communication | ERNIEBot | 0.13 | [0.02, 0.37] | Tier 3 | 9 |
| Chinese No-context | Communication | Kimi | 0.07 | [0.00, 0.38] | Tier 3 | 10 |
| Chinese No-context | Communication | ChatGLM | 0.04 | [0.00, 0.39] | Tier 3 | 11 |
| English Expert | Equal | ChatGPT | 0.93 | [0.76, 0.94] | Tier 1 | 1 |
| English Expert | Equal | Qwen | 0.92 | [0.79, 1.00] | Tier 1 | 2 |
| English Expert | Equal | DeepSeek | 0.86 | [0.63, 0.97] | Tier 1 | 3 |
| English Expert | Equal | Doubao | 0.75 | [0.36, 0.83] | Tier 2 | 4 |
| English Expert | Equal | Hunyuan | 0.74 | [0.33, 0.84] | Tier 2 | 5 |
| English Expert | Equal | Claude | 0.70 | [0.29, 0.80] | Tier 2 | 6 |
| English Expert | Equal | Gemini | 0.70 | [0.25, 0.78] | Tier 2 | 7 |
| English Expert | Equal | Grok | 0.67 | [0.17, 0.75] | Tier 2 | 8 |
| English Expert | Equal | Kimi | 0.64 | [0.23, 0.73] | Tier 2 | 9 |
| English Expert | Equal | ERNIEBot | 0.26 | [0.00, 0.35] | Tier 3 | 10 |
| English Expert | Equal | ChatGLM | 0.00 | [0.00, 0.40] | Tier 3 | 11 |
| English Expert | Safety-weighted | Qwen | 0.92 | [0.83, 1.00] | Tier 1 | 1 |
| English Expert | Safety-weighted | DeepSeek | 0.84 | [0.66, 0.92] | Tier 1 | 2 |
| English Expert | Safety-weighted | ChatGPT | 0.83 | [0.62, 0.84] | Tier 1 | 3 |
| English Expert | Safety-weighted | Hunyuan | 0.75 | [0.50, 0.92] | Tier 2 | 4 |
| English Expert | Safety-weighted | Doubao | 0.72 | [0.38, 0.82] | Tier 2 | 5 |
| English Expert | Safety-weighted | Claude | 0.72 | [0.50, 0.88] | Tier 2 | 6 |
| English Expert | Safety-weighted | Kimi | 0.66 | [0.45, 0.82] | Tier 2 | 7 |
| English Expert | Safety-weighted | Gemini | 0.64 | [0.16, 0.71] | Tier 2 | 8 |
| English Expert | Safety-weighted | Grok | 0.60 | [0.09, 0.67] | Tier 2 | 9 |
| English Expert | Safety-weighted | ERNIEBot | 0.25 | [0.00, 0.34] | Tier 3 | 10 |
| English Expert | Safety-weighted | ChatGLM | 0.00 | [0.00, 0.39] | Tier 3 | 11 |
| English Expert | Communication | ChatGPT | 0.94 | [0.78, 0.95] | Tier 1 | 1 |
| English Expert | Communication | Qwen | 0.92 | [0.80, 1.00] | Tier 1 | 2 |
| English Expert | Communication | DeepSeek | 0.86 | [0.64, 0.98] | Tier 1 | 3 |
| English Expert | Communication | Doubao | 0.76 | [0.35, 0.88] | Tier 2 | 4 |
| English Expert | Communication | Hunyuan | 0.74 | [0.30, 0.89] | Tier 2 | 5 |
| English Expert | Communication | Claude | 0.70 | [0.25, 0.81] | Tier 2 | 6 |
| English Expert | Communication | Gemini | 0.70 | [0.18, 0.78] | Tier 2 | 7 |
| English Expert | Communication | Grok | 0.67 | [0.11, 0.75] | Tier 2 | 8 |
| English Expert | Communication | Kimi | 0.64 | [0.19, 0.73] | Tier 2 | 9 |
| English Expert | Communication | ERNIEBot | 0.26 | [0.00, 0.35] | Tier 3 | 10 |
| English Expert | Communication | ChatGLM | 0.00 | [0.00, 0.40] | Tier 3 | 11 |
| English No-context | Equal | ChatGPT | 0.96 | [0.88, 1.00] | Tier 1 | 1 |
| English No-context | Equal | Gemini | 0.90 | [0.85, 0.98] | Tier 1 | 2 |
| English No-context | Equal | Qwen | 0.88 | [0.83, 0.98] | Tier 1 | 3 |
| English No-context | Equal | Grok | 0.88 | [0.81, 0.96] | Tier 1 | 4 |
| English No-context | Equal | Doubao | 0.85 | [0.73, 0.94] | Tier 1 | 5 |
| English No-context | Equal | Hunyuan | 0.78 | [0.49, 0.87] | Tier 2 | 6 |
| English No-context | Equal | Claude | 0.70 | [0.29, 0.78] | Tier 2 | 7 |
| English No-context | Equal | DeepSeek | 0.70 | [0.22, 0.78] | Tier 2 | 8 |
| English No-context | Equal | Kimi | 0.23 | [0.02, 0.37] | Tier 3 | 9 |
| English No-context | Equal | ERNIEBot | 0.22 | [0.06, 0.38] | Tier 3 | 10 |
| English No-context | Equal | ChatGLM | 0.00 | [0.00, 0.40] | Tier 3 | 11 |
| English No-context | Safety-weighted | ChatGPT | 0.91 | [0.85, 1.00] | Tier 1 | 1 |
| English No-context | Safety-weighted | Grok | 0.89 | [0.82, 0.95] | Tier 1 | 2 |
| English No-context | Safety-weighted | Gemini | 0.87 | [0.83, 0.94] | Tier 1 | 3 |
| English No-context | Safety-weighted | Qwen | 0.86 | [0.81, 0.95] | Tier 1 | 4 |
| English No-context | Safety-weighted | Doubao | 0.80 | [0.75, 0.89] | Tier 1 | 5 |
| English No-context | Safety-weighted | Hunyuan | 0.79 | [0.71, 0.87] | Tier 1 | 6 |
| English No-context | Safety-weighted | Claude | 0.71 | [0.61, 0.79] | Tier 2 | 7 |
| English No-context | Safety-weighted | DeepSeek | 0.62 | [0.53, 0.69] | Tier 2 | 8 |
| English No-context | Safety-weighted | ERNIEBot | 0.29 | [0.12, 0.33] | Tier 3 | 9 |
| English No-context | Safety-weighted | Kimi | 0.25 | [0.06, 0.29] | Tier 3 | 10 |
| English No-context | Safety-weighted | ChatGLM | 0.00 | [0.00, 0.24] | Tier 3 | 11 |
| English No-context | Communication | ChatGPT | 0.97 | [0.89, 1.00] | Tier 1 | 1 |
| English No-context | Communication | Gemini | 0.90 | [0.87, 0.97] | Tier 1 | 2 |
| English No-context | Communication | Qwen | 0.89 | [0.84, 0.96] | Tier 1 | 3 |
| English No-context | Communication | Grok | 0.87 | [0.78, 0.94] | Tier 1 | 4 |
| English No-context | Communication | Doubao | 0.86 | [0.74, 0.94] | Tier 1 | 5 |
| English No-context | Communication | Hunyuan | 0.78 | [0.48, 0.87] | Tier 2 | 6 |
| English No-context | Communication | DeepSeek | 0.71 | [0.27, 0.79] | Tier 2 | 7 |
| English No-context | Communication | Claude | 0.70 | [0.29, 0.77] | Tier 2 | 8 |
| English No-context | Communication | Kimi | 0.22 | [0.02, 0.37] | Tier 3 | 9 |
| English No-context | Communication | ERNIEBot | 0.21 | [0.05, 0.37] | Tier 3 | 10 |
| English No-context | Communication | ChatGLM | 0.00 | [0.00, 0.40] | Tier 3 | 11 |
