# Supplementary material for: Bilingual Performance of Large Language Models in Answering Consumer Health Questions in English and Chinese: Comparative Benchmark Study
Source: J Med Internet Res. 2026 Sep 29;28:e96587. doi: 10.2196/96587 (PMC13662449; doi:10.2196/96587)
Supplement: Supplementary file 7 [file jmir_v28i1e96587_app18.docx]

| **Multimedia Appendix 17. Post Hoc Error Correction Rates (Δ-Accuracy) by Model and Language, with pooled counts.** | | | | | | | | | | | | | | |
| --- | --- | --- | --- | --- | --- | --- | --- | --- | --- | --- | --- | --- | --- | --- |
| Model | Language | n | Baseline accuracy, % | Baseline errors | Δ Expert (P2), % | Expert, corrected n/N | Δ CoT (P3), % | CoT, corrected n/N | Δ RAG (P4), % | RAG, corrected n/N | Δ CoT+RAG (P5), % | CoT+RAG, corrected n/N | Δ Error Attribution (P6), % | Error Attribution, corrected n/N |
| ChatGLM | Chinese | 150 | 92 | 12 | -41.67 | -5/12 | 41.67 | 5/12 | 25 | 3/12 | 33.33 | 4/12 | 58.33 | 7/12 |
| ChatGPT | Chinese | 150 | 96 | 6 | -66.67 | -4/6 | 16.67 | 1/6 | 16.67 | 1/6 | 33.33 | 2/6 | 16.67 | 1/6 |
| Claude | Chinese | 150 | 94.67 | 8 | -50 | -4/8 | 37.5 | 3/8 | 12.5 | 1/8 | 25 | 2/8 | 37.5 | 3/8 |
| DeepSeek | Chinese | 150 | 96 | 6 | -50 | -3/6 | 50 | 3/6 | 33.33 | 2/6 | 16.67 | 1/6 | 50 | 3/6 |
| Doubao | Chinese | 150 | 95.33 | 7 | -85.71 | -6/7 | 57.14 | 4/7 | 28.57 | 2/7 | 28.57 | 2/7 | 71.43 | 5/7 |
| ERNIEBot | Chinese | 150 | 93.33 | 10 | -110 | -11/10 | 70 | 7/10 | 40 | 4/10 | 40 | 4/10 | 30 | 3/10 |
| Gemini | Chinese | 150 | 92.67 | 11 | -18.18 | -2/11 | 45.45 | 5/11 | 36.36 | 4/11 | 27.27 | 3/11 | 54.55 | 6/11 |
| Grok | Chinese | 150 | 92.67 | 11 | 27.27 | 3/11 | 36.36 | 4/11 | 36.36 | 4/11 | 18.18 | 2/11 | 36.36 | 4/11 |
| Hunyuan | Chinese | 150 | 94.67 | 8 | 25 | 2/8 | 37.5 | 3/8 | 62.5 | 5/8 | 50 | 4/8 | 62.5 | 5/8 |
| Kimi | Chinese | 150 | 90 | 15 | -6.67 | -1/15 | 53.33 | 8/15 | 60 | 9/15 | 53.33 | 8/15 | 66.67 | 10/15 |
| Qwen | Chinese | 150 | 94.67 | 8 | -25 | -2/8 | 37.5 | 3/8 | 37.5 | 3/8 | 37.5 | 3/8 | 62.5 | 5/8 |
| ChatGLM | English | 150 | 92 | 12 | -41.67 | -5/12 | 66.67 | 8/12 | 50 | 6/12 | 25 | 3/12 | 75 | 9/12 |
| ChatGPT | English | 150 | 96 | 6 | -133.33 | -8/6 | 33.33 | 2/6 | 16.67 | 1/6 | 33.33 | 2/6 | 33.33 | 2/6 |
| Claude | English | 150 | 96 | 6 | -50 | -3/6 | 66.67 | 4/6 | 83.33 | 5/6 | 66.67 | 4/6 | 50 | 3/6 |
| DeepSeek | English | 150 | 93.33 | 10 | -10 | -1/10 | 60 | 6/10 | 40 | 4/10 | 50 | 5/10 | 70 | 7/10 |
| Doubao | English | 150 | 95.33 | 7 | -85.71 | -6/7 | 71.43 | 5/7 | 28.57 | 2/7 | 42.86 | 3/7 | 71.43 | 5/7 |
| ERNIEBot | English | 150 | 95.33 | 7 | -128.57 | -9/7 | 28.57 | 2/7 | 71.43 | 5/7 | 42.86 | 3/7 | 57.14 | 4/7 |
| Gemini | English | 150 | 96 | 6 | -166.67 | -10/6 | 50 | 3/6 | 0 | 0/6 | 0 | 0/6 | 33.33 | 2/6 |
| Grok | English | 150 | 97.33 | 4 | -325 | -13/4 | 25 | 1/4 | 0 | 0/4 | 25 | 1/4 | 0 | 0/4 |
| Hunyuan | English | 150 | 96.67 | 5 | -100 | -5/5 | 60 | 3/5 | 20 | 1/5 | 40 | 2/5 | 60 | 3/5 |
| Kimi | English | 150 | 94 | 9 | 0 | 0/9 | 55.56 | 5/9 | 77.78 | 7/9 | 77.78 | 7/9 | 88.89 | 8/9 |
| Qwen | English | 150 | 96 | 6 | -50 | -3/6 | 33.33 | 2/6 | 33.33 | 2/6 | 66.67 | 4/6 | 83.33 | 5/6 |
| Chinese (pooled) | |  | 93.82 | 102 | -32.35 | -33/102 | 45.1 | 46/102 | 37.25 | 38/102 | 34.31 | 35/102 | 50.98 | 52/102 |
| English (pooled) | |  | 95.27 | 78 | -80.77 | -63/78 | 52.56 | 41/78 | 42.31 | 33/78 | 43.59 | 34/78 | 61.54 | 48/78 |
| Overall (pooled) | |  | 94.55 | 180 | -53.33 | -96/180 | 48.33 | 87/180 | 39.44 | 71/180 | 38.33 | 69/180 | 55.56 | 100/180 |
| Note. Model-level rows reported each model's own correction rate; pooled rows reported Σ corrected/Σ errors across models (percentages identical to those in the main text). Δ Expert reflected the net change in errors after expert prompting (negative values = more new errors introduced than corrected). Baseline errors per cell = n × (1 − baseline accuracy). | | | | | | | | | | | | | | |
