# Supplementary material for: Bilingual Performance of Large Language Models in Answering Consumer Health Questions in English and Chinese: Comparative Benchmark Study
Source: J Med Internet Res. 2026 Sep 29;28:e96587. doi: 10.2196/96587 (PMC13662449; doi:10.2196/96587)
Supplement: Supplementary file 8 [file jmir_v28i1e96587_app19.docx]

## **Multimedia Appendix 18. Detailed prompts and Complete conversation logs of Claude Opus 4.6 (Anthropic).**

**Prompts:** As a non-native English speaker, kindly help me revise the following text for improved understanding and clarity. Please check for spelling and sentence structure errors and suggest alternatives. What suggestions do you have to enhance the clarity of my text? Please identify any parts of my writing that may be difficult for a lay audience to understand.

**Complete conversation logs:** 55处修正已应用。用Word的Review → Compare Documents（原稿 vs 这个文件）即可生成tracked changes。

**修正内容按类型汇总：**

**术语统一（5处）：** "Medical Health" → "Consumer Health" 贯穿标题、摘要、引言、关键词。"Science popularization" → "consumer health guidance"（科普的英文对应词）。

**语法修正（12处）：** 去掉"communication, has positioned"中的多余逗号；"linguisticand" → "linguistic and"（缺少空格）；"communication,yet" → "communication, yet"；"reassessedusing" → "reassessed using"；"strategy.The" → "strategy. The"；"anchors.." → "anchors."（双句号）；"( draw from2019" → "(drawn from the 2019"；"presion" → "precision"（拼写错误）；"concerned" → "concerns"。

**时态一致性（8处）：** Results部分GEE/LMM段落全部从现在时改为过去时："survive" → "survived"，"is non-significant" → "was not significant"，"reduces" → "reduced"，"shows" → "showed"等。

**P值格式（2处）：** "FDR=0.003" → "FDR P=.003"，"FDR≈0" → "FDR P<.001"（JMIR要求大写P无前导零）。

**PMID格式（8处）：** 清除所有参考文献中的脱字符号（^39874574 → PMID:39874574）。

**Data/Code Availability扩展（2处）：** 按reviewer要求扩展了数据可用性声明。

**其他（18处）：** 去掉重复句子、修正编号格式（l→1）、修正图例措辞（删除"for longitudinal analysis"）、资助声明去掉多余逗号等。

**7处未能应用的原因：** 文本跨多个XML run分布，python-docx的plain text匹配无法定位。这些是较次要的修正（如部分"critical"→"important"的替换），可以在Word中手动查找替换完成。
