# Supplementary material for: Bilingual Performance of Large Language Models in Answering Consumer Health Questions in English and Chinese: Comparative Benchmark Study
Source: J Med Internet Res. 2026 Sep 29;28:e96587. doi: 10.2196/96587 (PMC13662449; doi:10.2196/96587)
Supplement: Supplementary file 10 [file jmir_v28i1e96587_app3.docx]

**Multimedia Appendix 3. 150 questions (English + Chinese) with ground-truth labels**

1) **English questions**

# 2019 TREC

1 Can cranberries prevent urinary tract infections?

**Answer:** Effective (Cochrane: cranberry products reduce the risk of symptomatic UTIs (esp. recurrent UTI))

2 Can acupuncture be a solution to insomnia?

**Answer:** Unclear (Cochrane: insufficient evidence of benefit for insomnia)

3 Can acupuncture be effective for people with epilepsy?

**Answer:** Unclear (Cochrane: insufficient reliable evidence for epilepsy)

4 Can honey be used to heal wounds?

**Answer:** Effective (Cochrane: heals partial-thickness burns faster than conventional dressings)

5 Can acupuncture prevent migraines?

**Answer:** Effective (Cochrane: reduces the frequency of migraine attacks)

6 Is amygdalin or laetrile an effective cancer treatment?

**Answer:** Ineffective (Cochrane: not supported by clinical data; net negative risk-benefit (cyanide toxicity))

7 Can aspirin improve the lives of people with vascular dementia?

**Answer:** Unclear (Cochrane: no evidence of benefit for vascular dementia)

8 Can melatonin be used to reduce jet lag?

**Answer:** Effective (Cochrane: remarkably effective at preventing or reducing jet lag)

9 Can ear drops remove ear wax?

**Answer:** Effective (Cochrane: probably better than no treatment for clearing earwax (low-certainty))

10 Can gene therapy prevent complications caused by sickle cell disease?

**Answer:** Unclear (Cochrane: promising but no established curative therapy; very limited evidence)

11 Can exercises relieve lower back pain?

**Answer:** Effective (Cochrane: probably slightly improves pain and function in chronic low-back pain)

12 Is male circumcision helpful in reducing heterosexual men's chances of getting HIV?

**Answer:** Effective (Cochrane: reduces heterosexual acquisition of HIV by about 38-66%)

13 Find documents that discuss using antidepressants for helping to manage or relieve lower back pain.

**Answer:** Unclear (Cochrane: insufficient evidence of benefit for low-back pain)

14 Are breathing exercises helpful for children with asthma?

**Answer:** Unclear (Cochrane: insufficient evidence in children with asthma)

15 Can probiotics treat bacterial vaginosis?

**Answer:** Effective (Cochrane: may improve cure rates, especially as adjunct to antibiotics (low-certainty))

16 Can magnesium prevent muscle cramps?

**Answer:** Ineffective (Cochrane: unlikely to provide clinically meaningful relief from muscle cramps)

17 Can lumbar supports treat or prevent lower back pain?

**Answer:** Unclear (Cochrane: insufficient evidence for prevention or treatment of low-back pain)

18 Can electrical stimulation devices treat male urinary incontinence?

**Answer:** Unclear (Cochrane: insufficient evidence for male urinary incontinence)

19 Can honey offer relief from cough symptoms in children?

**Answer:** Effective (Cochrane: probably relieves cough more than no treatment, placebo or some drugs)

20 Can steroids be used as a treatment for spinal cord injury?

**Answer:** Ineffective (Cochrane: not shown to provide clinically important benefit; may raise complications)

21 Can acupuncture treat vascular dementia?

**Answer:** Unclear (Cochrane: insufficient evidence for vascular dementia)

22 How effective is hydroxyzine (also known as Atarax) for treating generalized anxiety disorder?

**Answer:** Effective (Cochrane: effective for GAD, comparable to benzodiazepines/buspirone)

23 Is insulin an effective treatment for gestational diabetes?

**Answer:** Effective (Cochrane: effective for glycaemic control in gestational diabetes)

24 Can yoga control epilepsy?

**Answer:** Unclear (Cochrane: no reliable evidence for epilepsy)

25 Can fish oil be used to maintain the remission of ulcerative colitis?

**Answer:** Unclear (Cochrane: insufficient evidence for maintaining remission)

26 Can vaccines prevent the common cold?

**Answer:** Ineffective (Cochrane: adenovirus vaccine showed no benefit; no effective common-cold vaccine)

27 Can antibiotics be used as a treatment for wet cough (productive or chesty cough) in children?

**Answer:** Ineffective (Cochrane: little benefit for persistent wet cough; not routinely recommended)

28 Can antibiotics be used as a treatment for whooping cough (pertussis)?

**Answer:** Effective (Cochrane: eradicate Bordetella pertussis and reduce contagiousness)

29 Can antibiotics be use to treat community acquired pneumonia in children?

**Answer:** Effective (Cochrane: amoxicillin effective for non-severe community-acquired pneumonia)

30 Can you apply aloe vera to treat wounds?

**Answer:** Unclear (Cochrane: insufficient evidence that aloe vera heals wounds/ulcers)

31 Are exercises helpful in reducing hot flashes and night sweats in menopausal women?

**Answer:** Unclear (Cochrane: no or only very small, uncertain effect on vasomotor symptoms)

32 Can ginkgo biloba treat tinnitus?

**Answer:** Ineffective (Cochrane: no evidence of effectiveness for primary complaint of tinnitus)

33 Does hypnotherapy help people quit smoking?

**Answer:** Ineffective (Cochrane: no greater effect on quit rates than control/no treatment)

34 How effective are pelvic floor exercises for treating female incontinence?

**Answer:** Effective (Cochrane: first-line therapy for stress and mixed urinary incontinence)

35 Is valerian an effective treatment for anxiety disorders?

**Answer:** Unclear (Cochrane: insufficient evidence for anxiety disorders)

36 Are dental sealants helpful in preventing tooth decay?

**Answer:** Effective (Cochrane: substantially reduce caries in permanent teeth)

37 Can taking laxatives help with hemorrhoids?

**Answer:** Unclear (Cochrane: insufficient evidence; possible small benefit)

38 Is lumbar traction an effective treatment for lower back pain?

**Answer:** Ineffective (Cochrane: little or no effect on pain, function or return to work)

39 Can muscle relaxants be used to relieve back pain?

**Answer:** Effective (Cochrane: effective for short-term relief of acute low-back pain)

40 Can cinnamon help people with diabetes?

**Answer:** Unclear (Cochrane: insufficient evidence of glycaemic benefit in diabetes)

41 Can benzos (benzodiazepines) help with alcohol withdrawal?

**Answer:** Effective (Cochrane: effective and preferred treatment for alcohol withdrawal)

42 Are probiotics an effective treatment for eczema?

**Answer:** Ineffective (Cochrane: not evidence-based; little or no difference vs no probiotic)

43 Are compression stockings helpful for varicose veins?

**Answer:** Unclear (Cochrane: insufficient high-quality evidence)

44 Can vitamins help manage epilepsy?

**Answer:** Unclear (Cochrane: insufficient reliable evidence for epilepsy)

45 Can caffeine help people with asthma?

**Answer:** Effective (Cochrane: modestly improves airway function for up to about four hours)

46 Can feverfew prevent migraine headaches?

**Answer:** Unclear (Cochrane: insufficient evidence; benefit below clinical relevance)

47 Can insoles treat back pain?

**Answer:** Ineffective (Cochrane: no evidence that insoles prevent or treat back pain)

48 Is acupuncture helpful for easing the symptoms of chronic asthma?

**Answer:** Unclear (Cochrane: insufficient evidence for chronic asthma)

49 Is sulfasalazine an effective treatment for rheumatoid arthritis?

**Answer:** Effective (Cochrane: effective DMARD for rheumatoid arthritis)

50 Is bariatric surgery effective for obesity?

**Answer:** Effective (Cochrane: effective for weight loss in obesity)

51 Can dehumidifiers be used to control asthma?

**Answer:** Unclear (Cochrane: no evidence of benefit (very low-certainty))

# 2021 TREC

1 Will wearing an ankle brace help heal achilles tendonitis?

**Answer:** Unhelpful

2 Is a tepid sponge bath a good way to reduce fever in children?

**Answer:** Unhelpful

3 Can folic acid help improve cognition and treat dementia?

**Answer:** Unhelpful

4 Does duct tape work for wart removal?

**Answer:** Unhelpful

5 Should I apply ice to a burn?

**Answer:** Unhelpful

6 Can vitamin b12 and sun exposure together help treat vitiligo?

**Answer:** Helpful

7 Does yoga improve the management of asthma?

**Answer:** Helpful

8 Is starving a fever effective?

**Answer:** Unhelpful

9 Does selenium help prevent cancer?

**Answer:** Unhelpful

10 Will taking birth control pills treat an ovarian cyst?

**Answer:** Unhelpful

11 Will taking zinc supplements improve pregnancy?

**Answer:** Unhelpful

12 Can evening primrose oil help treat eczema?

**Answer:** Unhelpful

13 Do ankle braces help heal an ankle fracture?

**Answer:** Helpful

14 Can applying vitamin E cream remove skin scars?

**Answer:** Unhelpful

15 Can magnesium help prevent migraine?

**Answer:** Helpful

16 Does exercise help manage diabetes?

**Answer:** Helpful

17 Can fermented milk help mitigate high blood pressure?

**Answer:** Unhelpful

18 Can dupixent treat eczema?

**Answer:** Helpful

19 Does yoga help reduce pain from arthritis and improve physical function?

**Answer:** Helpful

20 Can the drug Imitrex (sumatriptan) treat acute migraine attacks?

**Answer:** Helpful

21 Will buying a light therapy lamp help treat depression?

**Answer:** Helpful

22 Does Aleve relieve migraine headaches?

**Answer:** Unhelpful

23 Can I get rid of a pimple overnight by applying toothpaste?

**Answer:** Unhelpful

24 Can chondroitin help relieve the pain of osteoarthritis?

**Answer:** Helpful

25 Can omega-3 help manage hypertriglyceridemia?

**Answer:** Helpful

26 Can probiotics prevent respiratory infections?

**Answer:** Helpful

27 Can aromatherapy massage help manage rheumatoid arthritis?

**Answer:** Helpful

28 Does steam from a shower help croup?

**Answer:** Unhelpful

29 Can minoxidil treat hair loss?

**Answer:** Helpful

30 Is the Hoxsey treatment a good cure for cancer?

**Answer:** Unhelpful

31 Can l-theanine supplements reduce stress and anxiety?

**Answer:** Helpful

32 Does inhaling steam help treat common cold?

**Answer:** Unhelpful

33 Does exercise improve the symptoms of depression?

**Answer:** Helpful

34 Can I remove a tick by covering it with Vaseline?

**Answer:** Unhelpful

35 Can applying mayonnaise help get rid of lice?

**Answer:** Unhelpful

36 Can eating dates help manage iron deficiency anemia?

**Answer:** Helpful

37 Will drinking vinegar dissolve a stuck fish bone?

**Answer:** Unhelpful

39 Can copper bracelets reduce the pain of arthritis?

**Answer:** Unhelpful

40 Can fungal creams treat athlete's foot?

**Answer:** Helpful

41 Does cocoa butter help reduce pregnancy stretch marks?

**Answer:** Unhelpful

42 Can probiotics prevent diarrhea caused by antibiotics in children?

**Answer:** Helpful

43 Does Tylenol manage the symptoms of osteoarthritis?

**Answer:** Helpful

44 Can music therapy help manage depression?

**Answer:** Helpful

45 Can omega-3 treat borderline personality disorder in women?

**Answer:** Helpful

46 Can vitamin D supplements improve the management of asthma?

**Answer:** Helpful

47 Will feeding soy formula to my baby prevent the development of allergies?

**Answer:** Unhelpful

48 Does chitosan help lose body weight?

**Answer:** Unhelpful

49 Will at-home exercises manage hip osteoarthritis pain?

**Answer:** Helpful

50 Will taking antioxidant supplements treat fertility problems?

**Answer:** Unhelpful

# 2022 TREC

1 Do tea bags help to clot blood in pulled teeth?

**Answer:** Yes

2 Can crystals heal?

**Answer:** No

3 Can collagen supplements cure osteoarthritis?

**Answer:** No

4 Can cell phones cause cancer?

**Answer:** No

5 Can you use WD-40 for arthritis?

**Answer:** No

6 Can mosquito bites make you sick?

**Answer:** Yes

7 Can cancer be inherited?

**Answer:** Yes

8 Are vaccines linked to autism?

**Answer:** No

9 Can baking soda help to cure cancer?

**Answer:** No

10 Are squats bad for knees?

**Answer:** No

11 Do ACE inhibitors typically cause erectile dysfunction?

**Answer:** No

12 Is morphine addictive?

**Answer:** Yes

13 Can fruit juice increase the risk of diabetes?

**Answer:** Yes

14 Do magnetic wrist straps help with arthritis?

**Answer:** No

15 Are sit ups bad for you?

**Answer:** Yes

16 Can a speech problem be caused by right handed person being forced to left hand?

**Answer:** Yes

17 Are there health benefits to drinking your own urine?

**Answer:** No

18 Is pink salt good for you?

**Answer:** No

19 Can massage get rid of cellulite?

**Answer:** No

20 Can fish oil improve your cholesterol?

**Answer:** No

21 Can you become addicted to weed?

**Answer:** Yes

22 Can vegan diets be healthy?

**Answer:** Yes

23 Is hydroquinone banned in Europe?

**Answer:** Yes

24 Do men get UTI infections?

**Answer:** Yes

25 Are carrots good for your eyes?

**Answer:** Yes

26 Is methanol poisonous?

**Answer:** Yes

27 Can an MRI harm my baby?

**Answer:** No

28 Can exercise lower cholesterol?

**Answer:** Yes

29 Can chewing gum help lose weight?

**Answer:** No

30 Can sunglasses help prevent cataracts?

**Answer:** Yes

31 Did AIDS come from chimps?

**Answer:** Yes

32 Is too much water bad for you?

**Answer:** Yes

33 Can HIV be transmitted through sweat?

**Answer:** No

34 Do Himalayan salt lamps have health benefits?

**Answer:** No

35 Can a woman get pregnant while breastfeeding?

**Answer:** Yes

36 Can statins cause permanent cognitive impairment?

**Answer:** No

37 Does Vitamin C prevent colds?

**Answer:** No

38 Can coffee help you lose weight?

**Answer:** Yes

39 Does drinking lemon water help with belly fat?

**Answer:** No

40 Does deli meat increase your risk of colon cancer?

**Answer:** Yes

41 Are skin tags contagious?

**Answer:** No

42 Can oil pulling heal cavities?

**Answer:** No

43 Does a high fiber diet help with hemorrhoids?

**Answer:** Yes

44 Can grapefruit interfere with medication?

**Answer:** Yes

45 Can vape pens be harmful?

**Answer:** Yes

46 Do bananas increase the risk of diabetes?

**Answer:** No

47 Is wifi harmful for health?

**Answer:** No

48 Does drinking apple cider vinegar help lose weight?

**Answer:** No

49 Does ginger help with nausea?

**Answer:** Yes

50 Can a cold sore cause genital herpes?

**Answer:** Yes

2) **Chinese questions**

# 2019 TREC

1. 蔓越莓可以预防尿路感染吗？

**答案：**有效（Cochrane：蔓越莓制品可降低症状性尿路感染的风险（尤其是复发性感染））

2. 针灸能解决失眠问题吗？

**答案：**证据不足（Cochrane：对失眠的疗效证据不足）

3. 针灸对癫痫患者有效吗？

**答案：**证据不足（Cochrane：缺乏支持其对癫痫有效的可靠证据）

4. 蜂蜜能用于伤口愈合吗？

**答案：**有效（Cochrane：较传统敷料更快促进浅表烧伤愈合）

5. 针灸能预防偏头痛吗？

**答案：**有效（Cochrane：可减少偏头痛发作频率）

6. 苦杏仁苷或苦杏仁素是有效的癌症治疗手段吗？

**答案：**无效（Cochrane：无临床数据支持；风险-获益明显为负（有氰化物中毒风险））

7. 阿司匹林能改善血管性痴呆患者的生活质量吗？

**答案：**证据不足（Cochrane：无证据显示对血管性痴呆有益）

8. 褪黑素能缓解时差反应吗？

**答案：**有效（Cochrane：在预防或减轻时差反应方面效果显著）

9. 耳滴液能去除耳垢吗？

**答案：**有效（Cochrane：清除耳垢可能优于不处理（证据确定性低））

10. 基因疗法能预防镰状细胞病引发的并发症吗？

**答案：**证据不足（Cochrane：前景可期但尚无成熟的治愈性疗法；证据非常有限）

11. 运动能缓解下背痛吗？

**答案：**有效（Cochrane：可能轻度改善慢性下背痛的疼痛与功能）

12. 男性割包皮能降低异性恋男性的艾滋病感染几率吗？

**答案：**有效（Cochrane：可使异性恋男性获得性 HIV 感染降低约 38%–66%）

13. 查找讨论使用抗抑郁药帮助缓解下背痛的文件。

**答案：**证据不足（Cochrane：对下背痛的疗效证据不足）

14. 呼吸练习对哮喘儿童有帮助吗？

**答案：**证据不足（Cochrane：在哮喘儿童中证据不足）

15. 益生菌能治疗细菌性阴道炎吗？

**答案：**有效（Cochrane：可能提高治愈率，尤其作为抗生素辅助治疗（证据确定性低））

16. 镁能预防肌肉痉挛吗？

**答案：**无效（Cochrane：不太可能对肌肉痉挛提供有临床意义的缓解）

17. 腰部支撑能治疗或预防下背痛吗？

**答案：**证据不足（Cochrane：预防或治疗下背痛的证据不足）

18. 电刺激装置能治疗男性尿失禁吗？

**答案：**证据不足（Cochrane：对男性尿失禁的证据不足）

19. 蜂蜜能缓解儿童咳嗽症状吗？

**答案：**有效（Cochrane：缓解咳嗽可能优于不处理、安慰剂或部分药物）

20. 激素能用于治疗脊髓损伤吗？

**答案：**无效（Cochrane：未显示有临床重要获益；可能增加并发症）

21. 针灸能治疗血管性痴呆吗？

**答案：**证据不足（Cochrane：对血管性痴呆的证据不足）

22. 羟嗪治疗广泛性焦虑障碍的效果如何？

**答案：**有效（Cochrane：对广泛性焦虑障碍有效，与苯二氮䓬类/丁螺环酮相当）

23. 胰岛素是妊娠糖尿病的有效治疗方法吗？

**答案：**有效（Cochrane：可有效控制妊娠糖尿病的血糖）

24. 瑜伽能控制癫痫吗？

**答案：**证据不足（Cochrane：缺乏支持其对癫痫有效的可靠证据）

25. 鱼油能用于维持溃疡性结肠炎的缓解期吗？

**答案：**证据不足（Cochrane：维持缓解的证据不足）

26. 疫苗能预防普通感冒吗？

**答案：**无效（Cochrane：腺病毒疫苗未见获益；目前无有效的普通感冒疫苗）

27. 抗生素能用于治疗儿童湿咳（有痰或胸腔咳嗽）吗？

**答案：**无效（Cochrane：对持续性湿咳获益有限；不常规推荐）

28. 抗生素能用于治疗百日咳（白喉）吗？

**答案：**有效（Cochrane：可清除百日咳鲍特菌并降低传染性）

29. 抗生素能用于治疗社区获得性肺炎儿童吗？

**答案：**有效（Cochrane：阿莫西林对非重症社区获得性肺炎有效）

30. 芦荟能用于治疗伤口吗？

**答案：**证据不足（Cochrane：芦荟促进伤口/溃疡愈合的证据不足）

31. 运动能帮助减少更年期女性的潮热和盗汗吗？

**答案：**证据不足（Cochrane：对血管舒缩症状无效或仅有很小且不确定的作用）

32. 银杏叶能治疗耳鸣吗？

**答案：**无效（Cochrane：对以耳鸣为主诉者无有效性证据）

33. 催眠疗法能帮助人们戒烟吗？

**答案：**无效（Cochrane：戒烟成功率不优于对照或不处理）

34. 盆底肌锻炼对治疗女性尿失禁的效果如何？

**答案：**有效（Cochrane：压力性及混合性尿失禁的一线治疗）

35. 谷氨酸是治疗焦虑障碍的有效方法吗？

**答案：**证据不足（Cochrane：对焦虑障碍的证据不足）

36. 牙科封闭剂有助于预防龋齿吗？

**答案：**有效（Cochrane：可显著降低恒牙龋齿）

37. 服用缓泻剂能缓解痔疮吗？

**答案：**证据不足（Cochrane：证据不足；可能有小幅获益）

38. 腰部牵引是治疗下背痛的有效方法吗？

**答案：**无效（Cochrane：对疼痛、功能或复工几无作用）

39. 肌肉松弛剂能用于缓解背痛吗？

**答案：**有效（Cochrane：对急性下背痛的短期缓解有效）

40. 肉桂能帮助糖尿病患者吗？

**答案：**证据不足（Cochrane：对糖尿病血糖控制的证据不足）

41. 苯二氮䓬类药物能用于酒精戒断吗？

**答案：**有效（Cochrane：对酒精戒断有效且为首选治疗）

42. 益生菌是治疗湿疹的有效方法吗？

**答案：**无效（Cochrane：缺乏证据支持；与不用益生菌相比几无差别）

43. 压力袜对静脉曲张有帮助吗？

**答案：**证据不足（Cochrane：高质量证据不足）

44. 维生素能控制癫痫吗？

**答案：**证据不足（Cochrane：缺乏支持其对癫痫有效的可靠证据）

45. 咖啡因能帮助哮喘患者吗？

**答案：**有效（Cochrane：可轻度改善气道功能，持续约 4 小时）

46. 野白菊能预防偏头痛吗？

**答案：**证据不足（Cochrane：证据不足；获益未达临床意义）

47. 鞋垫能治疗背痛吗？

**答案：**无效（Cochrane：无证据显示鞋垫可预防或治疗背痛）

48. 针灸有助于缓解慢性哮喘的症状吗？

**答案：**证据不足（Cochrane：对慢性哮喘的证据不足）

49. 柳氮磺胺吡啶是治疗类风湿关节炎的有效方法吗？

**答案：**有效（Cochrane：对类风湿性关节炎有效的改善病情抗风湿药）

50. 减肥手术对肥胖有效吗？

**答案：**有效（Cochrane：对肥胖的减重有效）

51. 除湿机可以用来控制哮喘吗？

**答案：**证据不足（Cochrane：无获益证据（证据确定性极低））

# 2021 TREC

1. 戴踝关节护具有助于跟腱炎的恢复吗？

**答案：**无帮助

2. 温水海绵浴是降低儿童发热的好方法吗？

**答案：**无帮助

3. 叶酸有助于改善认知和治疗痴呆症吗？

**答案：**无帮助

4. 用胶带可以去除疣吗？

**答案：**无帮助

5. 烧伤后应立即敷冰吗？

**答案：**无帮助

6. 维生素 B12 和日晒结合可以治疗白癜风吗？

**答案：**有帮助

7. 瑜伽能改善哮喘的管理吗？

**答案：**有帮助

8. “饿退烧”有效吗？

**答案：**无帮助

9. 硒有助于预防癌症吗？

**答案：**无帮助

10. 服用避孕药可以治疗卵巢囊肿吗？

**答案：**无帮助

11. 服用锌补充剂可以改善妊娠吗？

**答案：**无帮助

12. 晚安油可以治疗湿疹吗？

**答案：**无帮助

13. 踝关节护具有助于踝关节骨折的愈合吗？

**答案：**有帮助

14. 涂抹维生素 E 软膏可以去除皮肤疤痕吗？

**答案：**无帮助

15. 镁有助于预防偏头痛吗？

**答案：**有帮助

16. 运动有助于糖尿病的管理吗？

**答案：**有帮助

17. 发酵乳制品有助于降低高血压吗？

**答案：**无帮助

18. 普利尤单抗可以治疗湿疹吗？

**答案：**有帮助

19. 瑜伽有助于减轻关节炎疼痛并改善身体功能吗？

**答案：**有帮助

20. 药物舒马曲坦可以治疗急性偏头痛发作吗？

**答案：**有帮助

21. 购买光疗灯可以治疗抑郁症吗？

**答案：**有帮助

22. 萘普生能缓解偏头痛吗？

**答案：**无帮助

23. 我可以通过在痘痘上涂抹牙膏来在一夜之间消除它吗？

**答案：**无帮助

24. 鲨鱼软骨有助于缓解骨关节炎的疼痛吗？

**答案：**有帮助

25. 欧米伽 -3 能帮助管理高甘油三酯血症吗？

**答案：**有帮助

26. 益生菌可以预防呼吸道感染吗？

**答案：**有帮助

27. 香薰按摩有助于管理类风湿关节炎吗？

**答案：**有帮助

28. 淋浴蒸汽有助于治疗喉炎吗？

**答案：**无帮助

29. 米诺地尔治疗脱发有效吗？

**答案：**有帮助

30. Hoxsey（胡克斯疗法）治疗是癌症的好疗法吗？

**答案：**无帮助

31. L-茶氨酸补充剂能减轻压力和焦虑吗？

**答案：**有帮助

32. 吸入蒸汽有助于治疗普通感冒吗？

**答案：**无帮助

33. 运动能改善抑郁症的症状吗？

**答案：**有帮助

34. 我可以用凡士林覆盖蜱虫来去除它吗？

**答案：**无帮助

35. 涂抹蛋黄酱有助于去除头虱吗？

**答案：**无帮助

36. 吃枣有助于管理缺铁性贫血吗？

**答案：**有帮助

37. 喝醋能溶解卡住的鱼刺吗？

**答案：**无帮助

39. 铜手镯可以减轻关节炎的疼痛吗？

**答案：**无帮助

40. 抗真菌乳膏能治疗脚气吗？

**答案：**有帮助

41. 可可脂有助于减少妊娠纹吗？

**答案：**无帮助

42. 益生菌可以预防儿童抗生素引起的腹泻吗？

**答案：**有帮助

43. 泰诺能管理骨关节炎的症状吗？

**答案：**有帮助

44. 音乐疗法有助于管理抑郁症吗？

**答案：**有帮助

45. 欧米伽 -3 能治疗女性边缘型人格障碍吗？

**答案：**有帮助

46. 维生素 D 补充剂能改善哮喘的管理吗？

**答案：**有帮助

47. 给婴儿喂食大豆配方奶粉可以预防过敏的发展吗？

**答案：**无帮助

48. 壳聚糖有助于减轻体重吗？

**答案：**无帮助

49. 在家锻炼可以管理髋关节骨关节炎的疼痛吗？

**答案：**有帮助

50. 服用抗氧化剂补充剂能治疗生育问题吗？

**答案：**无帮助

# 2022 TREC

1. 茶袋有助于拔牙后的止血吗？

**答案：**是

2. 水晶能治疗疾病吗？

**答案：**否

3. 胶原蛋白补充剂能治愈骨关节炎吗？

**答案：**否

4. 手机会引发癌症吗？

**答案：**否

5. 我可以用 WD-40 来治疗关节炎吗？

**答案：**否

6. 蚊子叮咬会让你生病吗？

**答案：**是

7. 癌症会遗传吗？

**答案：**是

8. 疫苗与自闭症有关吗？

**答案：**否

9. 小苏打可以帮助治愈癌症吗？

**答案：**否

10. 深蹲对膝盖有害吗？

**答案：**否

11. ACE 抑制剂通常会导致勃起功能障碍吗？

**答案：**否

12. 吗啡会上瘾吗？

**答案：**是

13. 果汁会增加糖尿病的风险吗？

**答案：**是

14. 磁性腕带有助于关节炎吗？

**答案：**否

15. 仰卧起坐对你有害吗？

**答案：**是

16. 右撇子被迫改用左手会导致语言问题吗？

**答案：**是

17. 喝自己的尿液有健康益处吗？

**答案：**否

18. 粉盐对你有好处吗？

**答案：**否

19. 按摩可以消除橘皮组织吗？

**答案：**否

20. 鱼油可以改善胆固醇水平吗？

**答案：**否

21. 你会对大麻上瘾吗？

**答案：**是

22. 素食饮食可以是健康的吗？

**答案：**是

23. 水杨酸在欧洲被禁用吗？

**答案：**是

24. 男性也会得尿路感染吗？

**答案：**是

25. 胡萝卜对眼睛有益吗？

**答案：**是

26. 甲醇有毒吗？

**答案：**是

27. 核磁共振成像会对我的宝宝造成伤害吗？

**答案：**否

28. 运动可以降低胆固醇吗？

**答案：**是

29. 嚼口香糖有助于减肥吗？

**答案：**否

30. 太阳镜有助于预防白内障吗？

**答案：**是

31. 艾滋病来源于黑猩猩吗？

**答案：**是

32. 喝太多水对你有害吗？

**答案：**是

33. 艾滋病可以通过汗液传播吗？

**答案：**否

34. 喜马拉雅盐灯有健康益处吗？

**答案：**否

35. 哺乳期妇女会怀孕吗？

**答案：**是

36. 他汀类药物会导致永久性认知障碍吗？

**答案：**否

37. 维生素 C 能预防感冒吗？

**答案：**否

38. 咖啡有助于减肥吗？

**答案：**是

39. 喝柠檬水有助于减少腹部脂肪吗？

**答案：**否

40. 加工肉类会增加患结肠癌的风险吗？

**答案：**是

41. 皮肤标签会传染吗？

**答案：**否

42. 油拉法能治疗蛀牙吗？

**答案：**否

43. 高纤维饮食有助于缓解痔疮吗？

**答案：**是

44. 葡萄柚会与药物相互作用吗？

**答案：**是

45. 吸烟笔有害吗？

**答案：**是

46. 吃香蕉会增加患糖尿病的风险吗？

**答案：**否

47. 无线网络对健康有害吗？

**答案：**否

48. 喝苹果醋有助于减肥吗？

**答案：**否

49. 姜有助于缓解恶心吗？

**答案：**是

50. 口腔疱疹会导致生殖器疱疹吗？

**答案：**是
