# Supplementary material for: Bilingual Performance of Large Language Models in Answering Consumer Health Questions in English and Chinese: Comparative Benchmark Study
Source: J Med Internet Res. 2026 Sep 29;28:e96587. doi: 10.2196/96587 (PMC13662449; doi:10.2196/96587)
Supplement: Supplementary file 11 [file jmir_v28i1e96587_app4.docx]

# **Multimedia Appendix 4. Detailed prompts both in Chinese and English**

a) Prompts in English: (1) Expert prompt: "Suppose you are a committee of leading scientific experts and medical doctors reviewing the latest and highest quality research. Here is the question: [question]. Choose 'yes' or 'no' based on your best understanding of current medical practice and literature. If you do not know, you can state that you do not know." (2) Chain-of-thought (CoT) prompt: "You will answer the following health question. First, think step by step and provide your reasoning process. Then output a final answer using only one word: “yes” or “no”. Question:[question]. Please follow these steps: 1) Identify the key components and requirements of the question. 2) Break down the reasoning process into logical steps.3) Check each step for potential errors or assumptions. 4) Explain how each piece of information leads to the conclusion. 5) Provide the final reasoned answer. Format: Reasoning: <your chain-of-thought> . Final answer: yes/no. " (3) Retrieval-augmented generation (RAG) prompt: "You will answer the following health question based only on the evidence provided. Do not use your internal knowledge unless needed to interpret the evidence. If the evidence supports a clear yes/no answer, decide accordingly. Output only “yes” or “no”. Evidence: [evidence]. Question: [question]. " (4) Combined CoT + RAG prompt: "You will answer the following health question. First, analyze the evidence step by step and explain how it supports or contradicts the possible answers. Then provide your final answer using only “yes” or “no”. Evidence:[evidence]. Task: Please answer the question by following these steps (Chain of Thought):1) Analyze the Evidence: Carefully review the provided evidence. Does it directly address the question? Does it support a "Yes" or "No" answer?2) Contrast with Internal Knowledge: Compare the evidence with your internal medical knowledge. Are there conflicts? Does the evidence align with established medical consensus?3) Reasoning: Explain your logic step-by-step. If the evidence is insufficient or contradictory, prioritize the safest and most scientifically established medical consensus.4) Conclusion: Conclude with a definitive "Yes" or "No". Question: [question].Format: Thinking Process: [Your step-by-step reasoning here]. Final Answer: [Yes / No]. " (5) Error attribution prompt: "Question: [question]. You previously answered this question incorrectly. Retrieved context. Task: 1) reasoning chain: Show your step-by-step reasoning process. 2) evidence integration: Integrate evidence from the retrieved context. 3) error analysis: Identify the possible cause of the previous mistake: misunderstanding of medical knowledge, reasoning error, ambiguous question interpretation, lack of external evidence, hallucination. Explain in 2–3 sentences.4) final answer: Provide the corrected, well-supported answer. Format: Reasoning Steps: [Your step-by-step reasoning here]. Evidence Used: [evidence]. Error Analysis; Initial error; Correction. Final Answer. "
b) Prompts in Chinese: (1)专家提示词：“假定你是一个特别专业的医生和科学家，正在审核最新的高质量的医学研究文章。这是问题：[问题]。对于每个问题，基于你对医疗实践和文献的理解，你需要给一个答案，不论是对还是说错。如果你不知道，你可以直接说你不知道。”(2)思维链提示词:“请回答下面的健康问题。请先逐步展示你的推理过程，然后给出最终答案（只能是“是”或“否”）。问题：[问题]。请按照以下步骤进行：1）识别问题的关键要素和要求。2）将推理过程分解为逻辑步骤。3）检查每一步是否存在错误或假设。4）解释每条信息如何导向结论。5）提供最终经过推理的答案。格式：推理过程。最终答案：是/否。” (3)RAG提示词:“请仅根据提供的证据回答下面的健康问题。除非为了理解证据内容，否则不要使用你模型内部的知识。如果证据支持明确的“是/否”，请作答。最终答案只能是“是”或“否”。 问题：[问题]。” (4)思维链和RAG提示词:“请回答下面的健康问题。首先逐步分析证据，说明它如何支持或反驳潜在答案；然后给出最终答案（只能回答“是”或“否”）。证据：[证据]。任务：请按照以下步骤（思维链）回答问题：1）分析证据：仔细审阅提供的参考证据。这些证据是否直接回答了问题？它们倾向于支持“是”还是“否”？2）对比内部知识：将这些证据与你内部的医学知识进行对比。是否存在冲突？这些证据是否符合主流的医学共识？3）推理过程：请一步步解释你的推理逻辑。如果证据不足或相互矛盾，请优先考虑最安全、最科学的医学共识。4）结论：给出一个明确的“是”或“否”作为结论。问题：[问题]。格式：推理过程。最终答案：是/否 ”（5）归因分析提示词：“问题：{问题}。你之前回答这个问题是错误的。现在请进行错误分析：检索上下文。任务：1） 推理链：展示逐步推理过程2） 证据整合：整合检索上下文中的证据3） 错误分析：识别错误原因（可多选）：医学知识理解错误,推理过程错误，误解问题含义，缺乏外部证据，语言模型幻觉。用 2–3 句解释原因。4）最终答案：提供修正后的、有充分支持的答案。格式：推理步骤：使用的证据：[证据]。错误分析。初始错误。修正。最终答案。”
