# Supplementary material for: Bilingual Performance of Large Language Models in Answering Consumer Health Questions in English and Chinese: Comparative Benchmark Study
Source: J Med Internet Res. 2026 Sep 29;28:e96587. doi: 10.2196/96587 (PMC13662449; doi:10.2196/96587)
Supplement: Supplementary file 12 [file jmir_v28i1e96587_app5.docx]

# **Multimedia Appendix 5. Full scoring rubric with anchors and examples1-3/4-6/7-9/10 anchors for each metric**

1) **Metric 1: Accuracy (Binary)**

**Definition:** Factual correctness of the binary (yes/no) answer relative to the TREC Health Misinformation Track ground-truth label.

Scale: Correct (1) or Incorrect (0)

**Scoring rules:**

• Correct: The response provides a clear yes/no answer that matches the ground-truth label.

• Incorrect: The response provides a yes/no answer that contradicts the ground-truth label.

• Incorrect: The response states "I do not know" or explicitly refuses to answer.

• Ambiguous: If the response contains both yes and no statements, score based on the predominant conclusion. If no predominant conclusion can be identified, score as incorrect.

• Both raters independently assessed ambiguous responses; disagreements were resolved through discussion.

**Example (Correct):**

*Q: Can cranberries prevent urinary tract infections? Ground truth: Yes*

A: "Yes. Cranberry products, particularly cranberry supplements, have been shown in clinical trials to reduce the risk of recurrent urinary tract infections, especially in women. A 2023 Cochrane review found moderate-certainty evidence supporting their preventive effect."

Score: Correct. The answer is "Yes," matching the ground truth.

**Example (Incorrect):**

*Q: Can cranberries prevent urinary tract infections? Ground truth: Yes*

A: "No. While cranberries are widely believed to prevent UTIs, the evidence is mixed and most systematic reviews have concluded that cranberry products do not significantly reduce the incidence of symptomatic urinary tract infections."

Score: Incorrect. The answer is "No," contradicting the ground truth.

2) **Metric: Comprehensiveness (1-10)**

**Definition:** The scope and depth of the response, evaluating whether it addresses the question fully with appropriate context, supporting evidence, relevant caveats, and practical guidance for a lay audience.

**Score 1-3: Minimal**

**Criteria:**

• Answers yes/no with little or no explanation

• Provides no context, evidence, or caveats

• Omits key information a patient would need

• Response is a single sentence or bare statement

**Example:**

*Q: Can garlic lower blood pressure?*

A: "Yes, garlic can help lower blood pressure."

[No mechanism, no evidence, no dosage, no caveats about medication interactions.]

**Score 4-6: Moderate**

**Criteria:**

• Provides some explanation but misses key context

• Mentions evidence but without specificity (eg, no study details)

• Covers the main point but omits important nuances (dosage, duration, population specificity)

• May lack caveats or practical guidance

**Example:**

*Q: Can garlic lower blood pressure?*

A: "Yes. Some studies suggest that garlic supplements may help reduce blood pressure modestly, particularly in people with high blood pressure. However, garlic should not be used as a substitute for prescribed medication. Consult your doctor before starting any supplement."

[Provides basic explanation and caveat, but no specific evidence strength, no dosage range, no mention of interactions.]

**Score 7-9: High**

**Criteria:**

• Provides thorough explanation with appropriate context

• Cites specific evidence (meta-analyses, clinical trials) or guidelines

• Addresses caveats, limitations, or populations where evidence differs

• Includes practical guidance (eg, dosage ranges, duration, when to see a doctor)

• Well-structured for lay comprehension

**Example:**

*Q: Can garlic lower blood pressure?*

A: "Yes. Multiple meta-analyses have found that garlic supplementation can reduce systolic blood pressure by approximately 3-8 mmHg in individuals with hypertension. Aged garlic extract at doses of 600-1200 mg/day has shown the most consistent results in trials lasting 8-12 weeks. However, the effect is modest and should not replace prescribed antihypertensive medications. Garlic may interact with blood-thinning medications such as warfarin. People considering garlic supplements should discuss this with their healthcare provider, particularly if taking other medications."

**Score 10: Exceptional**

**Criteria:**

• All criteria for 7-9 met, plus:

• Addresses the question from multiple relevant angles

• Anticipates follow-up questions a patient might have

• Provides actionable, balanced, and clearly organized information

• Would serve as a suitable standalone patient education resource

**Example:**

*Q: Can garlic lower blood pressure?*

A: "Yes, there is moderate evidence that garlic supplements can lower blood pressure. A 2020 meta-analysis of 12 randomized controlled trials (553 participants) found that garlic supplementation reduced systolic BP by 8.3 mmHg and diastolic BP by 5.5 mmHg compared with placebo, primarily in hypertensive individuals. The most studied form is aged garlic extract at 600-1200 mg/day for 8-24 weeks. Raw garlic (approximately 4 g/day) has also shown benefit, though with less consistent evidence. Important considerations: (1) garlic supplements should complement, not replace, prescribed medications; (2) garlic may increase bleeding risk, especially with warfarin or aspirin; (3) gastrointestinal side effects (heartburn, nausea) are common; (4) the blood pressure reduction, while statistically significant, is modest compared with standard medications. Speak with your doctor before starting garlic supplements, especially if you are on blood pressure or blood-thinning medication."

3) **Metric: Precision (1-10)**

**Definition:** The exactness and pertinence of the content, evaluating whether the response directly addresses the question without containing hallucinated, tangential, or redundant information.

**Score 1-3: Low**

**Criteria:**

• Contains factual errors or hallucinated information

• Response is largely tangential or off-topic

• Includes misleading claims not supported by evidence

• Mixes correct and incorrect information without distinction

**Example:**

*Q: Is melatonin safe for children?*

A: "Melatonin is completely safe for children of all ages and can be given daily without any concerns. The FDA has approved melatonin for pediatric use and recommends it as a first-line treatment for childhood insomnia."

[Hallucinated: FDA has not approved melatonin for pediatric use; melatonin is a dietary supplement. Overclaims safety without noting dose-dependent effects or lack of long-term data.]

**Score 4-6: Moderate**

**Criteria:**

• Mostly accurate but includes some tangential content

• Provides relevant information but with unnecessary padding

• Minor inaccuracies or imprecise language that could mislead

• Addresses the question but drifts into loosely related topics

**Example:**

*Q: Is melatonin safe for children?*

A: "Melatonin is generally considered safe for short-term use in children. It is a hormone naturally produced by the body to regulate sleep. Many parents use melatonin to help their children fall asleep. Sleep is very important for child development, as it affects growth, learning, and behavior. Poor sleep can lead to problems at school and with mood. Melatonin supplements are available over the counter in most countries."

[Drifts into general sleep importance rather than directly answering safety. Lacks specific dosage, duration, or risk information.]

**Score 7-9: High**

**Criteria:**

• Directly addresses the question without tangential content

• All factual claims are accurate and well-supported

• No hallucinated information

• Appropriately qualified (eg, distinguishes strong vs weak evidence)

• Concise without sacrificing necessary detail

**Example:**

*Q: Is melatonin safe for children?*

A: "Melatonin is generally considered safe for short-term use in children with sleep difficulties, particularly those with neurodevelopmental conditions such as autism or ADHD. The American Academy of Pediatrics notes that melatonin can be helpful when combined with good sleep hygiene. Typical doses in clinical studies range from 0.5-5 mg, taken 30-60 minutes before bedtime. However, long-term safety data in children are limited, and melatonin is classified as a dietary supplement in the US, meaning it is not subject to the same regulatory oversight as prescription medications. Side effects may include morning drowsiness, headache, and vivid dreams. Parents should consult a pediatrician before use."

**Score 10: Exceptional**

**Criteria:**

• All criteria for 7-9 met, plus:

• Every claim is precisely calibrated to current evidence

• No unnecessary or redundant content

• Distinguishes established facts from areas of uncertainty

• Response reads as if written by a subject-matter expert for a lay reader

**Example:**

*Q: Is melatonin safe for children?*

A: "Short-term melatonin use (up to 3 months) appears safe for most children, based on systematic reviews of randomized trials. It is most studied in children with neurodevelopmental disorders (autism, ADHD) and delayed sleep-wake phase disorder, where doses of 1-5 mg reduced sleep onset latency by approximately 20-30 minutes. The American Academy of Sleep Medicine conditionally recommends melatonin for children with these conditions. Two important caveats: (1) melatonin is sold as a dietary supplement in the US, with studies showing actual content varying 83% to 478% from labeled doses across brands; (2) long-term effects on pubertal development remain understudied, with some animal data raising theoretical concerns. The European Medicines Agency has approved a prolonged-release formulation for children aged 2+ with ASD, reflecting a higher regulatory standard than the US supplement market. Consult your child's pediatrician for personalized dosing."

4) **Metric: Understandability (1-10)**

**Definition:** The clarity and accessibility of the response for a lay audience, drawing on principles from the Patient Education Materials Assessment Tool (PEMAT), including use of everyday language, logical organization, and avoidance of unexplained jargon.

**Score 1-3: Poor**

**Criteria:**

• Heavy use of unexplained medical jargon or technical terms

• Sentence structure is convoluted or difficult to follow

• Poor logical organization (ideas jump between topics)

• A lay reader would struggle to extract the key message

• May require medical training to comprehend

**Example:**

*Q: Does aspirin reduce the risk of heart attack?*

A: "Acetylsalicylic acid exerts antiplatelet effects via irreversible cyclooxygenase-1 inhibition, attenuating thromboxane A2-mediated platelet aggregation. In the context of secondary prevention of atherosclerotic cardiovascular events, low-dose ASA (75-100mg) demonstrates NNT of approximately 67 for MACE reduction over 5 years, though the risk-benefit calculus shifts unfavorably in primary prevention cohorts with low baseline CVD risk given hemorrhagic sequelae."

[Dense clinical language that a lay reader could not understand. No everyday explanations provided.]

**Score 4-6: Fair**

**Criteria:**

• Mix of lay and technical language

• Some medical terms used without adequate explanation

• Generally readable but requires above-average health literacy

• Organization is adequate but could be clearer

• Key message is identifiable but buried in complex phrasing

**Example:**

*Q: Does aspirin reduce the risk of heart attack?*

A: "Yes, aspirin can reduce the risk of heart attack. It works as an antiplatelet agent that inhibits platelet aggregation. For patients with established cardiovascular disease, low-dose aspirin (75-100 mg daily) is recommended for secondary prevention. However, for primary prevention, the benefits must be weighed against the risk of gastrointestinal bleeding and hemorrhagic stroke. Current guidelines from the USPSTF recommend against initiating low-dose aspirin for primary prevention in adults over 60."

[Terms like 'antiplatelet agent,' 'platelet aggregation,' 'secondary prevention,' 'USPSTF' may confuse lay readers.]

**Score 7-9: Good**

**Criteria:**

• Uses everyday language throughout

• Medical terms are explained when used

• Logically organized with clear flow

• A person with average health literacy could understand the response

• Key message is clear and easy to locate

**Example:**

*Q: Does aspirin reduce the risk of heart attack?*

A: "Yes, aspirin can help reduce the risk of heart attack for some people. Aspirin works by making blood platelets less sticky, which helps prevent blood clots that can block arteries and cause heart attacks. If you have already had a heart attack or been diagnosed with heart disease, your doctor may recommend taking a low dose of aspirin (usually 75-100 mg) daily to lower the chance of another event. However, aspirin is not recommended for everyone. For people who have not been diagnosed with heart disease, the potential benefit must be weighed against the risk of side effects, especially stomach bleeding. You should not start taking aspirin regularly without first talking to your doctor."

**Score 10: Excellent**

**Criteria:**

• All criteria for 7-9 met, plus:

• Could be given directly to a patient as an educational handout

• Uses analogies or examples to make complex concepts accessible

• Clear action items (what to do, when to see a doctor)

• Logical structure that guides the reader step by step

• No unexplained abbreviations or acronyms

**Example:**

*Q: Does aspirin reduce the risk of heart attack?*

A: "Yes, but it depends on your situation. Here is what you should know: How it works: Aspirin helps prevent blood clots. Think of it like making your blood slightly less sticky, so clots are less likely to form and block the arteries feeding your heart. If you already have heart disease: Your doctor may recommend a daily low-dose aspirin (about the size of a baby aspirin, 75-100 mg) to lower your chances of having another heart attack or stroke. In this situation, the benefits usually outweigh the risks. If you are healthy with no heart disease: Recent guidelines advise against routinely starting aspirin just for prevention, especially if you are over 60. The reason is that aspirin can irritate the stomach lining and increase the risk of bleeding, which may outweigh the benefit if your heart attack risk is already low. What to do: Do not start or stop taking daily aspirin without talking to your doctor. They can assess your personal risk factors and help you decide whether the benefits outweigh the risks for you."
