# Supplementary material for: Bilingual Performance of Large Language Models in Answering Consumer Health Questions in English and Chinese: Comparative Benchmark Study
Source: J Med Internet Res. 2026 Sep 29;28:e96587. doi: 10.2196/96587 (PMC13662449; doi:10.2196/96587)
Supplement: Supplementary file 13 [file jmir_v28i1e96587_app6.docx]

| **Multimedia Appendix 6. Representative Retrieval-Augmented Generation Search Logs** | | | | | | | | | | |
| --- | --- | --- | --- | --- | --- | --- | --- | --- | --- | --- |
| Year | Language | No. | Date | Query | URL 1 | URL 2 | URL 3 | Selected passages 1 | Selected passages 2 | Selected passages 3 |
| 2019 | Chinese | 3 | May,2025 | 针灸对癫痫患者有效吗？ | https://www.caam.cn/article/823 | https://www.hanspub.org/journal/paperinformation?paperid=104823 | https://www.cochrane.org/zh-hans/evidence/CD005062_acupuncture-epilepsy | 在目前的系统综述中纳入了包含1538名受试者在内的17项随机对照试验（2013年6月3日进行的文献检索）。与中药相比，针刺加中药对癫痫发作控制效果不理想（癫痫发作次数至少减少50%称之为理想）。在被纳入的试验中，针刺治疗组与对照治疗组相比，针刺治疗组并没有过多的不良事件发生。然而，纳入的试验数量少，异质性高，且有很高的偏倚风险。针刺治疗癫痫患者是否有效和安全尚不确定。 | 针灸治疗癫痫发作，现代较早的报道在五十年代初。之后，临床资料逐渐增多，科学性不断加强，五十年代末就有单位对针刺与癫痫脑电图之间的关系作了初步探讨。特别是近十馀年来，各种穴位刺激的方法，如雨后春笋般涌现，在控制癫痫发作的持续时间和延长间歇时间方面，有较好的作用，特别对于预防癫痫发作减少发作的频次，也有一定效果。在所有的穴位剌激方法中以穴位埋线应用最为普遍。目前，总有效率在80％左右。 | 针灸治疗癫痫病多取头颈项部穴位和阳经穴位以调摄阳气，进而安神、养神、守神；核心穴位为百会、内关、太冲、丰隆、神门，并根据态靶辨证，随态辨治，随靶加减，为临床进行多途径、多环节、多靶点治疗提供参考依据。 |
| 2019 | Chinese | 7 | May,2025 | 阿司匹林能改善血管性痴呆患者的生活质量吗？ | https://www.cochrane.org/zh-hans/evidence/CD011459_aspirin-and-other-non-steroidal-anti-inflammatory-drugs-prevention-dementia | https://nursing.medsci.cn/article/show_article.do?id=4bf3190993fe | https://zhuanlan.zhihu.com/p/665227985 | 我们检索了截止于2020年1月发表的相关研究。我们找到了4项符合本综述纳入标准的试验（涉及23187名受试者）。使用阿司匹林的研究发现，在健康老人中，服用低剂量阿司匹林（每日100毫克）不能预防痴呆，且会增加死亡率和严重出血风险（与安慰剂相比）。本综述没有发现证据可以支持使用阿司匹林或其他NSAIDs预防痴呆。实际上，一些意见甚至认为它们可能造成伤害。这些研究存在局限性，但出于对安全性的担忧，进一步研究低剂量阿司匹林对痴呆的预防似乎不太可能。 | 该研究共有19114名参与者接受了中位数为4.7年的随访，964名参与者接受了进一步的痴呆评估。确诊痴呆患者有575例，41%为临床可能AD。阿斯匹林与安慰剂组所有痴呆诱因(风险比[HR]为1.03;95%置信区间[CI]为0.91-1.17)、可能AD (HR为0.96;95%CI为0.74-1.24)或MCI(HR为1.12;95%CI为0.92-1.37)的风险无显著差异。随着时间的推移，阿司匹林组和安慰剂组的认知变化相似。由此可见，没有证据表明阿司匹林能有效降低痴呆、MCI或认知能力下降的风险。 | 研究发现，服用阿司匹林的老年人患老年痴呆的可能性明显降低。科学家经过进一步的研究发现，小剂量的阿司匹林还可以减慢老年痴呆症的恶化，这是因为阿司匹林可以增强脑中的血流量，起到防止血液凝固的作用。 |
| 2019 | Chinese | 17 | May,2025 | 腰部支撑能治疗或预防下背痛吗？ | https://www.mayoclinic.org/zh-hans/diseases-conditions/back-pain/symptoms-causes/syc-20369906 | https://www.msdmanuals.cn/home/bone-joint-and-muscle-disorders/low-back-and-neck-pain/low-back-pain | https://zt.emblemhealth.com/blog/health/eight-tips-prevent-back-pain | 改善自身身体状况，学习和练习如何使用身体，可能有助于预防背痛。为了保持背部健康强壮：运动。经常进行低强度有氧运动能够增强背部力量和耐力，以及改善肌肉功能。散步、骑自行车和游泳都是不错的选择，因为它们不会使背部拉紧或震动。请向医疗护理团队咨询尝试哪些运动。增强肌肉力量和柔韧性。腹肌和背肌锻炼可以增强身体核心力量，有助于调理这些肌肉，使之共同支撑背部。保持健康体重。超重会使背肌劳损。戒烟。抽烟会增加下背疼痛的风险。该风险会随着每天抽烟的数量而增加，因此戒烟应该有助于降低风险。由于背痛很常见，所以有多种药物可预防或缓解背痛。但是尚无确切的证据表明特制鞋、鞋垫、背部支撑物或专门设计的家具能有所帮助。 | 预防下背痛的最有效方法是规律锻炼。有氧运动和特殊肌肉强化以及伸展性训练是有帮助的。有氧运动，比如游泳和散步，可增进全身的健康，通常也可增强肌肉的力量。保持良好的坐姿和站姿可减少背部的压力，应避免懒散的姿势。座位的高度应该调整到脚平放到地上并且膝关节稍垂直弯曲，下背部平靠椅背。如果椅子不能支撑下背部，应该在下背部垫一个枕头。坐着时应双脚着地，不要将一条腿搭在另一条上面。应避免久站或久坐。如果无法避免长时间坐或站，经常更换姿势可以减少背部的压力。学习正确的提举姿势有助于预防背部损伤。髋关节应与肩关节保持平齐（也即不要旋转至任何一侧）。不应该在双腿几乎伸直的情况下，弯腰伸手提起物体。而是应该同时弯曲髋关节和膝关节。这样弯曲可使后背更加直立，使双臂垂下靠近物体，双肘位于物体侧方。然后，在保持物体与身体靠近的情况下，通过伸直双腿提起物体。这样的话，提起物体靠的是腿部力量而非背部力量。将物体举高过头或举起时扭身，会增加背部受伤的风险。 | 您可以做一些事情来保持您的背部健康。以下是由 WebMD 和梅奥诊所提供的8个建议：锻炼。经常运动有助于缓解肌肉紧张。有规律的、低强度的运动，比如散步和游泳，通常都是不错的选择。寻找六块腹肌。我们说的是您的腹肌！一个健美的肌肉（腹部和背部肌肉）就像您的背部天然的束身衣。注意您的体重。额外的体重会使您的下背部肌肉紧张。站直。良好的姿势可以帮助您保持背部的自然曲线，保持它的强壮。明智的坐姿。选择椅背笔直或下背部有良好支撑的座位。让您的腿发挥作用。举重物时，背部挺直，双膝弯曲。不要扭曲！如果可以的话，推而不是拉重物。扔掉高跟鞋。它们对您的下背部不好。改变您的睡眠方式。侧睡，膝盖微微向胸部收起来，如果您有背痛，可能会有帮助。 |
| 2019 | Chinese | 18 | May,2025 | 电刺激装置能治疗男性尿失禁吗？ | https://www.mayoclinic.org/zh-hans/diseases-conditions/urinary-incontinence/diagnosis-treatment/drc-20352814 | https://www.bjcyh.com.cn/Html/News/Articles/11149.html | https://www.parkwayeast.com.sg/zh/conditions-diseases/urinary-incontinence/diagnosis-treatment | 尿失禁的治疗取决于失禁的类型、严重程度和基础病因。可能需要联合治疗。如果是基础状况引起症状，医生将会先治疗该状况。医生可能会建议首先采用侵入性较小的治疗方法，如果这些方法对您没有帮助，才会转向其他选项。医生会将电极暂时插入您的直肠或阴道，以刺激和增强盆底肌。温和电刺激可有效治疗压力性尿失禁和急迫性尿失禁，但您可能需要在数月内进行多次治疗。 | 骶神经电刺激又叫"膀胱起搏器"。是近7,8年以来在欧美新近流行的微创治疗手段，去年以来，逐步在中国大陆开展工作。其主要适应症（FDA推荐适应症）：包括顽固性尿频，尿急，急迫性尿失禁，非梗阻性潴留等功能性下尿路排尿功能障碍。在适应症选择准确的前提下，能够极有效的治疗这类"疑难杂症"。 | 尿失禁的治疗将取决于其类型、严重程度和病因。由感染、肾结石或药物引起的急性尿失禁通常在治疗原始问题后便会消退。其他治疗包括：保守管理。生物反馈，有助于改善膀胱控制力。电刺激，对于压力性尿失禁或急迫性尿失禁可增强肌肉力量。凯格尔练习，增强盆底肌和括约肌力量。 |
| 2019 | Chinese | 20 | May,2025 | 激素能用于治疗脊髓损伤吗？ | https://e.dxy.cn/wisdom/front/zhihuihao/5294 | https://www.cn-healthcare.com/articlewm/20190321/content-1048008.html | https://www.ebiotrade.com/newsf/2025-10/20251018003234599.htm | 急性脊髓损伤对患者而言往往是灾难性的。目前临床上对脊髓损伤的治疗尚缺乏特效药物。激素作为具有炎症抑制，细胞保护的一类药物在以往脊髓损伤的临床治疗中扮演了非常重要的角色。但近些年，随着既往奠定激素治疗脊髓损伤基本地位的NASCIS多期临床试验研究结果被质疑，激素治疗急性脊髓损伤的基本地位已经有所动摇。Barrett S. Boody 博士观点：激素不应当应用于成年（大于18岁）不完全性脊髓损伤患者中。过去十余年的研究表明，使用激素治疗急性脊髓损伤仅能获得相当有限的效果，但伴随着较为严重的副反应。因此在临床中使用激素（如甲强龙）需权衡使用激素所可能带来的风险和收益。Jason C. Eck 博士观点：激素应该应用于成人不完全性脊髓损伤患者中。 | 脊髓疾病是神经科的常见病、多发病，以脊髓炎、视神经脊髓炎谱系疾病多见，这些免疫相关的脊髓疾病往往表现为长节段横贯性脊髓损伤，治疗以大剂量糖皮质激素为主，经激素治疗后，症状往往明显缓解或完全恢复。然而，并不是所有的长节段脊髓损伤经激素冲击治疗后都能得到缓解，有些反而会加重。 | 甲基强的松龙（MP）曾因抑制脂质过氧化和炎症介质释放被列为急性SCI标准疗法，但其高剂量方案（>5000 mg）虽能减轻脊髓水肿，却显著增加感染、高血糖和胃肠道出血风险。 |
| 2019 | Chinese | 21 | May,2025 | 针灸能治疗血管性痴呆吗？ | https://m-mip.39.net/askar/mipso_b392jwg.html | https://www.cn-healthcare.com/articlewm/20211105/content-1281632.html | https://m.thepaper.cn/newsDetail_forward_26313961 | 对血管性痴呆可以通过针灸来改善患者的认知功能，还可起到辅助治疗的效果。血管性痴呆一般是指由于血管病变导致脑缺血、缺氧、脑细胞坏死，从而引起记忆、注意、执行功能等认知功能下降的一种疾病。在对血管性痴呆患者进行针灸治疗时，一般可以选取督脉、头皮部位，也可同时配合人中、内关等穴位进行针灸。因为血管性痴呆患者存在脑缺血、缺氧以及脑细胞坏死的情况，通过针灸可以促进脑部血液循环，改善大脑供血不足的情况，从而改善患者的认知功能。 | 目前市面上使用较多的有奥拉西坦联合乐脉颗粒治疗、多奈哌齐联合脑血康治疗等。都证明中西药联合。临床上人们还采取针药结合的治疗方式（中医的针刺手法联合尼莫地平）针法醒脑开窍、滋补肝肾，药物改善血液循环，治疗效果比针药单独治疗效果显著，可有效提升患者认知功能，减轻痴呆症状。 | 越来越多的研究显示，在中医治疗中，各种神经退行性疾病属于针灸治疗的"优势病种"，越来越多的证据支持针灸对包括血管性痴呆、抑郁症和失眠症在内的各种神经精神疾病具有令人满意的疗效。针灸治疗的原则为填精益髓、醒脑调神，取督脉穴为主。主穴常取百会、四神聪、风府、太溪、悬钟、足三里，在配穴上，髓海不充配肾俞，脾肾亏虚配脾俞、肾俞，痰浊蒙窍配丰隆；气滞血瘀配膈俞、内关。除了常规针刺治疗，百会穴针后会加用灸法治疗。其他疗法还包括头针、耳针和穴位注射。 |
| 2019 | Chinese | 43 | May,2025 | 压力袜对静脉曲张有帮助吗？ | https://www.cochrane.org/zh-hans/evidence/CD008819_wearing-stockings-provide-compression-treatment-varicose-veins | https://www.sigvaris.com/zh-cn/expertise/basics/myths-and-misconceptions | https://www.drsmilehealth.com/archives/13017 | 在本综述中，我们纳入了13项研究，涉及1021名静脉曲张的受试者，他们没有经历过静脉溃疡（从2020年5月检索）。五项研究评估了加压长袜和不加压袜（例如，没有治疗或没有渐变弹性的安慰剂长袜）。在评估这一结局的九项研究中，受试者认为他们的症状在穿长袜时有所改善，但这些评估并不总是通过将同一研究中的一个试验组与对照组进行比较来做出的，因此存在偏倚。一项评估生活品质的研究显示，在穿长袜的和不穿长袜的组之间没有明显的区别。由于纳入的研究没有得出决定性的结果，因此无法就加压袜的最佳长度或压力做出任何结论。现有试验的质量证据不足以确定加压长袜能否在早期阶段作为专门和初步治疗来有效地管理和治疗静脉曲张。 | 谣传1：压力袜只适合有健康问题的人。的确，压力袜有助于预防蜘蛛静脉和静脉曲张，并有助于治疗水肿，以及其他慢性静脉疾病。对于患有某些疾病（例如糖尿病、静脉炎和腿部静脉溃疡）的人而言，它们是处方用品。但是压力袜同样也适合日常穿着。它们可用来帮助从事各类工作或娱乐活动的人们促进健康循环。需要长时间坐着或站着工作的人，连续数小时坐在汽车或飞机座椅上的旅行者，以及从事跑步运动的运动员，这些人都可以享受压力袜带来的好处。有些人认为压力袜实际上会阻断血液循环，因此很危险；然而，尺寸合适的压力袜并不会阻断血液循环。 | 穿着弹力袜能够在一定程度上减缓病情的发展，但无法治疗静脉曲张。为什么穿弹力袜无法治疗静脉曲张？静脉曲张的病因是长期静脉高压导致的下肢静脉功能不全，包括静脉壁松弛扩张和静脉瓣膜损伤。凡是能够增加下肢静水压的行为都是可以加速静脉曲张发生发展的。反之，能够减少下肢静水压的就能够减缓病情的发展。因此穿着弹力袜是静脉曲张保守治疗的主要方式。但下肢静脉功能不全，包括静脉壁松弛扩张和静脉瓣膜损伤是一个不可逆的过程。穿着弹力袜不能阻止这个过程的进展。如果发生了静脉曲张建议穿戴医用弹力袜预防快速进展，如果CEAP分级已经达到2-3级，就可以考虑微创手术了。 |
| 2019 | Chinese | 46 | May,2025 | 野白菊能预防偏头痛吗？ | https://www.cochrane.org/zh-hant/evidence/CD002286_feverfew-preventing-migraine | https://www.msdmanuals.cn/home/special-subjects/dietary-supplements-and-vitamins/feverfew | https://zhuanlan.zhihu.com/p/516120432 | 偏頭痛是一種常見且致殘的頭痛疾病。小白菊 (Tanacetum parthenium L.) 是作為一種用於預防偏頭痛發作的草藥。對於最新的 Cochrane 文獻回顧，我們回顧了截至 2015 年 1 月支持或反對小白菊預防偏頭痛的可用證據，並發現了六項研究，其中包括 561 名參與者。總體上，這些研究具異質性，他們的結果好壞參半。最新的文獻回顧顯示，與安慰劑相比，小白菊並未有明顯益處。我們在本次文獻回顧中加了一項規模更大且按高標準執行的新研究。結果表明，與安慰劑相比，小白菊每月偏頭痛的發作頻率減少了一半多一點 (0.6)。疼痛的嚴重程度或持續時間長短並無差異。這些結果來自一項中等規模的單一研究，因此必須審慎看待這些結果。在納入探討的研究中，沒有與小白菊相關的主要不良反應。 | 人们服用小白菊主要是为预防偏头痛和治疗炎症。它也被用来改善情绪并治疗哮喘、痛经、酒渣鼻和关节炎，据称具有抗癌特性。来自 6 项不同研究的证据显示小白菊预防偏头痛的效果各不相同，但是最近的一项研究规模更大，并且显示每月偏头痛数量呈小幅减少。研究结果的差异可能反映了所用小白菊的制剂和剂量不同。近期研究评估了小白菊与其他补充剂（如镁和辅酶Q10）的联合使用效果。 | 小白菊的主要活性成分是小白菊内酯（Parthenolide）、除虫菊酯（Pyrethrin），能阻止血小板聚集，并促进释放某些脑化学物质如血清素和炎症介质。上世纪70年代，英国国家煤炭委员会首席医务官的妻子患有严重的偏头痛，一个偶然的机会，当地百姓推荐她尝试小白菊，很快偏头痛几乎痊愈了。英国健康杂志报导了这位罹患长期性偏头痛的68岁老妇人，尝试使用小白菊的叶片，几个月後症状完全解除。因而自1980开始小白菊在英国广受欢迎。随後法国、加拿大开发为预防偏头痛、解除月经痉挛疼痛和治疗关节痛之植物性药剂。1990年代曾跃居美国植物性制药之销售冠军。 |
| 2019 | English | 3 | May,2025 | Can acupuncture be effective for people with epilepsy? | https://www.cochrane.org/evidence/CD005062_acupuncture-epilepsy | https://pmc.ncbi.nlm.nih.gov/articles/PMC4273587/ | https://pmc.ncbi.nlm.nih.gov/articles/PMC4273587/ | Acupuncture did not have excess adverse events compared to control treatment in the included trials. However, the included trials were small, heterogeneous and had a high risk of bias. It remains uncertain whether acupuncture is effective and safe for treating people with epilepsy. Compared with Chinese herbs, needle acupuncture plus Chinese herbs was not effective in achieving satisfactory seizure control (at least 50% reduction in seizure frequency). Compared with valproate, needle acupuncture plus valproate was not effective in achieving freedom from seizures or satisfactory seizure control. | The results of the analysis suggested that acupuncture combined with Western medicine is probably helpful in patients with epilepsy, but strong supportive data are not yet available. Given that this study is based on a low to moderate evidence-based analysis, the conclusions should be viewed with caution. | There was a reduction in the mean number of seizures per month in all patients during the follow-up period. Acupuncture significantly reduced the number of seizures in the intervention group in the final phase of the study. QOL improvements occurred in all patients, however, more significantly in the intervention group. |
| 2019 | English | 7 | May,2025 | Can aspirin improve the lives of people with vascular dementia? | https://pmc.ncbi.nlm.nih.gov/articles/PMC4171457/ | https://www.mdpi.com/2673-9879/3/1/11 | https://www.mdpi.com/2673-9879/3/1/11 | Low‐dose aspirin is frequently used as 'treatment as normal' in control groups and as a baseline treatment in pharmacological trials. There is still no good evidence that aspirin is effective in treating patients with a diagnosis of vascular dementia. There is increasing concern that low‐dose aspirin is associated with increased risk of haemorrhages. Further research is needed to assess the effect of aspirin on cognition, and on other outcomes such as haemorrhages, mortality, institutionalisation and behaviour. | We describe aspirin's effects on the primary prevention of cognitive impairment and various subtypes of dementia, as well as its role in cognitive decline in certain subsets of patients, including those with cerebral small vessel disease (CVSD), coronary heart disease (CHD), and gender differences. Overall, the benefits of aspirin in preventing dementia and cognitive decline remain inconclusive. The majority of cohort studies investigating aspirin's role in preventing cognitive decline or dementia looked promising, but this was not supported in most randomised controlled trials. However, aspirin may still be beneficial in certain subgroups of patients (such as CHD, VD, and CSVD) and warrants further investigation. | The broad dementia outcome occurred in a similar percentage of participants in the aspirin group and placebo group: 548 participants (7.1%) vs. 598 (7.8%), rate ratio 0.91 [95% confidence interval (CI), 0.81--1.02]. Thus, the CI excluded proportional hazards of >2% and proportional benefits of >19%. Aspirin does not have a large proportional effect on the risk of dementia. Trials or meta-analyses with larger total numbers of incident dementia cases to increase statistical power are needed to assess whether any modest proportional 10--15% benefits of 5--7 years of aspirin use on dementia exist. |
| 2019 | English | 17 | May,2025 | Can lumbar supports treat or prevent lower back pain? | https://pmc.ncbi.nlm.nih.gov/articles/PMC7046130/ | https://pubmed.ncbi.nlm.nih.gov/10908512/ | https://jamanetwork.com/journals/jama/fullarticle/187623 | There is moderate evidence that lumbar supports are not more effective than no intervention or training in preventing low‐back pain, and conflicting evidence whether they are effective supplements to other preventive interventions. It remains unclear whether lumbar supports are more effective than no or other interventions for treating low‐back pain. There is still a need for high quality randomised trials on the effectiveness of lumbar supports. One of the most essential issues to tackle in these future trials seems to be the realization of an adequate compliance. Special attention should be paid to different outcome measures, types of patients and types of lumbar support. | Five randomized and two nonrandomized controlled preventive trials and six randomized therapeutic trials were included in our review. Overall the methodological quality of the studies included in our review was rather low. Only four of the thirteen studies scored positive on 50% or more of the internal validity items. There was moderate evidence that for primary prevention lumbar supports are not more effective than other types of treatment or no intervention. No evidence was found on the effectiveness of lumbar supports for secondary prevention. The systematic review of therapeutic trials showed that there is limited evidence that lumbar supports are more effective than no treatment, while it is still unclear if lumbar supports are more effective than other interventions for treatment of low back pain. | Overall, lumbar supports or education did not lead to a reduction in low back pain incidence or sick leave. The results of the subgroup analysis need to be confirmed by future research. Based on our results, the use of education or lumbar supports cannot be recommended in the prevention of low back pain in industry. |
| 2019 | English | 20 | May,2025 | Can steroids be used as a treatment for spinal cord injury? | https://pmc.ncbi.nlm.nih.gov/articles/PMC9064751/ | https://www.sciencedirect.com/science/article/abs/pii/S1878875021003090 | https://www.neurology.org/doi/10.1212/WNL.0000000000009263 | In the treatment of patients with acute spinal cord injury (SCI), there is a lot of debate regarding surgical treatment strategies and pharmacological management, such as steroid use. In particular, the efficacy of steroid use, such as methylprednisolone sodium succinate, has been increasing and decreasing and is still intensely debated. The practice guidelines reported so far for this are also at the "suggest" stage with weak recommendations. Therefore, this review aims to summarize the effects of steroid use on SCI. This review provides an overview of current practical guidelines and clinical studies on steroid use in patients with SCI. | ACSI is a devastating and life-altering injury and, while numerous studies have been conducted on treatments, currently no consensus steroid therapy protocol exists. MP use in the setting of ASCI has been extensively studied and initially showed promise in both animal and human models. Adoption of high-dose MP protocols based on the results of NASCIS II and III has been followed by decreasing confidence in the validity of its results, particularly in the context of its association with adverse events. | The broad electronic search strategy yielded 1,574 articles, and of these, 16 studies were included in the final analysis (3 randomized controlled trials and 13 observational studies). The quality assessment revealed that the observational studies and the randomized controlled trials were of modest or low risk of bias. The random-effects meta-analysis did not detect a significant difference between the methylprednisolone and control groups for the pooled motor and sensory scores at last follow-up. This lack of difference was independent of study design and duration of follow-up. The corticosteroid was associated with a significantly higher incidence of adverse events, which included gastrointestinal hemorrhage and respiratory tract infection. |
| 2019 | English | 21 | May,2025 | Can acupuncture treat vascular dementia? | https://pmc.ncbi.nlm.nih.gov/articles/PMC5728254/ | https://www.sciencedirect.com/science/article/pii/S0965229922000590 | https://www.sciencedirect.com/science/article/pii/S1876382020314372 | Currently, there are no licensed treatments for VaD. Acupuncture has been used for years to treat VaD in China. However, the effectiveness of acupuncture for VaD remains controversial. There are so far two published systematic reviews referring to 'acupuncture' and 'vascular dementia'. A Cochrane systematic review published in 2007 and updated in 2011 aimed to assess the efficacy and adverse effects of acupuncture for treating VaD. However, this review did not include any clinical trials because the criteria for including studies were quite strict. To sum up, it remains unknown whether acupuncture is effective and safe for treating patients with VaD. | Acupuncture is probably helpful in vascular stroke, but strong supportive data are not yet available. Acupuncture should be used cautiously, owing to the analysis of this study based on low to moderate evidence. Further high-quality, large-scale RCTs should be conducted. | Acupuncture showed potential therapeutic effects for patients with dementia, but the quality of the evidence was not high. Higher-quality RCTs are warranted to confirm the clinical effects of acupuncture in the treatment of dementia. |
| 2019 | English | 43 | May,2025 | Are compression stockings helpful for varicose veins? | https://prod.emoryhealthcare.org/stories/heart-health/will-compression-hose-help-my-varicose-veins | https://www.yalemedicine.org/news/blood-clots-varicose-veins-sore-legs-compression-socks-help | https://www.ncbi.nlm.nih.gov/books/NBK328015/ | Compression hose exert external pressure on the legs to reduce the pooling of blood in varicose veins and improve the vein circulation. These hose often improve the symptoms of varicose veins and may minimize your risk of developing new varicose veins. | But choosing the right compression socks—or stockings (the two terms are sometimes used interchangeably)—can be complicated. First, you need to get a pair that suits your situation. For instance, if you have a medical condition, you may need a prescription and a particular compression strength (low, medium, or high). Compression stockings won't cure these conditions, but whether you have a serious medical issue or not, they can help manage symptoms. | Graduated compression hosiery is widely used as first line treatment for varicose veins. Compression stockings work by compressing the superficial veins to keep them collapsed and empty of blood and thereby pushing more blood into the deep venous system. This results in a reduction of venous pressure in the leg and subsequently a decrease in leg swelling. The compression is graduated, exerting an external pressure which is higher at the ankle (minimum 14mmHg) than the calf and thigh, thus increasing blood velocity within the deep venous system. It is recognised that the amount of pressure required is dependent on the severity of the condition. |
| 2019 | English | 46 | May,2025 | Can feverfew prevent migraine headaches? | https://pmc.ncbi.nlm.nih.gov/articles/PMC7133498/ | https://americanheadachesociety.org/news/incorporating-nutraceuticals-for-migraine-prevention | https://www.healthline.com/nutrition/feverfew | The most recent trial added to this version of the review is rigorous and larger (n = 218), using a stable feverfew extract at a dose determined by a previous dose‐finding trial. It reports that feverfew reduced migraine frequency by 1.9 attacks from 4.8 to 2.9 and placebo by 1.3 from 4.8 to 3.5 per month, resulting in a difference in effect between feverfew and placebo of 0.6 attacks per month. For the secondary outcome measures no statistically significant differences were reported. This constitutes low quality evidence, which needs to be confirmed in larger rigorous trials. It appears from the data reviewed that feverfew is not associated with any major safety concerns. | Feverfew leaves contain parthenolide and other ingredients that can inhibit the release of serotonin and prostaglandin. Serotonin and prostaglandin are naturally occurring substances that dilate blood vessels and can trigger migraine. Studies have shown that feverfew may help reduce the frequency and duration of migraine attacks. It can also help reduce symptoms of migraine, such as pain, nausea, vomiting, and light or noise sensitivity. | For centuries, people have been taking feverfew to treat migraine. In test-tube studies, compounds in feverfew, such as parthenolide and tanetin, helped stop the production of prostaglandins, which are molecules that promote inflammation. However, human studies on feverfew and migraine show mixed results. In a review of six studies involving 561 people, four studies found that feverfew helped reduce the frequency and intensity of migraine attacks, while two studies found no effect. Based on current research, feverfew appears to be only slightly effective against migraine attacks. More human studies are needed to draw solid conclusions. |
| 2021 | Chinese | 1 | May,2025 | 戴踝关节护具有助于跟腱炎的恢复吗？ | https://orthopt.org/uploads/content_files/files/8%20%20Chinese_Ankle%20Sprain%20-%202014%2007%2027%20.pdf | https://paper.people.com.cn/xaq/html/2014-05/01/content_1445567.htm | https://www.familydoctor.cn/hlthsci/genjian-tengtong-dayi-jieduan-fangan-yingdui-jianqiaoyan-665612.html | 临床师应建议急性外踝扭伤的患者于患侧使用外部支撑并且渐进性负重。外支撑的类型和步态辅助器具应基于患者损伤程度、组织修复的阶段、保护控制所需程度、疼痛程度和病人喜好来确定。对比较严重的损伤，指导原则是：固定范围从半刚性支具过渡到膝以下固定。（基于强证据建议） | 腱鞘炎：急性反应期（0~72小时）：先"稳住"——用可调节的踝关节支具固定在不偏不歪的位置，避免跟腱再受牵拉；每次冰敷不超过15分钟，隔2小时敷一次，减轻肿胀；用弹性绷带缠出10~20毫米汞柱的压力（类似轻压的感觉），防止肿得更厉害。 | 跟腱位于足跟与小腿之间，长15厘米左右，也是人体中最粗壮的一条肌腱，我们日常的跑、跳、行走等活动都依赖着这条肌腱，由于承载着身体巨大的负重量，可以说它也是人体中最劳累的肌腱。所以说，别看它够表面上够强壮，但在我们做一些剧烈运动、过度运动或者是运动前没有进行充分热身的时候，都会对其造成一定的损伤，轻者会造成跟腱炎症，严重的话就会直接导致肌腱断裂。因此，甭管我们是否患上了"亚腱康"，建议您在运动时最好带上护踝等保护性护具，这对保护跟腱有很好的辅助作用。 |
| 2021 | Chinese | 2 | May,2025 | 温水海绵浴是降低儿童发热的好方法吗？ | http://health.people.com.cn/n1/2018/1023/c14739-30358110.html | https://www.medlinker.com/m/share/question/30105991 | https://www.mountelizabeth.com.sg/zh/health-plus/article/sick-child-what-to-do | 所以，无论是在体温上升、高热持续，还是退烧阶段，都没有必要温水擦浴，这也应该是所有的权威医学机构都首选退热药退烧，而不是温水擦浴的原因，甚至好几家都明确反对温水擦浴，《美国儿科学会育儿百科》第六版也删除了温水擦浴的内容，中国最新的发热指南也明确建议不要温水擦浴退热了。 | 秋冬之际是一个感冒高发季，孩子发烧使许多家长们谈"热"色变。那孩子出现发高烧，家长该如何给孩子降温？太原市妇幼保健院急诊科护士樊蓉表示，最简单、最有效，也是最安全的办法，就是给孩子温水擦浴。温水擦浴孩子的皮肤，可使身体表面血管扩张，促进血流加速，改善血液循环，减轻组织缺氧，增强新陈代谢。擦拭的部位主要有额头、腋窝、肘窝、腹股沟处，这些部位因为有大血管，所以在擦拭时可以多停留一会儿，以促进降温。如果体温保持在38.5℃以上，需要及时就医。 | 如果孩子发热，您可以通过降低体温来帮助孩子感觉更舒适。为此，给孩子穿浅色衣服。避免用厚厚的毯子裹住孩子，让孩子的房间保持凉爽。给孩子洗温水海绵浴。不要用冷水或冰水，避免每次给孩子用海绵擦浴超过30分钟。洗完澡后立即擦干也很重要，以免孩子着凉。如果您的婴儿小于3个月且发热超过38°C，请联系您的儿科医生。医生可能会给您开扑热息痛婴儿滴剂，每隔4到6小时滴一次。请确保根据孩子的体重给他正确的剂量。 |
| 2021 | Chinese | 3 | May,2025 | 叶酸有助于改善认知和治疗痴呆症吗？ | https://www.pumch.cn/detail/12849.html | https://www.familydoctor.cn/news/yesuan-sheru-buzu-danao-hui-fasheng-shenme-bianhua-285936.html | https://www.163.com/dy/article/JROUA1A90536BTG3.html | 叶酸在人体内的作用远不止"孕妇专属"。它参与同型半胱氨酸（Homocysteine）的代谢，这是一种与心血管疾病和神经退行性疾病相关的氨基酸。当叶酸水平不足时，血液中的同型半胱氨酸会升高，导致氧化应激和血管损伤，进而影响大脑健康。研究发现，高同型半胱氨酸水平与认知下降、轻度认知障碍（MCI）以及老年痴呆（如阿尔茨海默病）的风险显著相关。叶酸缺乏的老年人（血清叶酸<10 nmol/L）比叶酸水平正常者更容易出现记忆力减退和执行功能障碍。此外，叶酸缺乏还可能加剧脑萎缩，尤其是在海马区——负责记忆的关键区域。但反过来，如果叶酸水平正常，额外补充叶酸能预防痴呆吗？答案并非绝对。研究显示，对于叶酸充足的人群，额外补充并不会显著降低痴呆风险。 | 叶酸与B族维生素。阿尔茨海默病发生还可能与B族维生素、叶酸缺乏有关。最近，由Goodwin等进行的一项早期研究发现，营养摄入与认知功能之间存在相关关系，与对照组相比，有更多的阿尔茨海默病发生，并且患者会出现血清同型半胱氨酸水平升高。而叶酸与维生素B12能降低体内高半胱氨酸含量，故补充叶酸及维生素B12有助于防止老年性痴呆症的发生。 | 低叶酸水平同样影响阿尔茨海默病和痴呆症的患病风险。"正常情况下，叶酸与维生素B6、B12协同作用，将蛋白质代谢副产物同型半胱氨酸转化为具有抗氧化特性的有益化合物，"穆恩解释道。但当身体无法获取足量叶酸或无法有效利用时，同型半胱氨酸水平往往持续累积。"必须严格控制同型半胱氨酸水平，因其升高不仅关联心血管疾病风险增加，而心血管疾病本身又是阿尔茨海默病和血管性痴呆的重要诱因。" |
| 2021 | Chinese | 4 | May,2025 | 用胶带可以去除疣吗？ | https://www.cochrane.org/zh-hans/evidence/CD001781_topical-treatments-skin-warts | https://www.mayoclinic.org/zh-hans/diseases-conditions/plantar-warts/diagnosis-treatment/drc-20352697 | https://zh.wikihow.com/用管道胶带除疣 | 病毒疣是一种常见的皮肤疾病，它由人乳头瘤病毒引起，好发于手部或足部。尽管疣没有危害性而且通常不需要任何治疗就会及时消失，但它们会影响美观并伴随疼痛。长在脚上的疣也被称作"足底疣"或"疣"。在本综述最新版的制作过程中，管道胶带因其安全简单且易于使用获得了青睐；然而，该试验的规模相对较小。在这项更新的综述中，我们发现了两项关于管道胶带的进一步试验，表明这种治疗方法不如最初想象的那么有效。 | 使用布基胶带去疣是一种无害但未经证实的方法。这种方法是将银色布基胶带贴在疣上，隔几天换一次胶带。每次换胶带前，先浸泡患处，再用浮石或砂锉轻轻去除表面已经坏死的组织。然后让疣暴露在空气中几个小时，待晾干后再贴上胶带。 | 疣是由人类乳头瘤病毒引起的生长物，它们既难看，又难以消除！治疗疣最著名的家庭疗法是使用管道胶带。有一种疗法被称为胶带封闭疗法（DTOT），使用这种疗法时，你需要用管道胶带长时间遮盖疣体，再用粗糙的物体擦掉疣体上的死皮。然后，等待几小时，让疣体暴露在空气中，之后再重新贴上新的管道胶带。你必须重复这一过程，直到疣体完全消失，可能需要长达2个月的时间。这种方法并非对所有疣都有效，但是它安全，并且花费很少，值得一试。 |
| 2021 | Chinese | 6 | May,2025 | 维生素 B12 和日晒结合可以治疗白癜风吗？ | https://www.mayoclinic.org/zh-hans/diseases-conditions/vitiligo/diagnosis-treatment/drc-20355916 | https://weixin.91160.com/h5/wecontent/doctorsaid/detail.html?id=583427 | https://www.sohu.com/a/484763324_121148905 | 有限的几项研究表明，银杏这种草药能够让白癜风患者的肤色复原。其他小型研究表明，α硫辛酸、叶酸、维生素C和维生素B-12加上光照治疗可以帮助部分患者恢复肤色。和任何非处方治疗方法一样，在尝试替代疗法之前，请咨询医务人员，以确保它们不会干扰您正在接受的其他治疗方法。 | 白癜风由于机理不明，治疗上比较棘手，联合用药治疗往往有不错的疗效。1）光化学疗法+糖皮质激素，如8－甲氧补骨脂素外涂加强的松口服。2）免疫调节剂+糖皮质激素，如转移因子胶囊加强的松口服。3）叶酸和维生素B12联合日光照射治疗：有研究表明白癜风患者血液中叶酸和维生素B12的水平往往低于正常，通过口服叶酸和维生素B12并局部日光或UVB照射治疗白癜风有良效，但具体作用机制尚不清楚。4）手术治疗+光化学疗法,如自体表皮移植加8－甲氧补骨脂素外涂，UVB照射。 | 有很多白癜风患者自行补充维生素的例子，一些研究调查表明，白癜风患者体内叶酸、维生素B12的水平比正常人低一些。所以，补充叶酸和维生素B12可帮助这些人恢复正常的肤色。还有建议配合日晒，3～6个月后，有相当数量的色素区产生。 |
| 2021 | Chinese | 9 | May,2025 | 硒有助于预防癌症吗？ | https://www.cochrane.org/zh-hans/evidence/CD005195_selenium-preventing-cancer | http://imohw.tmu.edu.tw/idohtmu/wp-content/uploads/2018/08/补充微量元素-硒Selenium-真的可以预防癌症吗.pdf | https://m.thepaper.cn/newsDetail_forward_23866245 | 精心地设计和开展的随机对照试验表明，硒补充剂在降低癌症风险方面没有任何益处（高级别质量证据）。一些随机对照研究报告，在补充硒的受试者中，高级别前列腺癌和2型糖尿病的发病率更高。在这些研究中，尚无明确证据表明受试者的硒基线水平对结局有影响。观察性纵向研究表明硒暴露与某些癌症类型的风险成反比关系，但也有报道称其为零和直接关系，并且没有系统模式表明剂量-反应关系。总体而言，没有证据表明通过饮食或补充营养来增加硒的摄入量可以预防人类的癌症。 | 經過不同醫學資料庫搜尋後，我們選擇由 Vinceti M 等人於 2018 年發表在 Cochrane Database Syst Rev 的系統性文獻回顧，該文獻納入現有最多的高品質隨機對照試驗（10 篇，共有 27,232 位成年受試者），比較每日服用 200-400 微克的硒補充品或服用安慰劑對於預防癌症的療效與安全性差異。結果顯示，平均使用 2-15 年後，接受硒補充的受試者並未有顯著較低的整體癌症發生風險（risk ratio：1.01, 95%CI：0.93-1.10）。該文獻同時針對大腸直腸癌、非黑色素瘤、肺癌、乳癌、膀胱癌和前列腺癌進行個別分析後，也均無法證實補充硒有顯著的防癌療效。儘管硒可能有預防癌症發生的可能藥理機轉，但現有系統性回顧整理相關人體臨床試驗的結果卻顯示額外補充硒並無法顯著減少癌症發生風險。 | 那这样是不是就能说明补硒，可以防癌抗癌呢？答案是不能！不可否认的是，在人体大量缺乏硒元素时，身体会生病，其中又以克山病最为典型。但这并不意味着我们可以盲目补硒，一项涉及多项补硒与癌症预防的汇总研究《硒和癌症预防》中指出，没有发现任何证据支持补硒可帮助人类预防癌症。 |
| 2021 | Chinese | 10 | May,2025 | 服用避孕药可以治疗卵巢囊肿吗？ | https://www.mayoclinic.org/zh-hans/diseases-conditions/ovarian-cysts/diagnosis-treatment/drc-20353411 | https://y.dxy.cn/hospital/879/851771.html | https://www.qingdaonews.com/htm7/fuke/lcnz/yf/6420.html | 治疗方法取决于您的年龄、囊肿的类型和大小，还取决于您的症状。医务人员可能会建议：药物。激素类避孕药（如避孕药）会阻碍排卵，这可能防止出现更多卵巢囊肿。但避孕药无法缩小现有的囊肿。 | 卵巢囊肿为促排卵的禁忌症之一，而且卵巢囊肿的存在会影响妊娠。因此在促排卵前一定要消除卵巢囊肿。而避孕药可以抑制促卵泡激素和促黄体生成素分泌，服药期间卵巢功能被抑制、从而降低了卵巢功能性囊肿的发生，也可使原有的卵巢功能性囊肿缩小、消失。 | 在专家介绍中我们得知，避孕药是可以预防卵巢囊肿的。口服避孕药本身就是激素类药物，能抑制脑垂体激素分泌，那些原来促进卵巢生成卵泡、排卵的激素受到抑制后，卵巢便不再排卵，女性也就不会怀孕。同样道理，没有了卵泡发育、排卵的过程，功能性卵巢囊肿也就不会发生，所以，口服避孕药本身就是预防这些囊肿的方法。对于已出现的功能性卵巢囊肿，服用避孕药后，卵巢处于"消停"状态，囊肿也会消退得更快。卵巢巧克力囊肿一般需要手术治疗，但由于激素原因仍在，术后常易复发，一般都需要口服避孕药中的高效孕激素，以抑制异位内膜的生长。避孕药可以预防卵巢囊肿，但是却不建议发现囊肿的女性随便服用。因为避孕药本身费用过多，也会引起体内激素紊乱，带来不好的问题。 |
| 2021 | Chinese | 12 | May,2025 | 晚安油可以治疗湿疹吗？ | https://karethic.com/zh-CN/治疗湿疹的四种天然方法/ | https://fenjinbio.com/薰衣草油湿疹缓解/ | https://www.xzmedtech.com/show-367396.html | 湿疹是一种常见的皮肤病，症状表现为皮肤红肿、瘙痒、干燥甚至脱皮。对于许多人来说，湿疹不仅仅是对皮肤的困扰，更是生活质量的重大影响。近年来，越来越多的研究和家庭疗法发现，香油作为一种天然护肤品，搭配其他成分可以有效缓解湿疹症状。 | 薰衣草油是一种有效的天然疗法，适用于轻至中度湿疹患者。通过适当的稀释和使用方法，薰衣草油不仅可以缓解湿疹症状，还能促进皮肤愈合和修复。然而，在使用过程中应注意个体差异和潜在的过敏反应，必要时应咨询专业医生或芳疗师的建议。 | 有许多自然疗法可以帮助缓解湿疹症状。天然疗法是乳木果油。它富含必需脂肪酸，具有抗炎和抗菌特性，只要它是原始的、未精制的，当然还有经过认证的有机乳木果油，您可以通过它的黄色识别出来。例如，在皮肤上涂抹乳木果油可以帮助深层滋养和保湿，同时减少瘙痒和炎症。应特别注意日常皮肤护理。使用温和、无香味的产品和富含油脂的保湿霜有助于保持皮肤屏障的完整。还建议避免热水淋浴和长时间淋浴，因为这会使皮肤干燥。 |
| 2021 | Chinese | 14 | May,2025 | 涂抹维生素 E 软膏可以去除皮肤疤痕吗？ | https://zhishi.scarbbs.com/bahenxiufu/bahenzixun/10683.html | https://smmip.club.xywy.com/tt/194541931.htm | https://moonandskin.com/zh/blogs/glow-up/how-to-use-vitamin-e-for-scars | 维生素E软膏，是由维生素E作为主要成分制成的一种质地柔软的护肤品。它具有温和而有效的修复功能，可以滋养皮肤、深层滋润、改善肌肤质地。而在祛除疤痕方面，维生素E软膏也发挥出了其独特的功效。维生素E软膏的祛除疤痕功能主要是通过以下几个方面实现的：润肤修复：维生素E具有良好的保湿性，能够深层滋润皮肤，帮助恢复皮肤天然屏障功能，加速伤口愈合，减少疤痕形成。抗氧化：维生素E具有强大的抗氧化作用，可以清除自由基，减少炎症反应，加快血液循环，促进色素沉着的疤痕淡化。促进新陈代谢：维生素E可以促进血液循环，提高皮肤细胞的新陈代谢速度，加速疤痕的脱落和组织再生。 | 维生素e软膏通常可以辅助去疤痕，也可以通过微针疗法进行治疗。维生素e软膏中含有大量的维生素E成分，有抗氧化的作用，还可以在皮肤表面形成保护膜，另外也有助于淡化局部的色素，对于表浅疤痕可以起到辅助改善的效果，但是也需要在医生的指导下用药。在去除疤痕的时候，也可以选择微针疗法，主要是利用滚轮上的小针头刺破皮肤，导入一些去疤痕类的药物，能够直接作用于疤痕组织，还可以促进疤痕纤维组织恢复，达到消除疤痕的目的。 | 维生素E被认为在维持皮肤健康方面发挥多种作用：抗氧化保护：它帮助中和自由基——这些不稳定的分子会损害细胞并促进衰老和皮肤疾病。保湿：维生素E可能增强皮肤水分，这对于保持弹性和减少疤痕形成的可能性至关重要。伤口愈合：一些研究表明，维生素E可能通过调节炎症和促进胶原蛋白生成来支持愈合过程，尽管证据不一。 |
| 2021 | Chinese | 17 | May,2025 | 发酵乳制品有助于降低高血压吗？ | https://e.dxy.cn/wisdom/front/zhihuihao/2953 | https://www.medsci.cn/article/show_article.do?id=28a9194e9124 | https://ecoapp.qianzhan.com/detials/210323-60643315.html | 最新的一项荟萃分析结果表明：存在于发酵乳制品中的益生菌，有助于控制血压水平。研究人员调查发现，摄入适量的益生菌可以把收缩压和舒张压分别降低 3.5 和 2.4 mmHg 左右，若是某些人的血压水平过高或者摄入多种益生菌，那么血压会降得更多。这项研究论文发表在 Hypertension 杂志上。 | 一项大型国际前瞻性城乡流行病学(PURE)研究有关乳制品摄入量与代谢综合征的患病率之间的关系，此项结果发现每天至少吃两份乳制品会为人体带来良好的代谢作用，能够降低患糖尿病、高血压和心血管疾病的风险。此次研究结果表明全脂乳制品和发酵乳制品是是此次研究中降低风险率表现得最强的。该研究结果在5月18日发表在《BMJ Open Diabetes Research & Care》上。 | 研究人员表示，"新的证据表明，抗高血压发酵乳，包括益生菌、生物活性肽和从用特定乳酸菌发酵的乳中获得的胞外多糖，可能会调节肠道微生物群。因此，开发具有肠道微生物调节和降血压作用的特制发酵乳是有潜力的。" |
| 2021 | Chinese | 22 | May,2025 | 萘普生能缓解偏头痛吗？ | https://www.cochrane.org/zh-hans/evidence/CD009455_naproxen-acute-migraine-adults | https://www.nmpa.gov.cn/wwwroot/otc2h/x053.htm | https://www.mayoclinic.org/zh-hans/diseases-conditions/chronic-daily-headaches/in-depth/headaches/art-20046729 | 非甾体类抗炎药（non-steroidal anti-inflammatory drugs, NSAIDs）常用于治疗偏头痛。有一种NSAID是萘普生。2013年5月22日，我们检索了使用萘普生治疗偏头痛的临床试验。我们发现了6项高质量研究，涉及约2700人。与安慰剂相比，萘普生能有效缓解成人偏头痛，但效果较弱。中度或重度头痛时，10个服用萘普生的患者中，约有2人可在2小时内疼痛消失（17%）。但使用安慰剂时，10人中约1人（8%）可在2小时内疼痛消失。萘普生可减轻10人中5人的头痛症状，而安慰剂则是10人中的3人。萘普生并不如布洛芬或舒马曲坦等其他药物好。在我们发现的研究中，500mg或825mg剂量的萘普生都不是偏头痛的最佳用药。 | 本品为非甾体类抗炎镇痛药，可抑制前列腺素的合成而发挥抗炎镇痛作用。用于缓解轻度至中度疼痛，如关节痛，神经痛，肌肉痛，偏头痛，头痛，痛经。 | 头痛非处方类止痛药。医务人员可能会建议您使用非甾体抗炎药（NSAID）。此类药物包括萘普生钠（Aleve）和布洛芬（Advil、Motrin IB 等）。这些药物可以在开始头痛后立刻缓解疼痛。 |
| 2021 | Chinese | 24 | May,2025 | 鲨鱼软骨有助于缓解骨关节炎的疼痛吗？ | https://www.cochrane.org/zh-hant/evidence/CD005614_chondroitin-osteoarthritis | https://zhuanlan.zhihu.com/p/356089672 | https://www.epochtimes.com/gb/22/10/28/n13854515.htm | 我們執行了一篇探討軟骨素用於骨關節炎患者效益的文獻回顧。我們搜尋2013年11月前的研究，共找到包含9,110位患者的43個試驗。大部分為針對膝部骨關節炎的研究。軟骨素可能可以在短期內（少於6個月）些微地改善疼痛。軟骨素改善了稍多人們20%的膝部疼痛。根據Lequesne's index（結合測量疼痛、功能與失能狀況），軟骨素可能些微提升了生活品質。相較於其他用劑，軟骨素對於不良事件與嚴重不良事件只有些許或沒有差異。軟骨素讓受影響的關節，在X光下關節間隙窄化的狀況稍微減緩。在某些研究方法品質比較好的研究中，軟骨素顯示並沒有改善疼痛與身體功能。 | 无论是人医还是兽医，硫酸软骨素有没有用这个话题也是充满了争议，因为既没有足够的证据证明它有用，同时又没有足够的证据证明它们没用。 | 科学证明单独应用硫酸软骨素时是没有好处的。不过，有证据表明，硫酸软骨素与氨基葡萄糖联合使用可以减轻关节疼痛，改善关节活动度。允许减少常规抗炎药的剂量。不过，2006年的氨基葡萄糖/硫酸软骨素关节炎干预试验(GAIT)是一项对氨基葡萄糖、硫酸软骨素和两者联合治疗膝骨关节炎的大型安慰剂对照研究。它们单独给药或联合给药均不能显著降低疼痛。专家们对软骨素的疗效存在分歧，美国风湿病学会建议不要将其用于治疗髋关节或膝关节骨关节炎，仅对其用于治疗手部骨关节炎提出条件性的建议。相比之下，欧洲骨关节炎临床与经济学会建议使用药用级软骨素治疗手部骨关节炎。 |
| 2021 | Chinese | 27 | May,2025 | 香薰按摩有助于管理类风湿关节炎吗？ | https://www.xzmedtech.com/show-557483.html | https://www.xzmedtech.com/show-566989.html | https://www.ebiotrade.com/newsf/2025-3/20250323055207257.htm | 薰衣草凭借其抗炎、镇痛和放松的特性，为关节炎患者提供了一种有效的自然疗法。无论是通过香薰、按摩还是泡澡，薰衣草都能帮助减轻关节的不适，改善生活质量。然而，每个人的体质不同，因此在使用任何天然疗法前，最好咨询专业人士的意见。随着对自然疗法愈来愈多的关注，薰衣草无疑是探索关节炎缓解方法中的一个值得尝试的选择。 | 风湿关节炎虽然是一种令人苦恼的疾病，但精油提供了一种温和而有效的缓解方式。通过结合使用不同的精油，患者可以制定个性化的护理方案，帮助减轻疼痛，改善生活质量。在寻求精油疗法时，结合专业意见是关键，以确保安全有效。希望这篇文章能够为风湿关节炎患者带来新的希望与启发！ | 按摩疗法作为类风湿关节炎（RA）辅助干预的作用。类风湿关节炎（Rheumatoid arthritis，RA）是一种慢性自身免疫性疾病，主要特征为关节炎症、疼痛以及活动能力下降。在当前的医疗体系中，改善病情抗风湿药（DMARDs）和生物制剂等药物疗法虽然在一定程度上改善了RA的疾病转归，但许多患者仍会残留一些症状，严重影响生活质量。 |
| 2021 | Chinese | 28 | May,2025 | 淋浴蒸汽有助于治疗喉炎吗？ | https://www.mayoclinic.org/zh-hans/diseases-conditions/laryngitis/diagnosis-treatment/drc-20374267 | https://m.dxy.com/disease/25391/detail | http://health.people.com.cn/n1/2018/1120/c14739-30410282.html | 某些自我护理方法和家庭护理可能会缓解喉炎的症状并减轻嗓子的压力：呼吸潮湿的空气。使用加湿器使家里或办公室的空气保持湿润。通过喝热水或热水淋浴吸入蒸汽。请尽可量少使用嗓子。不要大声或长时间说话或唱歌。如果您需要在大群人面前讲话，请尝试使用麦克风或扩音器。多喝水以防止脱水（避免饮酒和喝咖啡）。润喉。尝试吮吸锭剂、用盐水漱口或嚼一块口香糖。避免使用减充血药。这些药物会使您的咽喉变干。避免压低声音说话。与普通说话相比，这会给您的嗓子造成更大的压力。 | 失声应该积极治疗原发性疾病，对于明确病因后的不严重情况，以下方法会有些帮助：为声带做的最好的事情是让它们休息一下。一两天不说话是最佳的选择。如果必须要讲话，可以用平稳的音调进行。这个方法之所以能起作用，是因为咽喉轻度的炎症有时候仅依靠休息就能恢复，一旦炎症缓解了，声带不再肿胀，语音就能恢复正常。过度使用声带会阻碍炎症的愈合过程。窃窃私语要比正常讲话更损害声带。热水淋浴产生的蒸汽有助于滋润声带并舒缓喉咙的疾病不适。保持室内湿度40%～60%，对咽喉声带的恢复也有影响。 | 缓解身体疼痛的止痛药也可缓解喉咙痛。在治疗喉咙痛的自然疗法中，可以尝试加蜂蜜的热茶，使用加湿器，或用盐水漱口。无糖药物滴落或使用含药物的咽喉喷雾剂也有益处。 |
| 2021 | Chinese | 36 | May,2025 | 吃枣有助于管理缺铁性贫血吗？ | https://www.cn-healthcare.com/articlewm/20201223/content-1174626.html | http://www.z2imc.com/news/252.html | https://www.yantai.gov.cn/art/2022/9/24/art_69837_3059251.html | 吃红枣可以补铁补血吗？其实不能。一方面红枣当中所含的铁元素并不多，每百克鲜枣中含有的铁元素仅在2毫克左右；干枣中仅能达到每百克1.2毫克。另一方面红枣中所含的铁元素并不利于人体的吸收，吸收效果很差。很多人盲目食用红枣用于补铁补血，甚至会大量摄入红枣，然而红枣的外皮很坚硬，大量摄入很容易刺激和损伤食道黏膜和胃肠黏膜，存在一定的风险，不建议为之。 | 红枣、红糖不补血！值得一提的是，很多女性担心来月经期间失血过多造成贫血，会习惯吃红枣、喝红糖水来补血。实际上，红枣和红糖中铁含量很低，不能改善缺铁性贫血。 | 每100克新鲜大枣（以下简称鲜枣）可食用部分铁含量为1.2毫克，干的红枣（简称干枣）为2.3毫克，但比起动物食材，铁含量则低得多。而且，我们每次吃红枣的量不会很大。按平均每天3-5颗大枣为例，进食鲜枣（无核）50克，或干枣（无核）25克，摄取的铁仅仅为0.6毫克左右。这与一日推荐摄入量——成年人每日需要的铁，男为12毫克、女为20毫克，相差甚远。且我们身体对不同的铁，吸收率是不相同的；动物性食物中的铁为血红素铁，吸收率高，为10%-20%以上；红枣属于植物性食物，吸收率只有3%-5%。所以，光指望吃红枣来补血，你每天吃一箩筐恐怕也难以达到好的效果。 |
| 2021 | Chinese | 43 | May,2025 | 泰诺能管理骨关节炎的症状吗？ | https://www.mayoclinic.org/zh-hans/diseases-conditions/arthritis/in-depth/arthritis/art-20046440 | https://www.xzmedtech.com/show-569647.html | https://www.mayoclinic.org/zh-hans/diseases-conditions/back-pain/in-depth/chronic-pain-medication-decisions/art-20360371 | 很多药物可用于缓解关节炎疼痛。大多数药物相对安全，但不存在完全没有任何副作用的药物。请与医疗护理专业人员沟通，针对您的具体疼痛症状制定疼痛管理计划。疼痛管理计划旨在减轻疼痛。同时缓解压力、提高活动能力和改善生活质量。无需处方即可购买的止痛药可以帮助缓解。这类药物包括对乙酰氨基酚（Tylenol 等）、布洛芬（Advil，Mothrin IB 等）和萘普生钠（Aleve）。 | 对乙酰氨基酚（如泰诺）是一种非处方药，在缓解轻至中度骨关节炎疼痛方面也非常有效。其副作用较少，适用于对NSAIDs过敏或不适合使用的患者。然而，使用时需要遵循剂量，以避免对肝脏的潜在损害。非甾体抗炎药如布洛芬、萘普生等是缓解骨关节炎疼痛的主要药物之一。这些药物通过抑制体内的炎症反应来减轻疼痛和肿胀。NSAIDs可以口服或外用，外用药物通常适用于轻度疼痛的患者，且副作用相对较小。 | 通常推荐对乙酰氨基酚作为轻度至中度疼痛的首选治疗药物。该药物可用于治疗皮肤损伤引起的疼痛、头痛或累及肌肉和骨骼的疾病。对乙酰氨基酚通常用作治疗骨关节炎和背痛的处方药。它也可能会与阿片类药物联合使用，以降低对阿片类药物的需求量。对乙酰氨基酚被认为可阻断中枢神经系统中的前列腺素的生成。与NSAID不同，对乙酰氨基酚不针对损伤部位的发炎，仅针对疼痛。一般认为对乙酰氨基酚比其他止痛药更安全，不会引起胃疼、出血等副作用。然而，长时间服用超过推荐剂量的药物，或者在服用对乙酰氨基酚的同时饮酒，会增加肾脏损伤和肝功能衰竭的风险。对乙酰氨基酚在治疗与骨关节炎有关的膝关节和髋关节疼痛方面不如NSAID有效。 |
| 2021 | Chinese | 45 | May,2025 | 欧米伽 -3 能治疗女性边缘型人格障碍吗？ | https://www.leepsyclinic.com/2016/10/blog-post_8.html | https://zhuanlan.zhihu.com/p/634148908 | https://www.hkbiotek.com/post/omega-3脂肪酸精神健康 | Omega-3不飽和脂肪酸常見於深海魚如三文魚和鯖魚之中，當中最有益的是EPA和DHA。可是，我們的身體無法自行製造這兩種脂肪酸。所以我們必須從食物中攝取，魚油產品正是很好的來源。缺乏EPA和DHA有機會增加患上精神疾病的風險。到底EPA和DHA如何影響精神健康？根據哈弗健康報告，有證據表明omega-3可以穿過腦細胞的細胞膜，與影響情緒的分子如血清素及其受器相互作用。亦有研究指明omega-3的抗炎特性有助穩定情緒病患者的情緒，對正在服用抗抑鬱藥的患者尤其有效。相比起DHA，EPA或更有效控制情緒病。EPA也有機會可以成為治療患有邊緣性人格障礙的方針。不過，還需要有更多證據支持omega-3有效治療情緒病這個論點。 | 在边缘型人格障碍（BPD）患者的小规模研究中，发现在1周内每天补充8克添加EPA和DHA的omega-3脂肪酸可有效减少攻击性和抑郁症状。这项研究的研究人员认为，EPA可能是中度BPD病例的有效选择。 | 從症狀上來看：對於情感失調症狀，可選用Topiramate、Lamotrigine、Divalproex sodium、Haloperidol、Aripiprazole、Olanzapine、Quetiapine、Omega-3脂肪酸、和Amitriptyline；衝動行為可受到Topiramate、Lamotrigine、Aripiprazole、和Omega-3脂肪酸的控制；精神症狀可靠Aripiprazole、Olanzapine、和Quetiapine獲得緩解。而部份研究指出Aripiprazole、Divalproex sodium、和Topiramate可改善人際問題；Omega-3脂肪酸亦可減少自殺傾向。 |
| 2021 | Chinese | 50 | May,2025 | 服用抗氧化剂补充剂能治疗生育问题吗？ | https://www.cochrane.org/zh-hans/evidence/CD007411_antioxidants-male-subfertility | https://www.cochranelibrary.com/es/cdsr/doi/10.1002/14651858.CD007807.pub3/full/zh_HANS | https://www.medsci.cn/article/show_article.do?id=0dbd902e3684 | 前往生育诊所就诊的夫妇中，男方服用抗氧化剂，将提升活产率。但基于七项小型随机对照试验的证据，其总体质量偏低。低质量证据同样提示临床妊娠率可能增加。总而言之，目前尚未有证据表明会增加流产，但非常低质量的证据提示抗氧化剂将可能更易带来轻度的胃肠道不适。总之，应告知低生育伴侣，目前的证据尚无定论，主要在于目前的研究中试验的方法报告不足、没有充分报告活产率及临床妊娠率。由于事件发生率低、中途脱落数量大、每组受试者数量少，使得目前汇总的结果更加不精确。为确定抗氧化剂的作用，还需进行规模更大、设计优良的安慰剂对照的随机对照临床试验，并充分报告妊娠率及活产率。 | 极低质量证据表明，相比安慰剂，无治疗/标准治疗，抗氧化剂能提高活产率（OR 2.13，95%CI 1.45到3.12，P>0.001，8个RCT，651个女性，异质性I²=47%）。这表明生育力低下女性期待活产率20%，使用抗氧化剂可以提高到26%到43%。极低质量证据表明，抗氧化剂可以提高临床妊娠率(OR 1.52，95%CI 1.31到1.76，P<0.001，26个RCT，4271个女性，I²=66%)。组间的流产率没有差别(OR 0.79，95%CI 0.58到1.08，P=0.14，18个RCT，2834个女性，I²=23%，极低质量证据)。共35个试验报道了不良事件，但是不足以得出任何结论。 | 近期，发表于JAMA Network Open的一项来自荷兰的大型多中心、双盲、安慰剂对照随机临床试验（SUMMER试验），为这一争议提供了迄今为止最有力的答案。作为目前全球规模最大的男性生育抗氧化剂RCT之一，该研究共纳入1171名18-50岁的不育男性及其18-43岁的女性伴侣，覆盖荷兰21家生殖中心。该研究明确表明，对于整体男性不育人群而言，常规补充复合抗氧化剂如Impryl并不能提升自然妊娠率或辅助生殖成功率，也无法改善精液质量与精子DNA完整性。这一结果对临床实践具有重要指导意义：医生应避免将抗氧化剂作为不育男性的常规推荐，患者也应警惕市场上"提升精子质量"类保健品的夸大宣传。研究还提示"还原应激"（reductive stress）的可能性，即过度抗氧化可能干扰正常的氧化还原信号，反而损害精子功能。 |
| 2021 | English | 1 | May,2025 | Will wearing an ankle brace help heal achilles tendonitis? | https://www.alimed.com/blogs/splints-and-braces/choosing-an-ankle-brace-for-achilles-tendonitis | https://www.treatmyachilles.com/post/ankle-braces-for-achilles-tendonitis-do-they-work | https://www.donjoystore.com/injury-info-center/ankle-injury-guide/achilles-tendonitis | Ankle braces are designed to support the ankle and provide stability, reducing the risk of injury or further damage. Studies have shown that ankle braces can be effective in managing Achilles tendonitis by reducing swelling and inflammation in the achilles tendon. Typically, ankle braces work by applying pressure to the affected area, which can help alleviate pain and provide support to the tendon. By preventing excessive movement of the ankle joint, they can also help prevent further aggravation of the condition. | An ankle brace is designed to prevent your foot from turning sideways (i.e. in and out), not from going up and down, so it has little effect on the Achilles tendon. In any event, preventing your foot from going up and down is not good for the Achilles tendon. First, immobilising your Achilles tendon when you have a tendonitis can often make it feel even more uncomfortable than it already is. It needs a bit of movement to get rid of the stiff feeling. Second, the reason your Achilles tendon hurts when you have Achilles tendonitis is because it has lost a bit of its strength and therefore is struggling to cope with your normal activities. Keeping it still or limiting its movement drastically will cause it to lose more strength, because it's not getting any exercise. | As with any injury, your doctor will be the best source of advice on the appropriate treatment method for your situation. Achilles tendonitis treatment often includes the RICE method - rest, ice, compression, and elevation. Anti-inflammatory medications such as ibuprofen or aspirin may also be used to speed up recovery and reduce pain. Your doctor may also suggest physical therapy. The goal of physical therapy is to show you calf and heel stretches that help with the flexibility of the Achilles. As you return to activity, your doctor may suggest orthotics or an ankle brace to help support your Achilles tendon and to prevent the condition from reoccurring. |
| 2021 | English | 2 | May,2025 | Is a tepid sponge bath a good way to reduce fever in children? | https://www.sciencedirect.com/science/article/abs/pii/S0735675797900991 | https://pmc.ncbi.nlm.nih.gov/articles/PMC10665263/ | https://vicks.com/en-us/treatments/how-to-treat-the-flu/how-to-reduce-a-fever-associated-with-cold-or-flu | Tepid sponge baths distress febrile children, and their efficacy at reducing fever has not been established. This study compared fever reduction with (1) acetaminophen alone and (2) acetaminophen plus a 15-minute tepid sponge bath. Twenty children, ages 5 to 68 months, who presented to the emergency department or urgent care center with fever of ≥38.9°C were randomized. Sponge-bathed subjects cooled faster during the first hour, but there was no significant temperature difference between the groups over the 2-hour study period (P = .871). Subjects in the sponge bath group had significantly higher discomfort scores (P = .009). | Out of the 201 papers retrieved from the electronic search, two studies met the inclusion criteria. The meta-analysis found that tepid sponging was less effective than paracetamol at relieving fever two hours post-intervention (RR=0.25, 95% CI 0.08-0.79). Tepid sponging was not effective against fever. There is the need to modify existing local clinical protocols to reflect the new evidence and international guidelines. | Placing a cool, damp washcloth on your forehead and the back of your neck can help your fever symptoms feel better. You might also want to give yourself a sponge bath with cool water, focusing on high-heat areas like your armpits and groin. Normally, this method, known as tepid sponging, is done for about 5 minutes. Like staying in a cool environment and wearing light clothing, this physical measure for reducing a fever may not be as effective as fever-reducing medications. |
| 2021 | English | 3 | May,2025 | Can folic acid help improve cognition and treat dementia? | https://pmc.ncbi.nlm.nih.gov/articles/PMC1123448/ | https://www.aging-us.com/article/205788/text | https://www.sciencedirect.com/science/article/abs/pii/S0167494324002164 | The association of folate deficiency, as reflected by low concentrations of folate in serum, red cells, and cerebrospinal fluid and raised plasma concentrations of homocysteine, with depression and dementia has been confirmed in epileptic, neurological, psychiatric, geriatric and psychogeriatric patients. Some of the deficiency may be related to ageing, some may be secondary to mental illness, and some primary, but whether it is primary or secondary, open and controlled treatment studies confirm an aetiological link with specific effects of the vitamin on mood, drive, initiative, alertness, concentration, psychomotor speed, and social activity. With respect to dementia, there is evidence that folate deficiency may contribute to the cognitive impairment of the ageing brain, sometimes leading to reversible dementia but also increasing the risk of Alzheimer's disease and vascular dementia. | The systematic review included seven studies (with a total of 1102 participants, mean age 65--80 years), seven of which were appropriate for meta-analysis. Although a small number of studies found relatively large heterogeneity, the majority of studies showed significant benefit from folic acid supplementation, including the FSIQ (823 individuals, standardized mean difference [SMD] = 8.36, 95% confidence interval [CI] = 0.79 - 1.08). Folic acid supplementation had a significant effect on the reduction of most inflammatory cytokines, blood biomarkers of Alzheimer's disease, and Hcy. Folic acid supplementation seems to have a positive impact on cognitive function in older adults with mild cognitive impairment. | Vitamin B12 and folic acid could reduce blood homocysteine levels, which was thought to slow down the progression of Alzheimer's disease (AD), but previous studies regarding the effect of vitamin B12 and folic acid in treatment of AD have not reached conclusive results. Five studies that met the criteria were selected for inclusion in the meta-analysis. After a 6-month treatment, administration of vitamin B12 and folic acid improved the MMSE scores more than placebo did (SMD = 0.21, 95% CI = 0.01 to 0.32, p = 0.04) but did not significantly affect ADAS-Cog scores (SMD = 0.06, 95% CI = −0.22 to 0.33, p = 0.68) or measures of daily life function. Participants with AD who received 6 months of vitamin B12 and folic acid supplementation had better MMSE scores but had no difference in ADAS-Cog scores. Daily life function did not improve after treatment. |
| 2021 | English | 4 | May,2025 | Does duct tape work for wart removal? | https://health.clevelandclinic.org/will-duct-tape-remove-warts | https://www.healthline.com/health/duct-tape-for-warts | https://www.aad.org/public/diseases/a-z/warts-self-care | Duct tape occlusion is only an effective treatment for certain kinds of warts on certain parts of your body. Don't use duct tape if: Your wart is located near mucous membranes (like your mouth, your nasal cavity or around your eyes), or is in close proximity to your armpits or genitals. That skin is very sensitive and could tear. You have periungual or subungual warts (warts under and around your nails). You have genital warts. The strain of HPV that causes genital warts can also cause cervical cancer. You have plantar warts (warts on your foot). The skin on our feet is pretty tough. Not only is duct tape likely to be less effective on it, but the tape is also likely to fall off a lot. | Warts are caused by a virus within the body and can recur. Unlike other treatments, duct tape doesn't seek to treat the underlying virus that causes the wart or to identify the "root" of the wart. Instead, covering a wart with duct tape prevents the virus from spreading further by stopping the wart from contacting other parts of your skin. When you tear the tape off, the top layer of the wart will often come off with it. | To treat one or a few small warts at home, you can use: Salicylic acid; Duct tape; At-home freezing product. At-home treatment can be effective if you have one or a few small warts. Keep in mind that at-home treatment isn't right for everyone. You should see a dermatologist if you have: Warts on your face or genital area; A wart that is changing, hurts, itches, burns, or bleeds; Many warts; Doubts about whether you have a wart(s); Tried at-home treatment and cannot get rid of the wart(s); A weakened immune system (cancer, HIV, organ transplant); Diabetes. |
| 2021 | English | 6 | May,2025 | Can vitamin b12 and sun exposure together help treat vitiligo? | https://www.mayoclinic.org/diseases-conditions/vitiligo/diagnosis-treatment/drc-20355916 | https://pmc.ncbi.nlm.nih.gov/articles/PMC9515252/ | https://pubmed.ncbi.nlm.nih.gov/9394983/ | Limited studies show that the herb Ginkgo biloba may return skin color in people with vitiligo. Other small studies show that alpha-lipoic acid, folic acid, vitamin C and vitamin B-12 plus phototherapy may restore skin color for some people. As with any nonprescription treatment, check with your health care provider before trying alternative medicine therapies to be sure they won't interfere with other treatments you're using. If you have vitiligo, the following self-care tactics may help you care for your skin and improve its appearance: Protect your skin from the sun and artificial sources of UV light. Use a broad-spectrum, water-resistant sunscreen with an SPF of at least 30. | Most studies agree that combining vitamin B12, folic acid, and sun exposure is good for inducing repigmentation. Supplementation of zinc and phenylalanine when used in conjunction with topical steroids or UV-B (ultraviolet B) treatment shows therapeutic effects on vitiligo due to their role in the melanin synthesis pathway. Investigations conducted on herbal supplements have revealed that most of them contain antioxidants, which aid in repigmentation. | The aim of this 2-year study was to test the hypothesis that folic acid, vitamin B12 and sun exposure could be helpful in treating vitiligo. One hundred patients with vitiligo were treated with oral folic acid and vitamin B12. Clear repigmentation occurred in 52 patients, including 37 who exposed their skin to summer sun and 6 who used UVB lamps in winter. Total repigmentation was seen in 6 patients. The spread of vitiligo stopped in 64% of the patients after treatment. Folic acid and vitamin B12 supplementation combined with sun exposure can induce repigmentation better than either the vitamins or sun exposure alone. Treatment should continue as long as the white areas continue to repigment. |
| 2021 | English | 10 | May,2025 | Will taking birth control pills treat an ovarian cyst? | https://pmc.ncbi.nlm.nih.gov/articles/PMC10964840/ | https://www.mayoclinic.org/diseases-conditions/ovarian-cysts/diagnosis-treatment/drc-20353411 | https://www.aafp.org/pubs/afp/issues/2014/1101/p623.html | We identified eight randomized controlled trials from four countries; the studies included a total of 686 women. Treatment with combined oral contraceptives did not hasten resolution of functional ovarian cysts in any trial. This held true for cysts that occurred spontaneously as well as those that developed after ovulation induction. Most cysts resolved without treatment within a few cycles; persistent cysts tended to be pathological (e.g., endometrioma or para‐ovarian cyst) and not physiological. Although widely used for treating functional ovarian cysts, combined oral contraceptives appear to be of no benefit. Watchful waiting for two or three cycles is appropriate. | Treatment depends on your age and the type and size of your cyst. It also depends on your symptoms. Your health care provider might suggest: Medicine. Hormonal contraceptives, such as birth control pills, keep you from ovulating. This may keep you from getting more ovarian cysts. But, birth control pills won't shrink an existing cyst. | Oral contraceptives are not an effective treatment for ovarian cysts, whether the cysts are spontaneous or associated with medically induced ovulation. Most cysts resolve without intervention within two to three months. Those that do not resolve in this time frame are more likely to be pathologic in nature and should prompt referral for a surgical evaluation. (Strength of Recommendation: B, based on inconsistent or limited-quality patient-oriented evidence.) |
| 2021 | English | 12 | May,2025 | Can evening primrose oil help treat eczema? | https://www.cochrane.org/evidence/CD004416_oral-evening-primrose-oil-and-borage-oil-eczema | https://pubmed.ncbi.nlm.nih.gov/3307886/ | https://nationaleczema.org/blog/evening-primrose-oil-and-eczema/ | There was some evidence of mild and temporary side-effects for participants with either product or placebo, which were mainly mild, and included temporary headache and upset stomach or diarrhoea. With evening primrose oil (EPO) there is an anticoagulant (blood-thinning) effect when taking these products. This systematic review found no evidence that either BO or EPO are effective in treatment of eczema. Both of these products and the placebos used in the studies had similar mild, temporary side-effects, which were mainly gastrointestinal. | In a double-blind trial patients with atopic eczema received either oral evening primrose oil (EPO) (n = 14) or placebo (n = 11) for 12 weeks. In the EPO group a statistically significant improvement was observed in the overall severity and grade of inflammation and in the percentage of the body surface involved by eczema as well as in dryness and itch. The patients receiving EPO showed a significantly greater reduction in inflammation than those receiving placebo. Evening primrose oil caused a significant rise in the amount of dihomogammalinolenic acid in the plasma phospholipid fatty acids. | Research studies on evening primrose oil aren't as plentiful as others, and results range from promising to inconclusive. "We found some compelling data for evening primrose oil," said Dr. Peter Lio. "Evening primrose oil has high levels of gamma-linolenic acid and omega-6 fatty acids, and both are thought to help with barrier repair and have some anti-inflammatory properties." Other studies are less conclusive about the use of evening primrose oil for eczema. In 2013, researchers determined evening primrose oil in its various forms are "not effective treatments for eczema." Yet, in direct contrast, a 2018 study found evening primrose oil "has shown significant improvement in skin dryness, pruritus and range of lesions" in patients with atopic dermatitis. |
| 2021 | English | 17 | May,2025 | Can fermented milk help mitigate high blood pressure? | https://pmc.ncbi.nlm.nih.gov/articles/PMC11500820/ | https://www.sciencedirect.com/science/article/pii/S0002916523056101 | https://www.ahajournals.org/doi/10.1161/hypertensionaha.114.03469 | The review does not support an effect of fermented milk on blood pressure. Despite the positive effect on SBP the authors conclude, for several reasons, that fermented milk has no effect on blood pressure. The effect found was very modest and only on SBP, the included studies were very heterogeneous and several with weak methodology. Finally, sensitivity and subgroup analyses could not reproduce the antihypertensive effect. The results do not give notion to the use of fermented milk as treatment for hypertension or as a lifestyle intervention for pre‐hypertension nor would it influence population blood pressure. | In a randomized placebo-controlled study, 39 hypertensive patients received 150 mL/d of either L. helveticus LBK-16H fermented milk or a control product for 21 wk after a 2-wk run-in period. There was a mean difference of 6.7 ± 3.0 mm Hg in systolic blood pressure (P = 0.030) and of 3.6 ± 1.9 mm Hg (P = 0.059) in diastolic blood pressure between the test product and control groups. L. helveticus LBK-16H fermented milk containing bioactive peptides in normal daily use has a blood pressure--lowering effect in hypertensive subjects. | Previous human clinical trials have shown that probiotic consumption may improve blood pressure (BP) control. Nine trials were included. Probiotic consumption significantly changed systolic BP by −3.56 mm Hg (95% confidence interval, −6.46 to −0.66) and diastolic BP by −2.38 mm Hg (95% confidence interval, −2.38 to −0.93) compared with control groups. A greater reduction was found with multiple as compared with single species of probiotics, for both systolic and diastolic BP. The present meta-analysis suggests that consuming probiotics may improve BP by a modest degree, with a potentially greater effect when baseline BP is elevated, multiple species of probiotics are consumed, the duration of intervention is ≥8 weeks, or daily consumption dose is ≥10^11 colony-forming units. |
| 2021 | English | 22 | May,2025 | Does Aleve relieve migraine headaches? | https://pmc.ncbi.nlm.nih.gov/articles/PMC6540401/ | https://www.mayoclinic.org/drugs-supplements/sumatriptan-and-naproxen-oral-route/description/drg-20071750 | https://www.migraineagain.com/over-the-counter-migraine-treatments-headache-relief-at-home/ | Naproxen is statistically superior to placebo in the treatment of acute migraine, but the NNT of 11 for pain‐free response at two hours suggests that it is not a clinically useful treatment. Cochrane reviews examining other commonly used analgesics for acute migraine have reported better (lower) NNT results for the same outcome. Naproxen is not clinically useful as a stand‐alone analgesic in acute migraine, as it is effective in fewer than 2 people in 10. | Sumatriptan and naproxen combination is used to treat acute migraine attacks. It is not used to prevent migraine headaches or to treat cluster headaches. This medicine often relieves other symptoms that occur together with a migraine headache, such as nausea, vomiting, sensitivity to light, and sensitivity to sound. Sumatriptan is a serotonin receptor agonist that works in the brain to relieve migraine headaches. Naproxen is an NSAID. This medicine is available only with your doctor's prescription. | The most common OTC medications available to treat migraine attacks contain acetaminophen or a nonsteroidal anti-inflammatory drug (NSAID), like aspirin, ibuprofen, or naproxen. Some OTC medications for migraine will also contain caffeine or a combination of the above medications. An example of this is Excedrin Migraine, which has acetaminophen/aspirin/caffeine and is likely one of the more popular OTC medications for the treatment of migraine. |
| 2021 | English | 28 | May,2025 | Does steam from a shower help croup? | https://www.mayoclinic.org/diseases-conditions/croup/diagnosis-treatment/drc-20350354 | https://healthcare.utah.edu/the-scope/kids-zone/all/2023/01/home-treatments-croup-will-help-your-childs-barking-cough | https://www.uofmhealth.org/health-lab/croup | Croup often runs its course within 3 to 5 days. In the meantime, keep your child comfortable with a few simple measures: Stay calm. Comfort or distract your child — cuddle, read a book or play a quiet game. Crying makes breathing more difficult. Provide humidified or cool air. Although there's no evidence of benefit from these practices, many parents believe that humid air or cool air helps a child's breathing. For moist air, you can use a humidifier. You can also sit with the child in a bathroom filled with steam created by running hot water from the shower. If it's cool outside, you can open a window for your child to breathe the cool air. | So how can you treat stridor? One of the best things to do when you're at home is get the shower all steamed up and get your child in the bathroom, because warm, moist air seems to work best to relax the vocal cords and break the stridor. A humidifier, not a hot vaporizer, but a cool mist humidifier also will help with getting the swelling down. Cold air also helps relieve stridor. If it's cold outside, take your child outdoors. If your child continues to have stridor, take them to the closest children's emergency room. Your child may receive a dose of a steroid called dexamethasone, which helps shrink the swelling in your child's airways. In severe cases, they may receive a breathing treatment of a medicine called racemic epinephrine. | Because croup is a virus, antibiotics do not help. Giving your child over-the-counter cold medications will not help either. However, the following tips may help the cough and stridor: Run a hot shower to create steam. Do NOT put your child in the hot shower. Instead, close the bathroom door and let the room fill with steam. Have your child breathe in the moist air for 10--15 minutes. If your child's breathing does not get better with steam, take him or her outside. Cold, moist air can decrease the swelling and help the coughing and stridor. Offer cold liquids and popsicles to help with the coughing. Try to keep your child calm. |
| 2021 | English | 43 | May,2025 | Does Tylenol manage the symptoms of osteoarthritis? | https://pmc.ncbi.nlm.nih.gov/articles/PMC8275921/ | https://www.mayoclinic.org/diseases-conditions/osteoarthritis/diagnosis-treatment/drc-20351930 | https://www.aafp.org/pubs/afp/issues/2020/0715/p113.html | The evidence to date suggests that NSAIDs are superior to acetaminophen for improving knee and hip pain in people with OA. The size of the treatment effect was modest, and the median trial duration was only six weeks, therefore, additional considerations need to be factored in when making the decision between using acetaminophen or NSAIDs. In OA subjects with moderate‐to‐severe levels of pain, NSAIDs appear to be more effective than acetaminophen. | Osteoarthritis can't be reversed, but treatments can reduce pain and help you move better. Medicines that can help relieve osteoarthritis pain symptoms include: Acetaminophen. Acetaminophen (Tylenol, others) has been shown to help some people with osteoarthritis who have mild to moderate pain. Taking more than the recommended dose of acetaminophen can cause liver damage. Nonsteroidal anti-inflammatory drugs (NSAIDs). Common pain relievers, such as ibuprofen (Advil, Motrin IB, others) and naproxen sodium (Aleve), taken at the recommended doses, typically relieve osteoarthritis pain. NSAIDs can cause stomach upset, cardiovascular problems, bleeding problems, and liver and kidney damage. NSAIDs as gels, applied to the skin over the affected joint, have fewer side effects and may relieve pain just as well. | Acetaminophen is no better than placebo for relief of hip or knee pain due to osteoarthritis. Although acetaminophen does provide some pain relief, the effect is small and not clinically significant. (Strength of Recommendation [SOR]: A, based on systematic reviews and meta-analyses.) Higher daily dosages or extended-release formulations also are not effective for relieving hip or knee pain due to osteoarthritis. (SOR: A, based on a meta-analysis and a large randomized controlled trial [RCT].) |
| 2021 | English | 45 | May,2025 | Can omega-3 treat borderline personality disorder in women? | https://pubmed.ncbi.nlm.nih.gov/12505817/ | https://pmc.ncbi.nlm.nih.gov/articles/PMC10434080/ | https://www.sciencedirect.com/science/article/pii/S241464472030004X | Twenty subjects were randomly assigned to 1 g of E-EPA; 10 subjects were given placebo. Analyses that used random-effects regression modeling and controlled for baseline severity showed E-EPA to be superior to placebo in diminishing aggression as well as the severity of depressive symptoms. The results of this study suggest that E-EPA may be a safe and effective form of monotherapy for women with moderately severe borderline personality disorder. | Available data indicate that marine omega-3 fatty acids improve BPD symptoms, particularly impulsive behavioral dyscontrol and affective dysregulation, reducing depressive symptoms and suicidal tendencies. Marine omega-3 fatty acids could be considered as a complementary therapy for the improvement of severe symptoms associated with patients with BPD. For supplemental omega-3 fatty acids, significant effects were found in one study (n = 49) for reduction in suicidality (RR = 0.52, 95% CI 0.28 to 0.95) and depressive symptoms (RR = 0.48, 95% CI 0.28 to 0.81). | In humans, dietary deficiencies of omega-3 fatty acids are associated with an increased risk of developing various psychiatric disorders, including depression, bipolar disorder, schizophrenia, dementia, attention-deficit/hyperactivity disorder, and autism. In particular, eicosapentaenoic and docosahexaenoic acid have been linked to the maintenance of mental health, and their deficits have been implicated in the pathophysiology of mental disorders. This may be mediated by the modulation of inflammatory processes and their direct effects on neuronal membrane fluidity and receptor function. However, randomized clinical trials that have investigated the therapeutic effects of omega-3 fatty acids have yielded inconclusive results, thereby limiting the use of these nutrients in psychiatric practice. |
| 2021 | English | 50 | May,2025 | Will taking antioxidant supplements treat fertility problems? | https://jamanetwork.com/journals/jamanetworkopen/fullarticle/2839329 | https://www.sciencedirect.com/science/article/pii/S0015028218304291 | https://pmc.ncbi.nlm.nih.gov/articles/PMC10135990/ | A total of 1171 men (median [IQR] age, 34 [31-38] years; female partners' median [IQR] age, 32 [30-35] years) were included in the data analysis, of whom 591 were in the antioxidant supplement group and 580 were in the placebo group. Ongoing pregnancy rate within 6 months was not significantly different between the 2 groups (193 of 571 [33.8%] vs 208 of 555 [37.5%]; adjusted odds ratio [AOR], 0.85 [95% CI, 0.66-1.09]; P = .20). Within the window of optimal treatment effect between 4 and 6 months (considering a spermatogenesis cycle of 72 days), ongoing pregnancy rate was significantly lower in the antioxidant supplement group compared with the placebo group (69 of 446 [15.5%] vs 95 of 442 [21.5%]; AOR, 0.66 [95% CI, 0.47-0.94]; P = .02). This randomized clinical trial found that ongoing pregnancy rates did not improve with the antioxidant supplement compared with a placebo. Therefore, the investigators do not support its use in men seeking fertility care. | There is evidence that oxidative stress plays an important role in male and female subfertility. Antioxidants could be an inexpensive treatment or an adjunct to reproductive techniques such as IVF and ICSI to improve fertility outcomes. However, although the evidence to date suggests a treatment benefit, further large, well-designed, placebo-controlled trials reporting on clinically important outcomes such as ongoing pregnancy and the live-birth rate are needed to truly understand the role of antioxidants. | Oxidative stress (OS) has an important role in female reproduction, whether it is ovulation, endometrium decidualization, menstruation, oocyte fertilization, or development and implantation of an embryo in the uterus. The menstrual cycle is regulated by the physiological concentration of reactive forms of oxygen and nitrogen as redox signal molecules. It has been suggested that the decline in female fertility is modulated by pathological OS. The pathological excess of OS compared to antioxidants triggers many disorders of female reproduction which could lead to gynecological diseases and to infertility. Therefore, antioxidants are crucial for proper female reproductive function. Compensation for low levels of antioxidants through their supplementation can improve fertility. |
| 2022 | Chinese | 1 | May,2025 | 茶袋有助于拔牙后的止血吗？ | https://heho.com.tw/archives/259567 | https://gtcdental.com/ch/ch-treatment-aftercare/bayazhiliaohouhuli/ | https://www.51dentist.com/front.php?r=news/healthdetail&id=4319 | 建议，经过科学实证，可以咬茶叶包代替，「茶叶当中有单寧酸，单寧酸本身有血管收缩的功能，咬在拔牙的伤口有助於促进血管收缩、帮助止血。」不过张起华医师强调，茶叶包为替代方案，无菌纱布仍为止血首选。 | 茶含有丹宁酸，促使血管收缩，帮助血凝块形成。敷茶包或许能帮助减少出血。如果拔牙后一小时，你发现伤口有些渗血，不妨将湿茶包敷在拔牙部位，紧紧咬着，压住伤口。敷20到30分钟。喝冷茶或许也有帮助，但是直接在伤口敷茶包的效果更好。 | 通过正确的拔牙治疗后护理，确保最佳愈合效果并预防并发症。我们提供了疼痛管理、口腔卫生和快速康复的基本技巧。咬住纱布30分钟。如果出血不止，请更换新的纱布。也可以咬湿润的茶包，因为茶叶中含有鞣酸，有助于止血。 |
| 2022 | Chinese | 3 | May,2025 | 胶原蛋白补充剂能治愈骨关节炎吗？ | https://continentalhospitals.com/zh-CN/blog/benefits-of-collagen-protein-for-skin-and-joints/ | https://www.ahb-lab.com/zh/icppx/collagen-peptides-your-comprehensive-guide-to-managing-osteoarthritis/ | https://www.zhihu.com/tardis/zm/art/590661711 | 多年来，研究人员一直在研究胶原蛋白对关节炎的影响，但研究结果并不一致。科学家认为，胶原蛋白可能会触发身体产生抗炎物质，从而缓解关节疼痛，但这一说法尚未得到证实。此外，胶原蛋白中的成分，如软骨素和氨基葡萄糖，被认为有助于重建软骨。2017年的一项研究发现，口服胶原蛋白补充剂可改善疼痛、晨僵和关节肿胀，但指出胶原蛋白不能替代或与典型的 RA 药物一样有效。然而，其他研究表明，胶原蛋白对缓解 RA 症状无效。因此，需要更多的实质性研究来确定胶原蛋白是否可以成为 RA 治疗的有效成分。 | 胶原蛋白有助于维持关节表面的光滑。随着年龄的增长，软骨会磨损，您可能会感到僵硬或不适。胶原蛋白补充剂可以维持软骨健康，减少关节摩擦。胶原蛋白有助于保持关节的灵活性和活动能力。它支撑骨骼之间的软组织和软骨，使关节活动更顺畅。 | 由于胶原蛋白构成了我们身体的许多重要部分，因此它对保持身体健康非常有帮助，也是必不可少的。尽管有大量证据表明骨关节炎补充剂虽然胶原蛋白可以减轻骨关节炎疼痛，保持皮肤水润，但要充分了解其潜在的健康优势，如降低血压和血糖水平，还需要进一步的研究。 |
| 2022 | Chinese | 10 | May,2025 | 深蹲对膝盖有害吗？ | https://dxy.com/article/50066 | https://zhuanlan.zhihu.com/p/52780123 | https://www.womenshealthmag.com/tw/fitness/work-outs/g33230061/is-true-that-squat-make-ankle-hurt/ | 在任何一种深蹲过程中，最大的肌肉力量作用在膝关节上的时刻发生在从下降切换到上升的过程中。低杠深蹲是下蹲至最深度，这样的深蹲方式可以比其他方式更充分的拉伸腘绳肌。与半蹲相比这意味着腘绳肌可以更多的参与离心和向心训练的部分。当股四头肌收缩以伸展膝关节时，伸展的这一过程是通过附着点在胫骨上的股四头肌施加向前伸展的力（股四头肌是跨关节的肌肉）。由于部分下蹲缺乏腘绳肌产生的力量，所以膝盖前方经常涉及更大的负荷。但是深蹲却显示出膝盖惊人的对称性。腘绳肌产生的拉力与股四头肌的拉力相反。通过最大程度的增加腘绳肌对运动的作用，我们正在平衡这两个力，并且膝盖上没有足够的剪切力去伤害到韧带（如ACL和PCL）。这就是为什么会通过深蹲这个动作去增强腘绳肌和股四头肌对于恢复ACL撕裂是至关重要的原因。 | 1986年，有研究人员比较了举重、篮球运动员和跑步者之间的韧带松弛程度，经过一段时间的深蹲训练后，发现篮球运动员和跑步者有短暂的膝盖松弛变化，而举重运动员并没有显著的变化。1989年，另一组研究人员将举重运动员膝关节与未经过深蹲训练的人进行比较，结论证明深蹲并没有影响膝盖的稳定性，相反部分人的膝盖稳定性更好。他们的研究都指向了同一个结论——深蹲其本身是一项安全的运动，也不会对你的膝盖造成损伤。 | 以前總有「深蹲會傷膝蓋」的誤解，但隨著健身風氣越盛行，越來越多人開始探討深蹲跟膝蓋之間的關係。事實上只要深蹲動作正確，並在自身體能合理範圍下訓練，反而是可以預防膝蓋傷害的一個好動作。 |
| 2022 | Chinese | 16 | May,2025 | 右撇子被迫改用左手会导致语言问题吗？ | https://m.thepaper.cn/newsDetail_forward_16567911 | https://news.cctv.cn/2025/08/13/ARTIe0MJPyM6hoBPDYOosOUq250813.shtml | https://zhuanlan.zhihu.com/p/68235822 | 医学心理学家认为，如果强迫左撇子改用右手，使孩子已建立的大脑优势半球从右侧改为左侧，常会造成原有的语言中枢混乱，最常见的症状就是口吃，也有的孩子唱歌走调，发音不准。所以，强行纠正后果很严重。 | 左撇子不是病，只是一种自然现象，不必强制改用右手。若强制改用右手，会让左撇子已经建立的大脑优势半球强制更改，或出现记忆力障碍、注意力障碍、左右混淆等，还可能造成心理创伤。 | 根据2010年发表在Cortex期刊上的一项名为Right-shift for non-speech motor processing in adults who stutter的研究证明，左撇子的原因是他们的左侧语言相关区域因为存在某种潜在的损伤，所以导致大脑不得不选择右半脑为主要语言区域。如果强行左手改右手，那么会使得语言处理往原本就有问题的左侧偏移，导致语言运动控制出现问题。 |
| 2022 | Chinese | 18 | May,2025 | 粉盐对你有好处吗？ | https://blog.wenxuecity.com/myblog/52434/202302/28605.html | https://www.sohu.com/a/358817461_720471 | https://auyx.au/news/details/281504 | 喜马拉雅盐，又名玫瑰盐，是一种产自巴基斯坦东部旁遮普省克乌拉盐矿的石盐。可食用，也可用于装饰用途。没有科学证据能证明喜马拉雅盐相比普通食盐对人体有更多的好处，因此任何声称喜马拉雅盐对人体有特殊益处的说法应被视为市场营销或伪科学。 | 喜马拉雅盐90%的成分为氯化钠，还富含多种微量矿物质，如钾、镁、钙、磷等高达84种微量矿物质，对人体十分有益。关键是，比普通的盐，加工工序更少，且不含有添加剂和重金属。钠的含量也比普通食盐略低。至于网络上很多宣传的，喜马拉雅盐还能缓解支气管炎、治感冒，提高性欲等等，城市君无法考证。 | 最近，澳洲的一项全球首次研究表明，尽管喜马拉雅粉盐被吹捧为既健康又貌美，但实际上粉盐的基本营养素含量相比普通白盐并没有显著的提高，并且粉盐还有低含量的有害重金属。除了含有有害金属之外，尽管目前的研究发现粉盐比白食盐含有更多的营养成分，但是由于含量营养成分含量较低，人类必须摄入超过日健康摄入盐量的500%以上，其中的营养成分才能对人体产生作用。因此澳大利亚营养研究所得首席执行官表示，虽然粉盐比白盐含有更多的矿物质，但这个量是没有意义的，限制盐的每日摄入量才符合世界卫生组织的建议，以及当代人们对健康生活的认可。 |
| 2022 | Chinese | 20 | May,2025 | 鱼油可以改善胆固醇水平吗？ | https://www.mayoclinic.org/zh-hans/drugs-supplements-fish-oil/art-20364810 | https://www.mayoclinic.org/zh-hans/diseases-conditions/high-blood-cholesterol/in-depth/cholesterol/art-20045192 | https://jiankang.cctv.com/2025/03/28/ARTI0ca9RxmCZGZMZXK8UefV250327.shtml | 高甘油三酯类和胆固醇。有充分的证据表明，Ω-3 脂肪酸可以显著降低血液中的甘油三酯水平。高密度脂蛋白胆固醇（HDL，也被称之为"好胆固醇"）水平似乎也有轻微改善，但低密度脂蛋白胆固醇（LDL，也被称之为"坏胆固醇"）水平也有所上升。 | 高脂肪鱼含有高水平的 ω-3 脂肪酸。ω-3 脂肪酸可以降低甘油三酯类（血液中的一种脂肪）水平。这些脂肪酸还有助于降低血压和出现血凝块的风险。对于曾出现心脏病发作的患者，ω-3 脂肪酸可以降低猝死的风险。ω-3 脂肪酸不会影响 LDL 胆固醇的水平，但却有助于降低甘油三酯类的水平，并升高 HDL（有益胆固醇）的水平。但由于 ω-3 脂肪酸对心脏有益，美国心脏协会建议人们每周至少摄入两份鱼类。烘焙或烤制鱼肉时，避免加入不健康的脂肪。 | 深海鱼油是从深海鱼类如鲑鱼、沙丁鱼、鳕鱼等的脂肪组织中提取的油脂，其主要活性成分为Omega-3多不饱和脂肪酸，包括EPA（二十碳五烯酸）和DHA（二十二碳六烯酸）。这两种成分是人体必需但无法自行合成的营养素，需通过食物或补充剂摄入。Omega-3脂肪酸在人体内具有多重生理功能，例如调节血脂、抗炎、改善血管弹性等。其中，EPA以降低血液中的甘油三酯为主，而DHA则对大脑和视力发育有重要作用。避免误区。不盲目停用降压或降脂药物，不随意增加剂量。刘迎午主任特别提醒市民，深海鱼油无法降低总胆固醇和低密度脂蛋白（LDL即"坏胆固醇"），更不能替代他汀类药物对胆固醇的管理。 |
| 2022 | Chinese | 23 | May,2025 | 水杨酸在欧洲被禁用吗？ | https://www.cirs-group.com/cn/cosmetics/ou-meng-ban-bu-hua-zhuang-pin-xin-gui-zai-jin-yong-duo-zhong-cmr-wu-zhi-shui-yang-suan-jia-zhi-huo-mian | https://www.cirs-group.com/cn/cosmetics/ou-meng-sccs-fa-bu-guan-yu-shui-yang-suan-de-zui-zhong-an-quan-yi-jian | https://www.10100.com/article/8057967 | 2022年9月15日，欧盟发布了最新法规(EU) 2022/1531，对十几种致癌、致突变或生殖毒性（CMR）物质实施了禁令和限制，并更新了欧盟化妆品法规（EC）No 1223/2009的附录II、附录III和附录V相关规定。该法规自发布日起20天后生效，其中关于CMR物质的禁令于2022年12月17日生效。尽管水杨酸甲酯（CAS号：119-36-8）被分类为生殖毒性2类物质，但SCCS评估认为其在不超过浓度限制前提下在化妆品中的使用是安全的。法规更新了附件III限用清单中水杨酸甲酯的使用限量。 | 2023年6月9日，欧盟消费者安全科学委员会（SCCS）发布关于水杨酸（salicylic acid, CAS No. 69-72-7, EC No. 200-712-3）在化妆品中的最终安全意见（SCCS/1646/22）。依据目前的数据，并且考虑到水杨酸潜在的内分泌干扰特性，SCCS认为水杨酸作为防腐剂，在化妆品中浓度为0.5%是安全的：水杨酸是一种眼睛刺激物，可能会对眼睛造成严重损害。除了作为防腐剂，水杨酸在化妆品中作为其他用途使用时的最大安全使用浓度分别为：3.0%用于淋洗类发用产品；2.0%用于其他化妆品；0.5%用于身体乳液、眼影、睫毛膏、眼线笔、口红、口腔（口部）产品和非喷雾型除臭剂。SCCS暂未得出水杨酸在化妆品中使用的最大安全浓度。 | 3月12日，欧盟官方公报发布通告，对化妆品法规（EC）No 1223/2009附件III进行修订，明确限制洗护类化妆品中水杨酸（2-羟基苯甲酸）类制剂的浓度及使用条件。即日起，凡不符合相关限值和使用要求的含水杨酸洗护产品，不得在欧盟市场销售或投放。 |
| 2022 | Chinese | 29 | May,2025 | 嚼口香糖有助于减肥吗？ | https://zhuanlan.zhihu.com/p/140335470 | http://edu.people.com.cn/n/2015/0408/c1053-26811030-2.html | https://jingyan.baidu.com/article/11c17a2c0c1519f446e39d1f.html | 在微信上，关于口香糖减肥的论点，有一种解释是"饿了嚼一嚼口香糖，这样就会让大脑产生饱腹感，坚持这样做就能达到减肥瘦身目的。"事实真是这样吗？南京市妇幼保健院营养科戴永梅主任说，嘴部的咀嚼动作确实能伪造一种吃东西的表象，让大脑会认为你是正在吃东西，释放停止饥饿信息。但如果长时间咀嚼，会让胃释放过多胃酸消化液，灼伤胃黏膜引发恶心、腹痛。同时口香糖的化学原料让水果和蔬菜都失去了原有的风味，让人们的味觉变得不敏感，因此经常嚼口香糖的人口味更重，就餐时吃下的高热量甜食比别人要多。 | 谎言一：口香糖咀嚼动作能瘦脸。话说最新的一项研究表明，如果一个人除睡觉之外，整天都咀嚼无糖型口香糖的话，他很有希望在一年之内减掉5公斤赘肉，因为这样的"运动"每小时可以消耗掉70千卡的热量。真相：面部最容易堆积脂肪的是轮廓边缘和面颊部分，通过咀嚼口香糖确实能起到让面部肌肉运动的功效。但咀嚼时间超过15分钟，口香糖中的大量凝胶类物质开始发挥作用，口香糖就变得越来越难嚼。过度用力会让面部肌肉紧绷，咀嚼时间太长对面部骨骼和肌肉是不利的。 | 总结一下，嚼口香糖可能对一些人有好处，但对另一些人却没什么好处。因为口香糖本身没有任何天然成分能促进减肥，除非你努力改善饮食习惯，并将日常锻炼纳入其中。 |
| 2022 | Chinese | 38 | May,2025 | 咖啡有助于减肥吗？ | https://www.mayoclinic.org/zh-hans/healthy-lifestyle/weight-loss/expert-answers/calories/faq-20058100 | https://m.thepaper.cn/baijiahao_12557334 | http://health.people.com.cn/n1/2022/0809/c14739-32497962.html | 这取决于您喝咖啡的习惯。一杯纯的现煮咖啡热量不足 5 卡路里，并且没有脂肪。但如果您在咖啡中添加配料，卡路里就会增加。无论是在家里还是在咖啡店都是如此。饮料里经常加糖者比不加糖者更容易出现体重增长。一项研究发现，每天往咖啡中加一茶匙糖与体重增长有关。偶尔喝一杯加配料的咖啡饮料没什么问题。但就减重而言，每一份卡路里都有影响。包括液体形式的卡路里。 | 咖啡有减肥作用这一说法不是空穴来风。因为咖啡里含有咖啡因，而咖啡因有利尿作用。既然能利尿，自然可以排除体内的水分，体重当然也会下降。但是利尿是不能减肥的，利尿排掉的是水分，不是脂肪。虽然体重下降，但是脂肪没有少。只要喝足够的水，体内水分得到补充以后，体重立马就回来了。除此之外是因为咖啡因有兴奋作用，可以提高基础代谢率。因为咖啡因有刺激中枢神经的作用，让心跳加快，基础代谢也在加快。同时咖啡的热量很低，并且，咖啡中的咖啡因是脂肪分解的催化剂，能够加快脂肪分解。 | 很多人喝咖啡时喜爱添加牛奶或糖，牛奶中本身就含有脂肪，糖的额外摄入不利于减肥。即便只喝黑咖啡，如果喝得少，必然没有减肥效果。但长期过量摄入咖啡易上瘾，不喝咖啡便萎靡不振、提不起精神。"控制能量的摄入并增加消耗是减肥的本质机理。"孟凡强说，"管住嘴，迈开腿"的减肥方式更适合肥胖和超重人群。另外，"管住嘴"比"迈开腿"在控制体重上"性价比"更高。"如果从能量代谢的角度出发，'管住嘴'更易达到减肥效果。一般情况下，人快速步行一小时约消耗200千卡，而喝一听330毫升的可乐就能摄入约150千卡能量，吃50克巧克力约摄入300千卡，100克红烧肉近500千卡。" |
| 2022 | Chinese | 41 | May,2025 | 皮肤标签会传染吗？ | https://www.skindoctor.com.tw/post/皮贅（skin-tags） | https://www.warts.hk/zh-hk/skin-tag/ | https://voi.id/zh/info-sehat/187547 | 初得皮膚贅瘤的人，通常都會無法接受會繼續慢慢長出皮膚贅瘤的現實，而在門診中質問這個疾病的病因，希望可以藉此知道預防的方法！但是很可惜的，目前皮膚贅瘤的確切成因，仍然是個謎。儘管皮膚科醫師提出很多的理論，還是無法了解為何會莫名地產生纖維組織的增生，造成肉眼看到的一顆顆膚色小腫瘤。但是可以確定的，它絕對和任何的細菌病毒無關，所以當然不會傳染，更對健康無害。 | 皮膚瘜肉一般呈凸起的粒狀、袋狀或絲狀，常見於頸部，胸部下方或腋下。呈膚色或暗啡色，大約有幾毫米大小，形狀不一，擠壓時沒有分泌物或血液流出。皮膚瘜肉不具傳染性，亦非因感染所致，甚少引起痛楚。 | 皮赘不具有传染性。皮赘在临床上表现为无质地的生长肉，不具有传染性。它通常不会引起疼痛或不适。只是，它通常被认为是一种干扰外观的情况。 |
| 2022 | Chinese | 48 | May,2025 | 喝苹果醋有助于减肥吗？ | https://www.mayoclinic.org/zh-hans/healthy-lifestyle/weight-loss/expert-answers/apple-cider-vinegar-for-weight-loss/faq-20058394 | https://www.news.cn/food/20250408/90afd9cfe70742c686ecb64b38353cf1/c.html | https://m.baidu.com/bh/m/detail/qr_6518207843030668153 | 苹果醋不太可能导致减重。研究尚未证明这种压碎苹果后制成的发酵果汁能帮助人们减肥。一些人声称苹果醋有许多有益健康的作用，比如减重。他们说在餐前喝少量苹果醋或服用苹果醋补充剂有助于控制饥饿感并燃烧热量。但几乎没有研究能支持这一说法。专家尚未发现使用苹果醋能实现有意义的减重或长期的饥饿控制。而且许多关于苹果醋和减重的研究规模较小或是有其他不足。一些小型研究表明，苹果醋可能有潜力作为减重辅助剂。但还需要更多的研究。 | 对人类进行的13项研究，样本量过小（大多不到40人）、干预周期短（最长仅为12周）、实验上也存在设计缺陷，减肥效果微弱且不一致，研究质量偏低，还有很多作用机制尚未明确。最后得出的结论是：苹果醋有减肥效果这件事证据不足，需要更多低偏倚风险的大规模、长期临床研究。 | 睡觉前喝苹果醋减肥吗？病情分析：可以的，其实效果也不大。注意不要多喝水。发酵的苹果含有果胶，这种果胶被认为有助于降低脂肪含量。所以减肥的人需要做的就是在每餐前喝几勺苹果醋，这样就可以轻松的燃烧掉多余的脂肪。减肥并不意味着一定要饿的靠前胸和后背。合理的饮食不仅能维持身体健康，保证充分的活力，还能减轻心理上的不满足感，防止暴食和精神崩溃。如果长期按此安排饮食，还可以养成良好的饮食习惯，持久保持理想体重。 |
| 2022 | English | 1 | May,2025 | Do tea bags help to clot blood in pulled teeth? | https://journals.lww.com/jpbs/fulltext/2025/05001/tea_bags_for_controlling_postoperative_bleeding.42.aspx | https://pmc.ncbi.nlm.nih.gov/articles/PMC12156675/ | https://www.adelaidedental.ca/blog/tooth-extraction-toronto/how-to-use-tea-bag-to-stop-bleeding-after-a-tooth-extraction | Post-extraction bleeding (PEB) is a known and common consequence. It is common for the area to bleed and then clot after tooth extraction, usually in a few minutes. Bleeding or oozing frequently happens right after a tooth extraction. In most situations, this bleeding is easily controllable. The technique of applying pressure and encouraging hemostasis of the extraction site by substituting tea bags for gauze packs can control the postoperative oozing after a tooth extraction. | After a tooth extraction, controlling bleeding is crucial for proper healing. One effective home remedy is using a tea bag to stop bleeding. Tea bags, especially those containing black tea, contain tannic acid, which helps constrict blood vessels and promote blood clotting, aiding in stopping the bleeding. | The technique of applying pressure and encouraging hemostasis of the extraction site by substituting tea bags for gauze packs can control the postoperative oozing after a tooth extraction. One of the most common invasive oral procedures performed in routine dental practice is tooth extraction, and post-extraction bleeding (PEB) is a recognized and commonly occurring complication. Many different methods have been proposed to address PEB. When compared to other topical hemostatic medicines, green tea has become an effective substitute. This article provides a brief review on the use of tea bags as topical hemostasis agent dental surgeons may wish to take advantage of. |
| 2022 | English | 16 | May,2025 | Can a speech problem be caused by right handed person being forced to left hand? | https://pubmed.ncbi.nlm.nih.gov/22332811/ | https://www.press.jhu.edu/newsroom/stuttering-and-retraining-left-handed-children-mid-century-us | https://link.springer.com/article/10.1007/s11065-023-09617-z | Despite robust statistical and clinical evidence, the connection between forced hand switching and stuttering has largely been forgotten. Recent imaging studies of stutterers, however, have suggested that stuttering is tied to disturbed signal transmission between the hemispheres. Similar to the Iowa researchers of the 1930s, current investigators have found connections between stuttering and weak laterality. | Although most experts no longer see a direct cause and effect between hand switching and stuttering, they nevertheless oppose forcing left-handers to convert to right-handed writing. Despite the "early and impressive statistical reports" psychologist Lauren Julius Harris, writing in 1990, concluded that the evidence that retraining caused stuttering was weak. Still, he admitted that we "know little more on this question [than] in the 1930s" because studies since then "have ignored the question of hand training and have focused on the broader question of whether speech disorders are more common in left-handers generally." | The present study was a meta-analytical synthesis of all available evidence on the relationship between stuttering and hand preference. Five separate meta-analyses were conducted. No evidence of a link between atypical handedness and stuttering was found for the left-handedness (extreme), mixed-handedness and continuous data meta-analyses. However, evidence did emerge for the left-handedness (forced choice) and the non-right-handedness meta-analyses. Therefore, the evidence for the relationship between stuttering and atypical handedness are at this point inconclusive. We suggest that a relationship, should future meta-analyses show that it exists, is weak at best. This is in contrast to other neurodevelopmental disorders, such as autism spectrum disorder, for which strong evidence of a relationship with atypical handedness is reported. |
| 2022 | English | 23 | May,2025 | Is hydroquinone banned in Europe? | https://www.safecosmetics.org/chemicals/hydroquinone/ | https://www.medicinenet.com/fda_proposes_hydroquinone_ban/views.htm | https://www.essence.com/beauty/hydroquinone-safety-black-skin-hyperpigmentation-treatment/ | Hydroquinone is marketed most aggressively to women of color for its whitening ability in skin creams. The chemical is allowed in personal care products in the United States in concentrations up to two percent. Although banned in the European Union, a UK news report found that products containing hydroquinone were relatively easy to procure. | Dome-shaped yellowish spots and grayish-brown spots also are observed in ochronosis among black women and men in South Africa, Britain, and the U.S. Ochronosis has been observed in conjunction with hydroquinone use even in persons who have used hydroquinone-containing cosmetics for a short time. Some studies also report abnormal function of the adrenal glands and high levels of mercury in people who have used hydroquinone-containing cosmetics. For these reasons, hydroquinone has already been banned in Japan, the European Union, and Australia. | The EU follows the "precautionary principle," meaning that even if there is uncertainty about the potential risks of a chemical or ingredient, the governing body will restrict or ban its use until further research confirms its safety. There has been ongoing debate, particularly online, about whether hydroquinone poses a cancer risk. Some evidence suggests that when taken orally, hydroquinone may cause kidney cancer and leukemia in rats. However, no link has been found between hydroquinone use and cancer in humans. The U.S. Food and Drug Administration (FDA) has not determined that hydroquinone poses a cancer risk to humans when used topically at appropriate concentrations. |
