# Supplementary material for: Bilingual Performance of Large Language Models in Answering Consumer Health Questions in English and Chinese: Comparative Benchmark Study
Source: J Med Internet Res. 2026 Sep 29;28:e96587. doi: 10.2196/96587 (PMC13662449; doi:10.2196/96587)
Supplement: Supplementary file 14 [file jmir_v28i1e96587_app7.docx]

# **Multimedia Appendix 7. TRIPOD-LLM Reporting Checklist**

| **Section** | **Item** | **TRIPOD-LLM checklist item** | **Reported in manuscript / reporting status** |
| --- | --- | --- | --- |
| Title | 1 | Identify the study as evaluating LLM performance, specifying the task, target population, and outcome. | Title (evaluation study and consumer-health question-answering task identified). |
| Abstract | 2 | Structured abstract covering items 2a-2l below. | Abstract; see items 2a-2l below. |
| Abstract: Research design | 2a | Identify study as evaluating LLM performance. | Title; Abstract, Objective. |
| Abstract: Background | 2b | Healthcare context, use case, and rationale. | Abstract, Background. |
| Abstract: Objectives | 2c | Study objectives. | Abstract, Objective. |
| Abstract: Methods | 2d | Key elements of study setting. | Abstract, Methods. |
| Abstract: Methods | 2e | All data used; data splits. | Abstract, Methods (single evaluation dataset; no training/tuning split performed). |
| Abstract: Methods | 2f | Name and version of LLM(s) used. | Abstract, Methods (11 models named); Methods, Study Design; Supplementary Table 1. |
| Abstract: Methods | 2g | LLM-building steps, fine-tuning, RLHF. | NA - existing pretrained models were evaluated as available; no author-led model building or fine-tuning. |
| Abstract: Methods | 2h | Specific tasks performed by LLMs. | Abstract, Methods (binary classification plus explanatory consumer-health answers) |
| Abstract: Methods | 2i | Evaluation datasets, endpoints, measures. | Abstract, Methods (TREC 2019/2021/2022; accuracy and communication-quality metrics). |
| Abstract: Results | 2j | Overall report of main results. | Abstract, Results. |
| Abstract: Discussion | 2k | Broader implications. | Abstract, Conclusions. |
| Abstract: Other | 2l | Registration number/repository. | NA - the study was not preregistered; no registration number. |
| Introduction: Background | 3a | Healthcare context and rationale for evaluating the LLM. | Introduction, Background. |
| Introduction: Background | 3b | Target population and intended use of the LLM. | Introduction and Discussion. |
| Introduction: Objectives | 4 | Study objectives; whether development, fine-tuning, or evaluation. | Introduction, final paragraph. |
| Methods: Data | 5a | Sources of data for evaluation, and rationale. | Methods, Question Set and Bilingual Preparation; Supplementary File 1. |
| Methods: Data | 5b | Data points, distribution, and descriptors. | Methods, Question Set and Bilingual Preparation (150 questions; 51/49/50 by year; English and Chinese); Supplementary File 1. |
| Methods: Data | 5c | Date of oldest and newest text used. | Methods, Study Design and Question Set (evaluation texts: 2019-2022; model training dates unavailable where developers do not disclose them). |
| Methods: Data | 5d | Data preprocessing and quality checking. | Methods, Question Set and Bilingual Preparation (selection, translation, back-translation, adjudication, and quality review). |
| Methods: Data | 5e | Missing and imbalanced data handling. | NA. |
| Methods: Analytical | 6a | LLM name, version, last training date. | Methods, Study Design; Supplementary Table 1 (model/version/access date). |
| Methods: Analytical | 6b | LLM architecture, training, fine-tuning, alignment. | NA - existing models were evaluated as available; no model development, fine-tuning, or alignment was performed by the authors. |
| Methods: Analytical | 6c | Text generation details: prompt engineering, inference settings. | Methods, Prompting Strategy; Supplementary File 3 (prompts and available interface settings). |
| Methods: Analytical | 6d | Initial and postprocessed output of the LLM. | Methods, Prompting Strategy and Evaluation Metrics (raw free-text responses, yes/no outcome extraction, and scoring; no additional model-output postprocessing). |
| Methods: Analytical | 6e | Classification details and thresholds. | Methods, Evaluation Metrics (binary classification rule and ordinal scoring scales). |
| Methods: LLM output | 7a | Metrics capturing quality of outputs vs gold standards. | Methods, Evaluation Metrics (accuracy, comprehensiveness, precision, and understandability). |
| Methods: LLM output | 7b | Outcome metrics' relevance to downstream task. | Methods, Evaluation Metrics; Discussion. |
| Methods: LLM output | 7c | Outcome definition, prediction calculation, date of inference. | Methods, Study Design (inference conducted May 1-31, 2025); Methods, Evaluation Metrics and Statistical Analysis. |
| Methods: LLM output | 7d | Assessor qualifications, instructions, inter-assessor agreement. | Methods, Evaluation Metrics (two bilingual clinical reviewers, scoring instructions, and inter-rater agreement). |
| Methods: LLM output | 7e | How performance was compared to other LLMs/benchmarks. | Methods, Statistical Analysis (comparison across 11 LLMs using GEE, LMM, and TOPSIS); no human-performance benchmark. |
| Methods: Annotation | 8a | How text was labeled; annotation guidelines with examples. | Methods, Evaluation Metrics; Supplementary File 4 (scoring rubric with anchors/examples). |
| Methods: Annotation | 8b | Number of annotators; inter-annotator agreement. | Methods, Evaluation Metrics (two reviewers; all responses independently rated; kappa statistics); Supplementary Table 3. |
| Methods: Annotation | 8c | Background and experience of annotators. | Methods, Evaluation Metrics (bilingual clinical background of ZW and HX); add roles/experience if available. |
| Methods: Prompting | 9a | Prompt design, curation, and selection. | Methods, Prompting Strategy; Supplementary File 3. |
| Methods: Prompting | 9b | Data used to develop prompts. | Methods, Prompting Strategy (prespecified benchmark prompts; no separate prompt-development dataset). |
| Methods: Summarization | 10 | Preprocessing before summarization. | NA – question answering/classification study; no summarization task. |
| Methods: Instruction tuning | 11 | Instruction tuning/alignment strategies. | NA - no instruction tuning or alignment was performed by the authors. |
| Methods: Compute | 12 | Compute requirements or proxies thereof. | Methods, Study Design - models were accessed through public web interfaces; provider-side compute/GPU information was unavailable, and no local training, fine-tuning, or investigator-controlled inference hardware was used. Add elapsed-time/cost proxies if collected. |
| Methods: Ethical approval | 13 | IRB/ethics committee approval; informed consent. | Methods, Ethical Considerations (IRB exemption No. I-26ZM0094; no human participants or patient data). |
| Methods: Open science | 14a | Funding source and role of funders. | Funding Statement lists funding sources. |
| Methods: Open science | 14b | Conflicts of interest. | Competing Interests / Conflict of Interest statement. |
| Methods: Open science | 14c | Study protocol access. | No study protocol was prepared - state this explicitly in the final manuscript. |
| Methods: Open science | 14d | Registration information. | The study was not registered - state this explicitly if the item is retained. |
| Methods: Open science | 14e | Data availability. | Data Availability (public TREC Health Misinformation Track datasets; add direct dataset links/access details where possible). |
| Methods: Open science | 14f | Code availability. | Code Availability (GitHub repository: https://github.com/wuziyanalice/LLM_HMT). |
| Methods: Public involvement | 15 | Patient and public involvement. | NA - no patient or public involvement. |
| Results: Participants | 16a-d | Patient/EHR data flow and characteristics. | NA - no patient/EHR data or clinical outcomes; evaluation used publicly available consumer-health questions. |
| Results: Performance | 17 | LLM performance per prespecified metrics. | Results; Tables 1-3; Figures 2-6; Supplementary analyses. |
| Results: LLM updating | 18 | Results from any LLM updating. | NA - no LLM updating, tuning, or model modification was performed. |
| Discussion: Interpretation | 19a | Overall interpretation in context of objectives. | Discussion, interpretation of principal findings. |
| Discussion: Limitations | 19b | Study limitations and effects on biases. | Discussion, Limitations. |
| Discussion: Usability | 19c | Challenges in using data for specified task. | Discussion, Limitations (representation, translated questions, search-engine effects, ground truth, temporal drift, and language generalizability). |
| Discussion: Usability | 19d | Intended use, end-user, level of autonomy. | Discussion and Conclusions. |
| Discussion: Usability | 19e | Handling poor quality or unavailable input data. | Method. |
| Discussion: Usability | 19f | User expertise required. | Partially reported - state that users enter questions through public web interfaces, that no specialized technical expertise is required, and what clinical/health-literacy oversight is appropriate. |
| Discussion: Usability | 19g | Next steps for future research. | Discussion, future directions and Conclusions (open-ended tasks, harm classification, low-resource languages, and broader validation). |
