# Supplementary material for: Bilingual Performance of Large Language Models in Answering Consumer Health Questions in English and Chinese: Comparative Benchmark Study
Source: J Med Internet Res. 2026 Sep 29;28:e96587. doi: 10.2196/96587 (PMC13662449; doi:10.2196/96587)
Supplement: Supplementary file 15 [file jmir_v28i1e96587_app8.docx]

| **Multimedia Appendix 8. Inter-rater Reliability: Cohen Kappa and Weighted Kappa Values** | | |
| --- | --- | --- |
| Metric | Kappa type | κ value |
| Accuracy | Cohen's κ | 0.82 |
| Precision | Weighted κ (quadratic) | 0.82 |
| Comprehensiveness | Weighted κ (quadratic) | 0.80 |
| Understandability | Weighted κ (quadratic) | 0.81 |
